# Additive Manufacturing of Nanoscale Multimaterial Voxels Via Meniscus-Confined Electrodeposition

**DOI:** 10.1021/acsnano.5c16931

**Published:** 2026-04-08

**Authors:** Simon Sprengel, Julian Hengsteler, Peng Zeng, Albert Ripoll Oliveras, Muhammad Zerehi Zadeh, Weishan Wu, Andrei Zotov, Daniel Torres, Xinhua Zhu, Jon Ustarroz, Vita Solovyeva, Tomaso Zambelli, Dmitry Momotenko

**Affiliations:** † Laboratory of Electrochemical Nanotechnology, Institute of Chemistry, 11233 Carl von Ossietzky Universität Oldenburg, Oldenburg D-26129, Germany; ‡ Laboratory of Biosensors and Bioelectronics, Institute for Biomedical Engineering, 27219 ETH Zürich, Zurich CH-8092, Switzerland; § 27219 ETH Zürich, The Scientific Center for Optical and Electron Microscopy (ScopeM), 8093 Zurich, Switzerland; ∥ Chemistry of Surfaces, Interfaces and Nanomaterials (ChemSIN), 26659 Université libre de Bruxelles, 1050 Brussels, Belgium; ⊥ Electrochemical and Surface Engineering (SURF), Vrije Universiteit Brussel, 1050 Brussels, Belgium; # 11233 Carl von Ossietzky Universität Oldenburg, Fakultät V, Institut für Physik, Oldenburg 26129, Germany

**Keywords:** Nanoprinting, 3D printing, nanopipette, MCED, alloy

## Abstract

Advanced applications featuring sub-microscale and nanoscale metallic structures, which include energy storage devices, nanophotonic elements, and nanoelectronic interfaces, require three-dimensional multimaterial structural elements. Here, we present an approach for highly localized meniscus-confined electrodeposition based on double-barrel nanopipettes capable of producing high-aspect ratio metallic structures with a wide range of elemental compositions. This is enabled by the possibility of finely tuning local ionic content directly inside the liquid meniscus by applying voltage bias between the barrels filled with different electrolytes. This provides a platform for fast switching between materials within a single voxel and the fabrication of smooth material gradients via tunable electrodeposition, which is also characterized by improved mass-transport and faster print rates. We demonstrate the capability of this approach by producing various arrangements of Cu–Au and Au–Pt voxels with ca. 200 nm lateral resolution, which are formed from fully dense (non-porous) polycrystalline metallic alloys with the evidence of metastable microstructural features.

## Graphical abstract

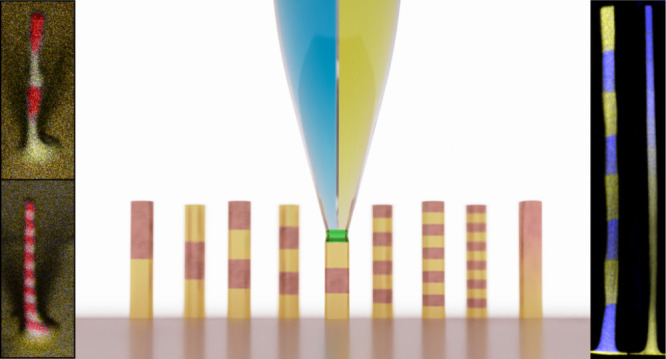

Additive manufacturing (AM), commonly known as 3D printing, is a family of techniques to produce real-world objects from a digital model.^1^ Enabled by computer-aided design tools and recent advances in materials science, AM has allowed processing a wide range of materials, allowing manufacturing of objects as diverse as jet engines^2^ and reactors for chemical industry,^3–5^ to buildings,^6^ implants,^7^ and electronic components.^8^ The advantages of predominantly single-step and three-dimensional (3D) fabrication by AM manifest themselves the strongest at the nanoscale, where processes are based on mostly sequential and, by definition, planar technologies, like photolithography. Despite recent advances, a particular challenge for AM at small dimensions remains in processing multiple materials simultaneously. This will expand fabrication capabilities by the potential to create sacrificial support layers^9^ that can help to achieve close to unlimited design complexity; ultimately, multimaterial capacity should also allow fabrication of functional interfaces and devices. The wide application span for these technologies includes state-of-the-art examples, like racetrack memory devices,^10^ spin-filters,^11^ and functional elements for quantum computing,^12^ nanoelectronic elements,^13^ microbatteries,^14^ and nanorobots.^15^

There is a range of small-scale AM techniques that already exhibit a capacity to produce multimaterial structures. For example, direct laser writing based on microfluidic delivery of photopolymerizable precursors allowed sequential printing of complex-shaped structures built from multiple components.^16,17^ 3D-nanoprinting in this case can be performed with a large number of precursors (demonstrated with up to five), given by the flexibility of the microfluidic delivery system, which, however, makes switching between materials possible only at relatively low frequency due to the need to refill the printing space with the appropriate monomer. Another set of techniques is based on focused electron beam-induced deposition (FEBID) that achieves metal deposition from a precursor gas, delivered into the vacuum environment of an electron microscope. Magnetic 3D-structures with nanoscale dimensions composed of FeCo and Pt segments were demonstrated,^18^ although FEBID techniques always suffer from some carbon contamination in the structures and require (ultra)­high vacuum equipment. AM methods capable of processing more pure metals typically suffer from resolution capabilities. For example, laser-induced forward transfer (LIFT), which manipulates metal droplets ejected from a sacrificial support onto the target substrate, was shown to print Au–Pt microstructures.^19^ There are also other emerging techniques capable to manipulate metallic nanoparticles by focused electric fields, which have been shown to be capable of fabrication of multimetal (Pd, Au, Ag, and Pt) microstructures,^20^ although the shape of the deposits is not of arbitrary complexity. These impressive advances highlight that processing of electrically conductive features with user-defined shapes and controlled composition—these relevant to real-world applications—remains elusive.

In contrast, electrochemical AM (e-AM) techniques, which are based on highly confined electrodeposition, offer almost unlimited design freedom,^21^ nanoscale resolution,^22^ and high electrical conductivity.^23^ While there are several materials reported suitable for printing metals, including platinum,^24^ silver,^25,26^ nickel,^26,27^ or copper–nickel alloys by co-electrodeposition,^28^ the multimaterial capacity of e-AM remains limited. For example, double-barrel nanopipettes have been used in multimetal electrohydrodynamic redox printing (EHD-RP).^25^ Each barrel was equipped with a sacrificial metal anode of Cu and Ag, thus allowing switching of the deposition between these two metals by applying the voltage to one or another metal wire. Fabrication of an alloy (mixture) of both metals or varying composition with EHD-RP is not trivial, and while alloys can be achieved by modifying the composition of electrolyte,^29^ manipulation of the chemical content of the printed structures on the fly has not been demonstrated. This is in part possible by tuning the electrodeposition conditions (e.g., voltage, current, relative humidity) in a meniscus-confined electrodeposition (MCED) configuration. Such nanopipette-based techniques currently are not flexible enough to vary the composition of the printed structures on-the-fly.

Here, we demonstrate a different approach for multimaterial e-AM based on MCED, an e-AM technique capable to deposit metals and alloys.^30^ By driving ionic mass-transport through a nanoscale meniscus that connects the barrels of a theta-nanopipette in a controllable way, the print process that features 200 nm resolution results in a widely variable material composition of the fabricated structures which can be adjusted on-the-fly and at subvoxel level. Comprehensive material analysis of the printed structures confirms high dynamics of the process and reveals the possibility to fabricate solid solutions, which show unusual single-phase alloy behavior, unachievable otherwise due to the existence of a miscibility gap.

## Results and Discussion

### Working Principle

In a conventional MCED the print process occurs due to electrodeposition of metal at the area of the substrate wetted by a liquid meniscus that exists between the nozzle tip and the interface of the substrate electrode (working electrode, WE).^31^ The electrode potential that drives the electrodeposition is controlled by the difference in voltage between the WE and quasi-reference counter electrode (QRCE) wire inside the electrolyte-filled nanopipette, containing ions of metal precursor that undergo electrochemical reduction at the WE interface. In our case, the e-AM process is implemented with positional feedback of an intermittent MCED (iMCED) that drives the nozzle toward the substrate until a meniscus is established and then immediately withdraws the nanopipette until no Faradaic current is detected.^22^ These periodic cycles of meniscus formation and breaking enable to precisely monitor the height of the grown voxel (typically, within sub-nm to single-digit nm), estimate its diameter on-the-fly, and, most importantly, prevent nozzle clogging as the nozzle retraction then becomes automatically adjusted to the metal growth. Additionally, the stability of the meniscus during printing, which is critical for conventional MCED, becomes much less of a problem in iMCED because the lifetime of a meniscus is limited to only several milliseconds on each cycle.

For multimaterial e-AM we employed iMCED with a double-barrel nanopipette nozzle (Figure 1, further details in the Supporting Information SI-1), where each compartment is filled with its own electrolyte and is equipped with its own QRCE. Here, Pt wire is chosen as the QRCE material in order to avoid possible contamination of the electrolyte and deposition of unwanted metal on the substrate, which can become an issue with other materials, like Ag/AgCl.^32^ This configuration is highly advantageous, as it allows controlling the chemical composition of the ionic species inside the meniscus via electrophoresis as well as simultaneously adjusting the deposition potential on the substrate. The former is possible since the ion transport is driven by the voltage bias between the barrels (with voltages defined as *V*_1_ and *V*_2_). The bias *V*_*bias*_

$$V_{bias}=V_{1}-V_{2}$$

determines the driving force to populate the meniscus with various ionic species. More specifically, when depositing metal ions of opposite polarity, such as positively charged copper (Cu^2+^) and negatively charged gold (AuCl_4_^–^), both salts have to be contained in one of the barrels, whereas the other nozzle compartment is filled only with the supporting electrolyte. In this case a positive bias (*V*_1_ *> V*_2_) leads to movement of the positive ions from barrel one through the meniscus into barrel two, causing enhancement of the concentration of cation precursor in the meniscus space (Cu^2+^, Figure 1b, panel (i)). For a negative bias (*V*_1_ *< V*_2_), ion transport leads to an increase of the concentration of negatively charged ions (AuCl_4_^–^) in the meniscus (Figure 1b, panel (ii)). Conversely, for metal ions with similar polarity, such as negatively charged gold (AuCl_4_^–^) and platinum (PtCl_6_^2–^), two metal-containing electrolytes are located in opposite nozzle barrels. In this case, change of the bias polarity would draw one or another anion into the meniscus (Figure 1b, panels (iii) and (iv)).

**Figure 1:**
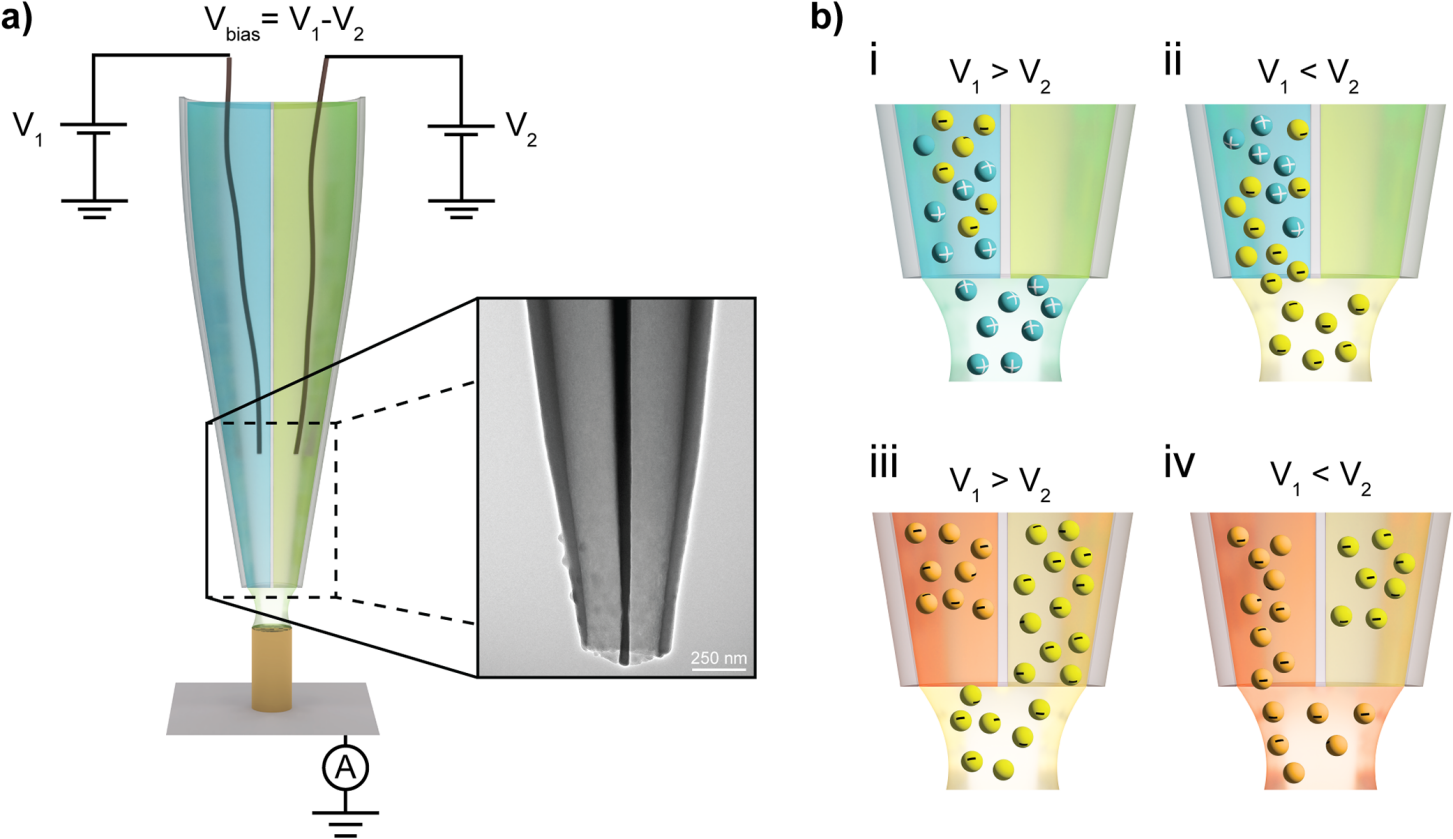
Schematic of the multimetal printing concept with double-barrel nanopipette nozzles. (a) An illustration of the theta nanopipette, filled with different electrolyte solutions (in each barrel) and equipped with QRCEs to control voltage difference across the meniscus as well as the electrochemical substrate potential. The multimaterial printing capacity is allowed due to the different ionic composition inside the nozzle barrels and the voltage difference between QRCEs, which allows to dynamically change ionic content inside the liquid meniscus, where electrodeposition takes place. The inset in (a) shows a TEM image of a typical nozzle employed for printing. (b) Schematic showing changes in ionic composition of the electrolyte inside the meniscus due to the applied voltage bias. Panels (i) and (ii) show the operation with metal ions of opposite polarities, where the ions move from the barrel on the left toward the barrel on the right that contains only supporting electrolyte. Under a positive bias voltage (V_1_ > V_2_) the meniscus is filled with cations, and under a negative bias voltage (V_1_ < V_2_) anions dominate the meniscus. Panels (iii) and (iv) illustrate printing with ions of similar polarity that are contained in separate nozzle barrels. Voltage bias drives the metal precursors from right to left or left to right, depending on the electric field direction.

The control of the deposition potential is somewhat more complicated than in the case of conventional MCED, where the substrate voltage needs to be set simply with respect to a single QRCE. Here, the presence of two QRCEs requires a more detailed consideration, since in a general case the substrate potential (*E*_*sub*_) has to be referenced with respect to both of them via

$$E_{sub}=-\left(\alpha_{v}V_{1}+\left(1-\alpha_{v}\right)V_{2}\right)$$

where α_*v*_ is a coefficient that determines which of the QRCEs has higher influence on the substrate potential. This coefficient is mainly determined by the asymmetry in resistance between the QRCEs, given by the asymmetry in geometry of the nozzle barrels, a common occurrence when laser-pulling, as well as differences in electrolyte conductivity. For a perfectly symmetrical nanopipette nozzle with a matched conductivity in each barrel α_*v*_ is 0.5, and the potential at the substrate is then given by

$$E_{sub}=-\frac{V_{1}+V_{2}}{2}$$

In a fully asymmetric case with α_*v*_ = 1 or α_*v*_ = 0, the substrate potential would then simply become either *V*_1_ or *V*_2_, respectively, making voltage control similar to single-barreled MCED. Following these considerations, one can control the deposition voltage and the bias independently. For example, it is possible to keep fixed *V*_*bias*_ and adjust deposition voltage by simultaneously increasing or decreasing both *V*_1_ and *V*_2_, or, conversely, keep a desired *E*_*sub*_ and then adjust *V*_*bias*_ to the desired value. Our finite element simulations demonstrate that accurate control of fluxes and potentials is indeed possible regardless of the asymmetry of the electrolyte composition and conductivity; however, printing parameters should be adjusted for each case (Supporting Information SI-2).

### Printing with Sub-voxel Material Control

When a sufficiently high bias is applied, one of the two metal ions becomes dominant in the meniscus and, subsequently, is preferentially printed onto the substrate. Change of the bias polarity allows switching between the metals (Figure 2). First, we tested this principle with Cu and Au, a case where precursor species have opposite polarities (Cu^2+^ and AuCl_4_^–^). The left pipette barrel (barrel 1) was filled with 10 mM CuSO_4_, 10 mM HAuCl_4_, and 500 mM H_2_SO_4_, whereas the right (barrel 2) contained 10 μM CuSO_4_, 10 μM HAuCl_4_, and 507.6 mM H_2_SO_4_. The electrolyte concentrations in the second barrel were adjusted to match the conductivity of the first barrel and allow the establishment of a well-defined voltage offset. Symmetric conductivities in both electrolytes allow a simplified approach to estimate the *E*_*sub*_ (which becomes similar to a commonly used assumption of the WE potential in scanning electrochemical cell microscopy,^33^ an approach that allows nanoscale electrochemical imaging as well as electrodeposition of nanoparticles on planar substrates^34^).

**Figure 2:**
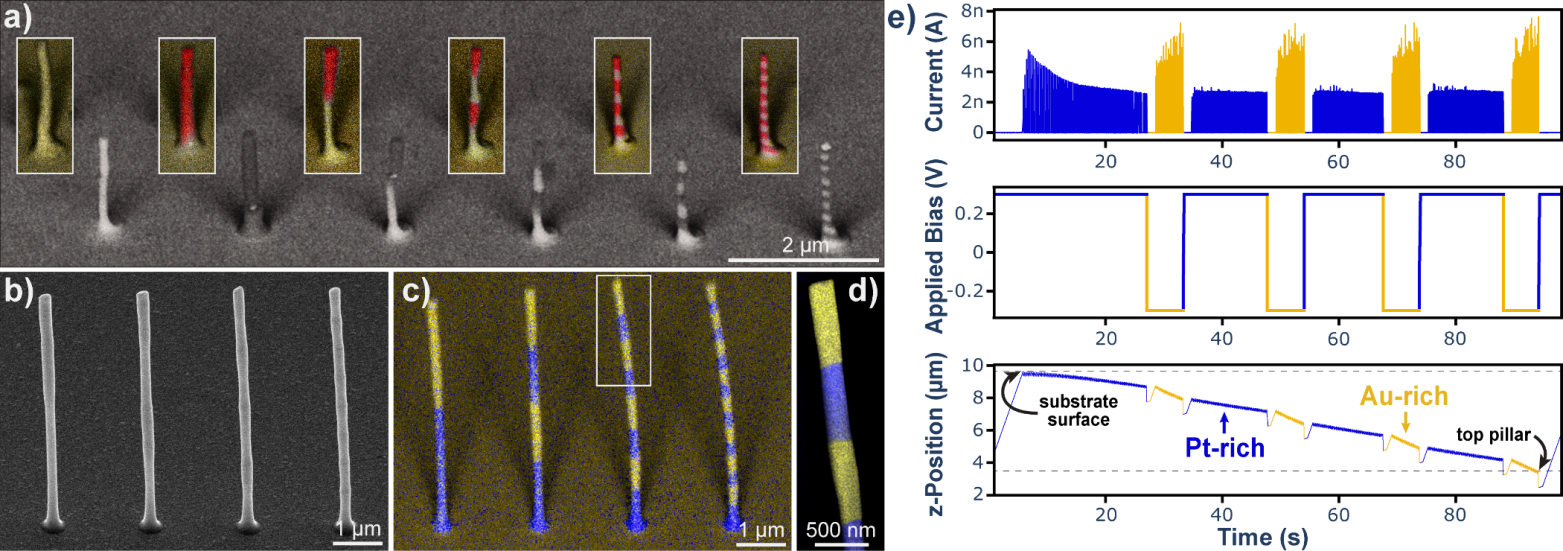
Printing with sub-voxel material control. **a)** Backscattered electron microscopy image with a partly overlaid EDX map of “zebra-striped” pillar structures with enriched Au (bright EM contrast/yellow intensity in EDX) and Cu (dark EM contrast/red intensity in EDX, overlay at same scale as BSE image) metal content achieved by switching between voltage bias between the nozzles ± 0.7 V and printed at constant substrate potential of −1.1 V. **b)** SEM image of metal pillars containing Pt- and Au-rich segments, printed at biases of ± 0.3 V and substrate potential of −0.99 V for Au and −0.81 V for Pt. **c)** EDX map of the structures shown in (b) with Au and Pt enriched pillar segments colored in yellow and blue, respectively. **d)** A close-up view of the marked section in (c) showing sharp boundaries between Au and Pt segments. **e)** A snapshot of a print process data for Au–Pt pillar consisting of 8 segments, showing the variations of electrodeposition current (top), applied bias (middle), and vertical z-piezo position (bottom). The latter shows retraction of the nozzle upon pillar growth. The colors of the data (yellow and blue) correspond to printing Au and Pt.

To demonstrate the influence of bias voltage on printing, both materials were deposited at the same *E*_*sub*_ of −1.1 V and *V*_*bias*_ of ±0.7 V for Cu and Au, respectively. Individual material sections of the fabricated pillar structures with alternating material sections in a “zebra-striped” pattern are very well visible both in backscattered electron (BSE) microscope images and in the energy dispersive X-ray (EDX) map overlay shown in Figure 2a. The contrast in BSE image arises from differences in the backscattered electron coefficient, which increases with the atomic number (Z), which manifests itself in brighter contrast for elements with higher Z, such as gold (Z = 79), since they produce more backscattered electrons than lighter ones (Z = 29 for Cu). The height of the individual material sections (set values defined by the user) from left to right varied from 1000 to 500, 250, and 125 nm. Given that the pillars are fabricated with a diameter of about 149 ± 9 nm (confidence interval for 95% confidence level, CI_95_, number of samples n = 21), the material switching is evidently feasible even at a sub-voxel scale. The EDX overlay confirms the material distributions, with Cu and Au intensities colored red and yellow, respectively. EDX analysis also confirms the elemental composition, featuring average composition (CI_95_, n = 29) of Au sections at 51.9 ± 2.9 at% Au and 48.1 ± 2.9 at% Cu, and 30.4 ± 2.3 at% Au with 69.6 ± 2.3 at% Cu for Cu sections ().

Gold, as a more noble metal, has a significantly higher electrodeposition potential (*E* ^0^ of 1.002 V vs 0.342 V for Cu vs standard hydrogen electrode, SHE),^35^ making the driving force, i.e., the overpotential, for Au reduction significantly higher. However, the data show favored Cu deposition over Au (70 at% Cu in Cu-sections vs 52 at% Au in Au-sections). This is due to differences in deposition kinetics, with Cu growth rates being faster than those of Au. Cu deposition from a sulfate electrolyte proceeds via a relatively simpler two-step electron transfer,^36,37^ whereas Au deposition from a chloride salt typically occurs through stepwise pathways (*AuCl*_4_^–^ → *AuCl*_2_^–^ → *Au*) and is often associated with sluggish charge-transfer kinetics and inhibition phenomena, also due to the strength of the *Cl* ^–^ ligand.^38,39^ As an example, under comparable conditions of overpotential and bath composition, reported Tafel slopes are between 0.165 and 0.130 V dec^–1^ for Cu^40^ and between 0.33 – 0.39 V dec^–1^ for Au deposition.^41,42^ In any case, our results indicate that it is possible to switch on-the-fly between different material compositions at various length scales at a fixed substrate potential.

Demonstration of iMCED using the second configuration, which employs metal ions of similar polarity, was shown by printing Au and Pt using AuCl_4_^–^ and PtCl_6_^2–^, respectively. Here, the left compartment of the theta pipette was filled with 10 mM HAuCl_4_, while the right compartment contained 110 mM H_2_PtCl_6_. A significant excess of Pt precursor served to allow a higher percentage of Pt in the resulting structures, which otherwise is difficult to achieve due to sluggish Pt deposition kinetics (vide infra). The printing experiment was carried out at voltages of *V*_1_ = 1.05 V (or 0.75 V) and *V*_2_ = 0.75 V (or 1.05 V) establishing a *V*_*bias*_ of +0.3 V (or −0.3 V) for driving either Pt or Au precursor into the meniscus. Given the asymmetric conductivity of the system, *E*_*sub*_ was estimated using simulated coefficient α of ca. 0.2 (Supporting Information SI-2), resulting in *E*_*sub*_ of −0.99 V for Au-deposition and −0.81 V for Pt-deposition. This is sufficient to electrodeposit both metals, although slightly less ideal for printing Pt, which deposits at the highest rate at −0.95 V vs Pt QRCE. The alternating Au and Pt sections of 4000, 2000, 1000, and 500 nm in height (set-values) formed 8 μm-tall pillars (Figure 2b). Despite the BSE image exhibits practically no intensity contrast between the metals due to very close Z between Pt and Au (78 for Pt vs 79 for Au), the segments can be differentiated via a noticeable variation in the diameter. For Au segments, the pillar diameter (CI_95_, n = 4) reaches 247 ± 10 nm, which is thicker than that of Pt (203 ± 10 nm). Elemental composition shown in Figure 2c confirms that the variation in pillar thickness is indeed related to different chemical compositions of individual segments. The average material composition (CI_95_, n = 15) taken from a close-up high-resolution EDX image shown in Figure 2d confirms strong (over 26 at%) variation between chemical content of the sections, with 85.5 ± 0.6 at% of Au and 40.8 ± 1.6 at% of Pt in the gold-rich and platinum-rich sections, respectively (Supporting Information SI-4). In this case, gold (1.002 V vs SHE)^35^ also has a higher electrodeposition potential than platinum (0.755 V vs SHE)^35^ making the driving force for Au reduction significantly higher, together with the fact that Pt deposition from PtCl_6_^2–^ is known to suffer from very sluggish kinetics.^43^ Therefore, in this case both thermodynamics (overpotential) and deposition kinetics favor Au deposition over Pt, even more than Cu is favored over Au.

Elemental characterization shows a pattern comparable to the Cu/Au system (Figure 2a) with a sharp transition of chemical content between Pt and Au segments (Figure 2c), which takes place, most likely, within a single iMCED layer (vide infra). This is in a good agreement with the print data shown in Figure 2e, which exhibits transient profiles of electrodeposition current, applied bias, and absolute position of the vertical piezo nanopositioner during printing of the 8-sectioned pillar (). The profiles show consistent and very sharp changes in the electroplating current, characteristic for each metal, and demonstrate a clear difference in electrodeposition rates (slope of the z-position curve) between Pt and Au, with the latter being printed significantly faster (57 versus 159 nm s^–1^). The data also demonstrates reproducibility of the process, with stable electrodeposition currents of ca. 3 nA for Pt and 5–7 nA for Au. Higher current levels are consistent with the deposited layer heights of the iMCED, which are correspondingly larger for Au (32.6 nm/layer) than for Pt (10.2 nm/layer) sections for a comparable meniscus existence time (ca. 11 ms). In case of the Au-rich section (86:14 alloy), the deposition exhibits almost 100% Faradaic efficiency, as determined by comparison of the measured growth rate with the measured current. In contrast, for the Pt-rich section (59:41 alloy) the Faradaic efficiency is much lower (ca. 52%). Since the process takes place at the potential of −0.81 V vs Pt wire, the current under these conditions is significantly affected by the contribution from hydrogen evolution reaction, making further comparative kinetic analysis of the transients difficult. It is worth highlighting here that we intentionally disregarded the symmetry of the conductivity between the barrels such that the substrate electrode potential is almost entirely determined by the voltage difference with the QRCE in the barrel with PtCl_6_^2–^, which nevertheless does not compromise the print process.

Important to note, printing with a single-barrel iMCED using a mixed electrolyte of similar concentration (10 mM HAuCl_4_ and 110 mM H_2_PtCl_6_) results in a similar composition of the features, with the highest concentration of Au and Pt around 97 at% and 37 at% achieved by varying *E*_*sub*_ between −0.4 V and −1.00 V. This approach, however, is significantly less practical than bias-controlled iMCED with a double-barrel nozzle due to up to an order of magnitude slower print rates, achieved due to improved bias-driven mass transport of ions through the meniscus ().

### Control of Chemical Composition

The bias between the barrels is a dial that can be tuned to achieve the desired composition of metals in the printed structure (within a certain composition range) and thus deposit alloys with tunable material ratios. To demonstrate this capability, an array of 12 Au/Pt pillars was printed at different *V*_*bias*_, from −0.3 V to +0.3 V with a step of 0.05 V (skipping 0 V), hence from Pt-rich to Au-rich metal content (Figure 3a). The EDX analysis of the printed features demonstrates the resulting effect on chemical composition for each corresponding pillar, with the platinum content showing a continuous decrease from about 63 at% to about 10 at%. Interestingly, the single composition array yields a larger range of Pt amount with higher maximum Pt content of 63 at% and at the same time lower minimum Pt content of 10 at% recorded with lower-resolution SEM-EDX compared to the “zebra-striped” pillar resulting in a high Pt content of 41 at% and low Pt content of 14 at%. We attribute this to somewhat different switching conditions in these experiments: for “zebra-striped” pillars the bias profile changed rapidly and sharply (5–15 s to change bias ±0.3 V), whereas only slight bias changes took place for the single pillars (365 s between the highest and the lowest *V*_*bias*_).

**Figure 3:**
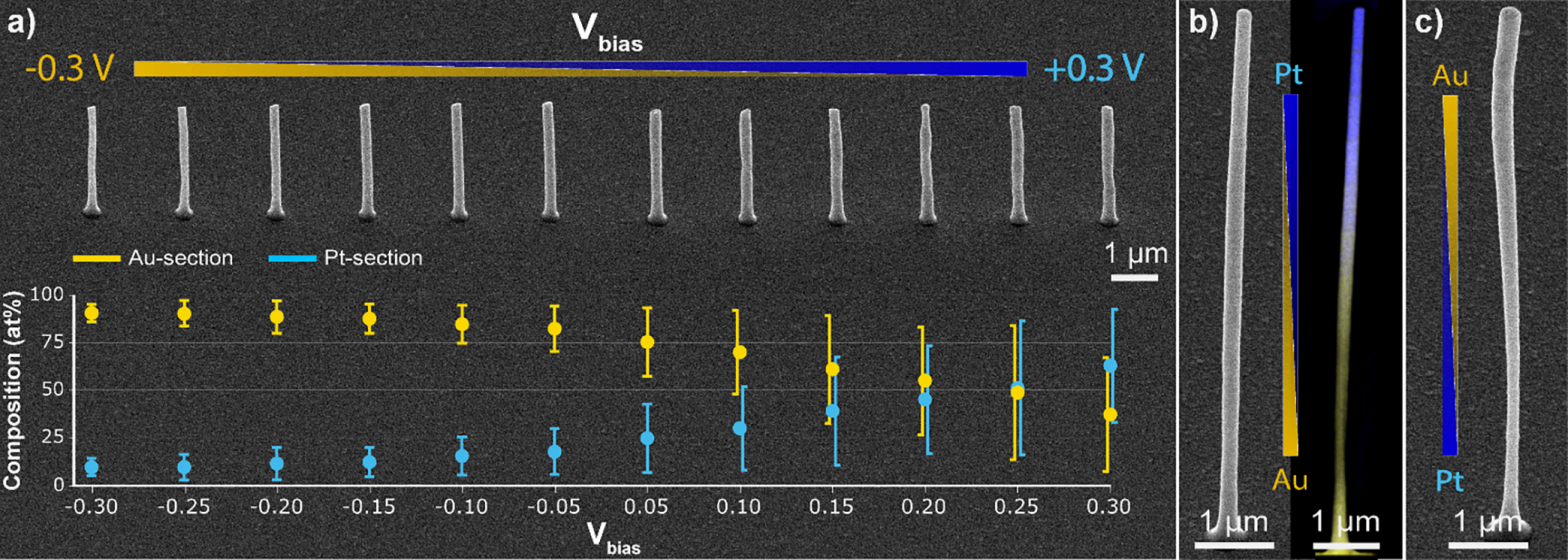
Variable material composition of the printed pillars. **a)** SEM image of a 12-pillar array that illustrates the change of material composition for Au–Pt alloy depending on the applied voltage bias from −0.3 to +0.3 V with a step of 50 mV (skipping 0 V value). The graph under the SEM image shows the corresponding metal content of three printed arrays using independent nozzles. **b, c)** SEM images and EDX map inset (in b) of Au–Pt pillars printed with a gradient in metal composition in vertical direction. The gradient is composed of 30 voxels with 270 nm heights, where the voltage bias changed gradually from −0.3 V (+0.3 V) for the bottom voxel to +0.3 V (−0.3 V) for the topmost voxel for pillars shown in (b) and (c), respectively.

As these results indicate the possibility to steadily change the composition of the metal structure by adjusting the meniscus composition via *V*_*bias*_, it also becomes feasible to achieve an almost seamless composition gradient within a single structure. To demonstrate this, we fabricated two pillars, from Au-rich to Pt-rich composition from top to bottom of the pillar and vice versa. For smooth transition, the pillars were subdivided into 270 nm-tall segments printed with *V*_*bias*_ steps of 25 mV (Figure 3b). The pillar with a Au-rich content at the bottom obtained a consistent diameter without distinctive geometrical features (Figure 3b, left). EDX analysis (inset in Figure 3b) evidences a gradual linear transition in metal content along the pillar length, with up to 91 at% for Au and 37 at% for Pt (). Conversely, a pillar with inverted gradient showed a slimmer base with increasing diameter as the concentration transitioned to Au-rich alloy. Although the reason for this is not entirely clear, we attribute this behavior to experimental imperfections during the printing process. Looking at the data of Au-rich and Pt-rich pillars in Figure 3a, the pillar diameters are slightly different but close enough to indicate that material-specific effects are unlikely to cause the observed shape.

### Morphology and Microstructure of the Printed Alloy

Important morphological and microstructural features relevant to the multimaterial iMCED process are identifiable from the 8-segment “zebra-striped” Au–Pt pillar shown in Figure 4. The corresponding EDX and scanning transmission electron microscopy (STEM) images, which directly relate the chemical content with the morphology, are shown in Figure 4a. The STEM image also features a stark contrast between Au- and Pt-rich sections. Morphology of the Au segment shows clear distinction between the printed individual iMCED layers (transverse to the pillar axis), with Au layers themselves being relatively featureless and smooth. On the contrary, Pt segments appear more “sandy” with a visually rougher surface. The individual layers on Pt, not visible in Figure 4a, could be identified at a higher magnification with high-resolution transmission electron microscopy (Figure 4b). The thickness of these individual layers (11 nm for Pt and 32 nm for Au) is in good agreement with the data extracted from z-piezo data recorded during the printing (Figure 2e) and with clearly visible transition between the morphology of Au and Pt. The latter also confirms a very sharp change in chemical composition upon bias switching, which seems to occur within a single deposited layer of iMCED (). Between the sections, no delamination is observed ().

**Figure 4:**
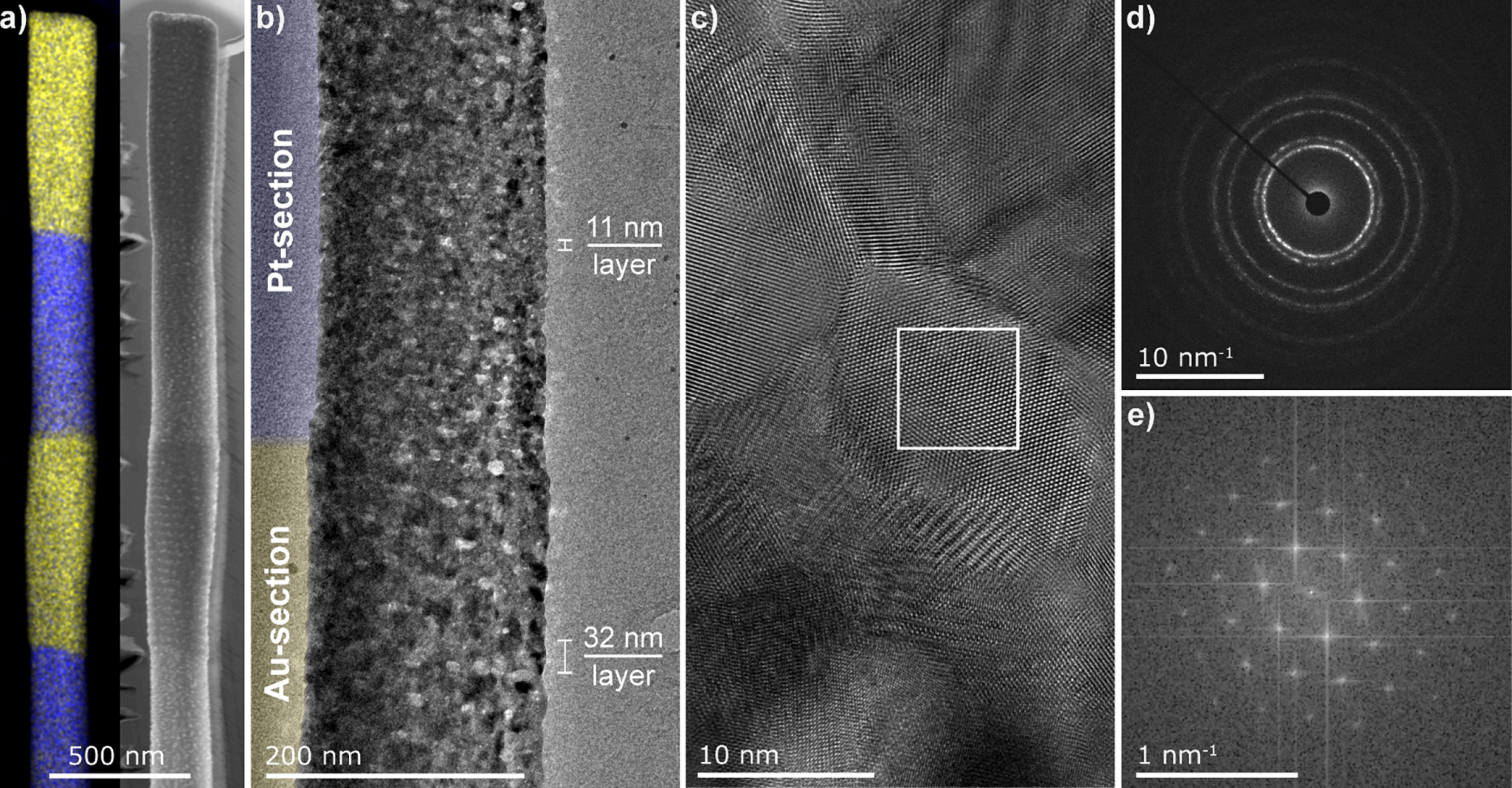
Morphology and microstructure analysis. **a)** EDX map (left) and a corresponding STEM image (right) of a part of an 8-segment Au–Pt pillar. **b)** STEM image showing a close-up view of a border between Au-rich and Pt-rich segments. **c)** HR-TEM image of a Pt-rich segment. **d)** Selected area diffraction pattern of a Pt-rich pillar segment. **e)** FFT image of an area in (c) marked with a white square.

The microstructural features of the Au–Pt alloy are visible from the high-resolution transmission electron microscopy (HR-TEM) and electron diffraction data shown in Figure 4c-e. The printed material is a polycrystalline alloy with individual crystallites of ca. 10–20 nm. No voids are observed in the metal structure, allowing to conclude a non-porous nature of the printed material (). Rings present in the diffraction pattern (Figure 4d) recorded on the selected area of the pillar also confirm the polycrystallinity of the sample.

Interestingly, the diffraction pattern of the Pt-rich section also confirms formation of a single-phase Au–Pt alloy, despite the phase diagram of the Au–Pt system suggests a large miscibility gap that extends for almost all range of possible metal ratios^44^ (). This occurs despite very close lattice parameters of pure metals (4.0780 Å for Au and 3.9231 Å for Pt),^45^ both packed in a face centered cubic (fcc) crystal structure. The existence of the single phase Au–Pt alloy is derived from the dimensions of the smallest ring on the diffraction pattern (4.312 nm^–1^ in the inverse space), which correlates to a *d*-spacing of 2.319 Å. Assuming Vegard’s law (and using a calibration coefficient of 0.9968 accounting for optical aberrations in TEM analysis) this corresponds to the 61:39 ratio of the atomic composition of the Au–Pt for 111 plane, since *d*-spacing for pure metals is 2.356 Å for Au and 2.265 Å for Pt. This is in excellent agreement with EDX data, that suggests 61:39 alloy composition, hence supporting the existence of a solid solution, likely due to the non-equilibrium, kinetics-driven formation of the metallic phases. The diffractograms (Figure 4e) obtained via fast Fourier transform from the selected regions, such as the one marked with a square on Figure 4c, also confirm these conclusions. The closest frequency intensity peaks obtained a *d*-spacing of 2.321 Å and correspond to a composition of Au:Pt (64:36), consistent with previous analysis indicating a single-phase solid solution without phase separation. For Au-rich alloys this does not seem to be the case. The diffraction pattern suggests that the smallest ring corresponds to *d*-spacing of 2.352 Å, which would be equivalent to a 97:3 Au–Pt ratio. EDX data, however, evidence the presence of up to 15 at% of Pt in this segment, suggesting that a significant amount of Pt does not form a 111 phase. These microstructural features of the alloy remain stable for a period of at least 6 months at room temperature ().

The formation of a single fcc phase in the printed Au–Pt pillars can be understood in terms of rapid, far-from-equilibrium growth kinetics during electrodeposition and nanoscale structural confinement. During iMCED, atoms are incorporated into the growing lattice within ca. 11 ms per layer using large overpotentials that drive reduction much faster than atomic rearrangement can occur. Under such conditions, atoms are effectively quenched into a metastable configuration before they can segregate into the equilibrium two-phase mixture, resulting in a kinetically trapped solid solution. The resulting alloy is nanocrystalline (10–20 nm grains) and fully dense (non-porous), as confirmed by HR-TEM. Given this small grain size, long-range diffusion or phase segregation would require atomic migration across multiple grain boundaries. However, the diffusivity of Au in Pt is extremely low (around 1.5 · 10^–23^ cm^2^ s^–1^ at 600 K) and decreases exponentially with temperature (following Arrhenius scaling), becoming effectively negligible at room temperature.^46,47^ Even at 900 K the expected diffusion lengths over minutes are only a few nanometers, insufficient to drive long-range partitioning or spinodal decomposition across multiple grains ().

The nanocrystalline grain structure contributes not only to the suppression of diffusion but also to the stabilization of the nonequilibrium single-phase state by increasing the interfacial energy. Consequently, phase segregation is kinetically inaccessible within the experimental time scales, and the alloy remains metastable yet structurally stable (). Similar behavior has been reported in other electrodeposited alloy systems,^48,49^ where rapid deposition kinetics and nanoscale grain architectures set the conditions for single-phase formation under far-from-equilibrium conditions.

## Conclusions

Meniscus-confined electrodeposition with a multibarrel nozzle is demonstrated as an approach for multimaterial additive manufacturing with precisely controlled composition. The flexibility of this approach is underpinned by the possibility of tuning the ionic concentrations inside the meniscus by applying a bias voltage between the nozzle barrels, which induces an ion current that populates the meniscus with a certain ratio of ionic species, suitable for cationic and anionic metal precursors (as well as their mixtures). Since the voltage bias could be adjusted at a sub-millisecond time scale, and a single iMCED layer is typically printed within 11 ms, this approach allows rapid changes in the composition of the resulting features, hence providing a tool to create multimaterial interfaces with sub-voxel heights (in our case, 125 nm thick segments for voxels with ca. 200–250 nm lateral dimensions), as well as high-aspect ratio out-of-plane structures with smooth material composition gradients. Material analysis of the fabricated alloys suggests that metals could be deposited with a wide window of chemical compositions, with Au–Pt and Cu–Au alloys of ca. 60 to 96 at% and 30 to 52 at% of Au content, respectively. As confirmed by STEM and HR-TEM, Au–Pt alloys are fully dense (non-porous) polycrystalline materials, featuring 10–20 nm grains, with the evidence of the existence of Au–Pt solid solution. While the present work focuses on noble metal systems, we anticipate that the demonstrated approach can be extended to other electrochemically depositable metals. Such extensions, however, require optimization and validation for each electrolyte configuration as meniscus-confined deposition conditions are highly system-specific. With the possibility to vary chemical composition of the metal structures, multibarrel MCED opens exciting opportunities for creating new materials and interfaces for future nanoelectronics, sensing, and catalytic interfaces, suitable for a wide range of state-of-the-art applications. The demonstrated capability for rapid bimetallic switching and nanoscale 3D structuring positions this platform as a promising tool for next-generation CO_2_-reduction electrocatalyst fabrication. Cu–Au systems, in particular, are known to benefit from synergistic pathways, where Au promotes CO formation and Cu converts CO into C_2+_ products.^50–54^ Nanoscale engineering of the multimetal sections from both metals could facilitate CO spillover and enhance C–C coupling,^54–56^ thus overcoming the current fabrication limitations in the resolution and compositional flexibility needed to systematically produce such architectures.^57^ The proposed iMCED method offers nanoscale voxel-level compositional control and precise 3D structuring, which can become a platform to accelerate catalyst discovery and optimization.^58^

## Methods

### Chemicals

All chemicals were used as received. Deionized (DI) water produced by a Millipore Milli-Q Direct 8/16 system with a resistivity of 18.2 MΩ cm (25 °C) was used to prepare aqueous solutions. Electrolyte solutions for the Cu–Au system were prepared using HAuCl_4_·3H_2_O (trace metal basis, > 99.9%, Sigma-Aldrich, Switzerland), CuSO_4_·5H_2_O (trace metal basis, > 99.9%, Sigma-Aldrich, Switzerland), and H_2_SO_4_ (1 M, Carl Roth, Germany), while the ones for the Au–Pt system were prepared using HAuCl_4_·3H_2_O (30 wt% in diluted HCl, trace metal basis, > 99.9%, Sigma-Aldrich, Germany), and H_2_PtCl_6_ (20 wt%, Thermo Fisher Scientific, Germany). All Au and Pt solutions were freshly prepared and handled under minimal light exposure.

### Nanopipette Probes

Nanopipettes were pulled from borosilicate theta capillaries (Harvard apparatus #30–0114, 1.5 mm OD, 0.23 mm Wall, 0.17 mm Septum, 100 mm long) using a laser pipette puller (P-2000, Sutter Instruments). Single barrel nanopipettes were pulled from borosilicate capillaries (Harvard apparatus #30–0044, 1.2 mm OD, 0.255 mm Wall, 100 mm long) using the same laser pipette puller.

### MCED Setup

Nanopipette probes, mounted on a custom-made probe holder, were coarsely positioned over a sample with a mechanical XY micropositioner and vertical step motor (MMP1, MadCityLabs, USA). The substrate was mounted on the integrated micro-nanopositioning system (Nano-View/M 100–2) with a XY axis step motor micropositioners and a XYZ piezo-positioner (Nano-MET10, MadCityLabs, USA). The setup was built on top of a vibration isolation platform MinusK BM-8, and it was surrounded by a faraday cage. Electrical measurements were performed using a high-bandwidth and low-noise current amplifier (DLPCA-200, Femto, Germany). The setup was controlled through an FPGA card (PCIe-7858R, National Instruments) using a home-written program in a LabVIEW interface, based on the WEC-SPM software package (kindly provided by Prof. Patrick Unwin, University of Warwick), that was used for data acquisition.

### Substrates

All printing experiments were conducted using gold-coated glass substrates (50 nm Au, Micro- to Nano, Netherlands). Substrates were cleaned by 10 min ultrasonication in DI-water, followed by 10 min in isopropanol and rinsed with DI-water, followed by O_2_-plasma treatment (13.56 MHz, Zepto, Diener, Germany) for 10 min prior to use.

### Characterization

Electron microscopy characterization of nanopipette probes was performed with a transmission electron microscope JEOL JEM-2100F at 200 kV accelerating voltage and 122 μA emission current. Structure characterization for angled SEM and EDX mapping was performed using Hitachi SU 5000 operating at 15 kV with an EDX detector from Oxford Instruments. TEM lamellae of the pillars were prepared by FIB on a Thermo Fisher Scientific (TFS) Helios 5 UX. Further STEM imaging and EDX mapping were performed using a TFS Talos F200X at 200 kV with SuperX EDS. High-resolution TEM images were acquired on a JEOL GrandARM operating at 300 kV.

## Supplementary Material





## Notes

Details on preparation of the nozzles, finite element method simulations, EDX analysis of pillars, segment height comparison, compositions achieved with a single barrel printing with mixed electrolyte, EDX analysis of gradient pillar, high-resolution microstructure imaging and analysis, selected area electron diffraction analysis (PDF)
COMSOL report file (PDF)
