# Supplementary material for: Additive Manufacturing of Nanoscale Multimaterial Voxels Via Meniscus-Confined Electrodeposition
Source: ACS Nano. 2026 Apr 8;20(15):11604–13. doi: 10.1021/acsnano.5c16931 (PMC13104162; doi:10.1021/acsnano.5c16931)
Supplement: Supplementary file 1 [file nn5c16931_si_001.pdf]

# Additive Manufacturing of Nanoscale Multi-Material Voxels via Meniscus-Confined Electrodeposition

# Supporting Information

*Simon Sprengel,[1,†] Julian Hengsteler,[2,†] Peng Zeng,[3] Albert Ripoll Oliveras,[2] Muhammad Zerehi Zadeh,[1] Weishan Wu,[1] Andrei Zotov,[2] Daniel Torres,[4] Xinhua Zhu,[4, 5] Jon Ustarroz,[4 ,5] Vita Solovyeva,[6] Tomaso Zambelli,[2] and Dmitry Momotenko[1,]**

[1] *Laboratory of Electrochemical Nanotechnology, Institute of Chemistry, Carl von Ossietzky Universität Oldenburg, Oldenburg D-26129, Germany*

[2] *Laboratory of Biosensors and Bioelectronics, Institute for Biomedical Engineering, ETH Zürich, Zurich CH-8092, Switzerland*

[3] *ETH Zürich, The Scientific Center for Optical and Electron Microscopy (ScopeM), 8093 Zurich, Switzerland*

[4] *Chemistry of Surfaces, Interfaces and Nanomaterials (ChemSIN), Université libre de Bruxelles, 1050 Brussels, Belgium; Electrochemical and Surface Engineering (SURF), Vrije Universiteit Brussel, 1050 Brussels, Belgium*

[5] *Electrochemical and Surface Engineering (SURF), Vrije Universiteit Brussel, 1050 Brussels, Belgium*

[6] *Carl von Ossietzky Universität Oldenburg, Fakultät V, Institut für Physik, Oldenburg 26129, Germany*

*dmitry.momotenko@uol.de

[†]*authors contributed equally*

## SI-1. Preparation of theta pipette nozzles

Theta nozzles were prepared using a $CO_2$-based Sutter Instruments P-2000 laser puller with borosilicate theta capillaries (Harvard apparatus #30-0114 borosilicate glass, 1.5 mm OD, 0.23 mm Wall, 0.17 mm Septum, 100 mm long). Single barrel capillaries of same manufacturer had dimensions of 1.2 mm OD, 0.255 mm Wall, 100 mm long (Harvard apparatus #30-0044). Nanopipettes with total opening diameter of about 490 nm for double-barrel and about 800 nm for single-barrel nozzles were produced using the following programs:

**Table SI-1:** Pulling parameters for printing nozzle using Sutter Instrument P-2000 pipette puller featuring double-barrel theta and single barrel pipette fabrication.

| Pipette opening diameter | Line No. | Heat | Filament | Velocity | Delay | Pull |
|---|---|---|---|---|---|---|
| 490 nm, double barrel | 1 | 600 | 4 | 30 | 150 | 20 |
| | 2 | 500 | 4 | 30 | 150 | 60 |
| | 3 | 500 | 3 | 30 | 135 | 60 |
| 800 nm, single barrel | 1 | 350 | 3 | 30 | 220 | 0 |
| | 2 | 350 | 3 | 50 | 180 | 40 |

Nozzle dimensions were confirmed by SEM, suggesting the following nozzle parameters for the outer (OD = 492 ± 30 nm; $CI_{95}$, n = 13) and inner diameters (ID = 311 ± 23 nm; $CI_{95}$, n = 13).

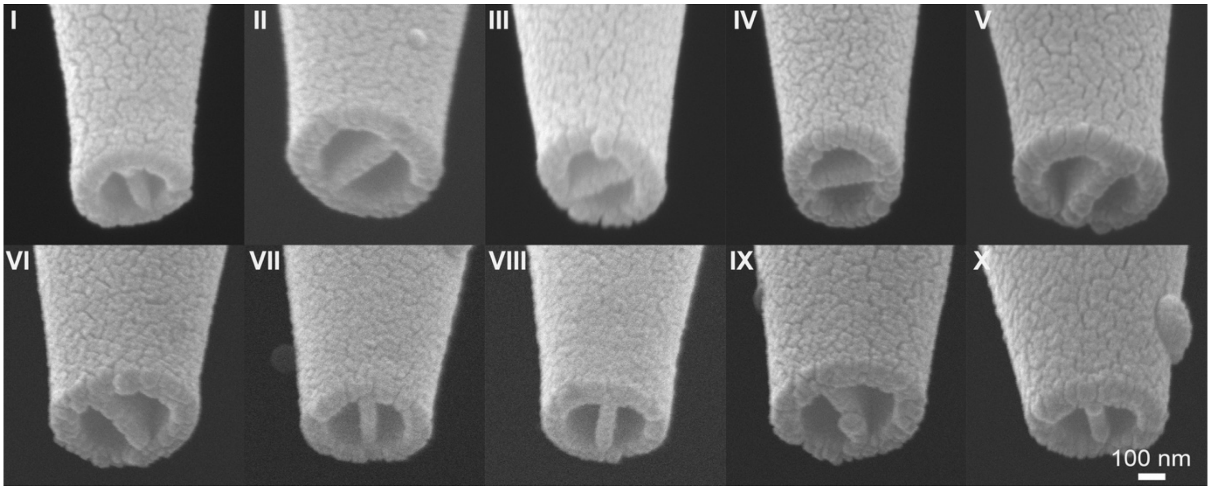


**Figure SI-1:** SEM images of double-barrel nanopipette nozzles.

## SI-2. Finite element simulations

Finite element simulations were performed using *COMSOL Multiphysics* (Version 6.2.0.658, win64) on Intel® Core™ i9-10850K CPU @ 3.60GHz equipped with 128GB RAM. The exact model description is provided in form of COMSOL Report, provided as a separate file.

### Geometry and Mesh

The finite element model was constructed as a three-dimensional representation of the theta-nanopipette and the liquid meniscus bridging the substrate. 3D geometry simulations are required to accurately model the experiment configuration since two-dimensional axisymmetric models cannot adequately capture the geometry that includes a non-axisymmetric double-barrel nanopipette probe. To reduce computational efforts while preserving accuracy, the model was cut along the longitudinal axis, simulating one half of the cross-section (Figure SI-2a–c).

The pipette geometry was adapted from high-resolution TEM image of the theta nozzle (Figure SI-2a), only slightly smaller than mean estimated from SEM (420 vs 490 nm OD). TEM allowed to capture exact geometric details of the inner and outer geometry of the nozzle. Based on these experimental dimensions, two individual barrels separated by a septum with the thickness $w_{th}$ combined by a hemispherical liquid meniscus of the height equal to $h_M$. The septum was simulated with fillets of the same radius as the septum thickness in order to avoid sharp corners and discontinuities.

Meshing of the computational domain was performed using tetrahedra (in total ca. 2.4 million elements). Particular care was taken to ensure fine mesh density in regions of high expected gradients (i) at the barrel openings being concentration boundaries to the meniscus, and (ii) at the substrate boundary, where deposition occurs and where the flux is calculated. Similarly, the barrel

openings were refined with high mesh density to resolve the sharp gradients in electric potential and ion concentrations (Figure SI-2c). The remaining bulk regions were meshed more coarsely to minimize computational efforts.

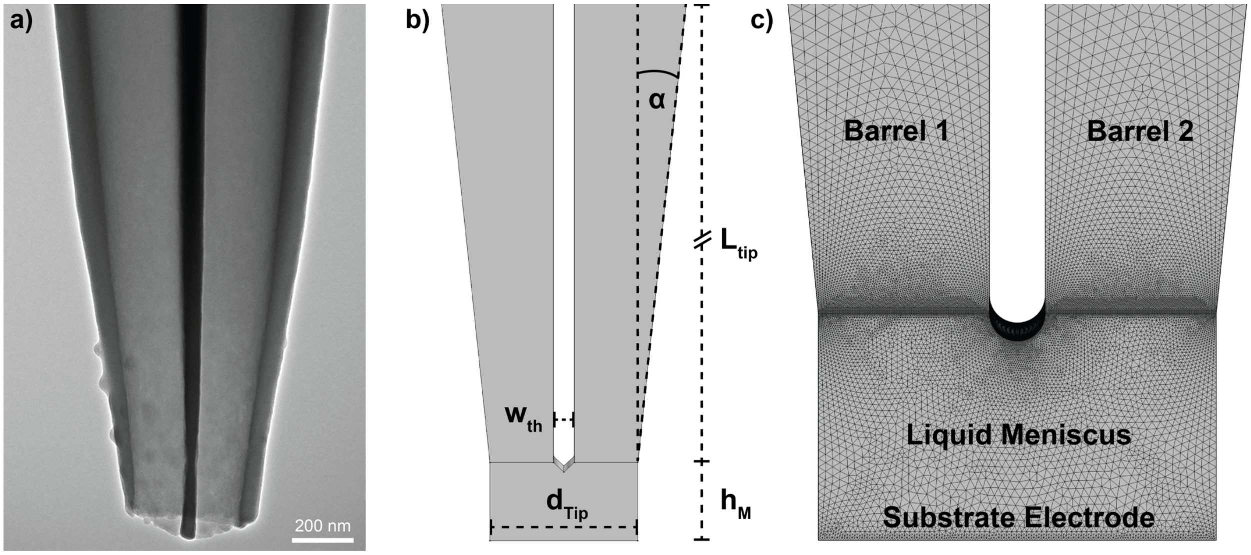


**Figure SI-2:** Geometry and mesh of the FEM model. (a) TEM image of a theta nanopipette tip used as reference for the computational geometry. (b) Adapted 3D geometry in COMSOL showing the dual barrels, septum, and liquid meniscus bridging the substrate electrode. (c) Close-up of the pipette opening and meniscus with the applied mesh, refined at the barrel exits and substrate boundary.

Important model parameters are presented in Figure SI-2b, including tip opening diameter $d_{Tip}$, wall thickness of the septum $w_{th}$, conical angle of the pipette $\alpha$, total length of the pipette $L_{Tip}$, and height of the liquid meniscus $h_M$. The latter according to our data is 190 ± 14 nm for Au and 172 ± 9 nm for Pt. Averaging both values yields an effective meniscus height of approximately 181 nm for the process. For simplicity, we assumed a meniscus height of half total diameter (180 nm) of the nozzle. All numerical values are supplied in Table SI-2:

**Table SI-2:** Numerical values assigned to the simulation parameters. Expressions and constants are defined as used in the finite element simulations of the theta pipette.

| Name | Expression | Value | Description |
|---|---|---|---|
| $L_{tip}$ | 20 [µm] | $2.0 \cdot 10^{-5}$ m | total length of tip |
| $\alpha$ | 5.6 [°] | 0.097738 rad | cone semi angle |
| $r_{Tip,open}$ | 160 [nm] | $1.6 \cdot 10^{-7}$ m | total radius of tip opening |
| $w_{th}$ | 45 [nm] | $4.5 \cdot 10^{-8}$ m | wall thickness septum |
| $d_{Tip}$ | $2r_{Tip,open} + w_{th}$ | $3.6 \cdot 10^{-7}$ m | total diameter of tip opening |
| $r_{Tip,top}$ | $r_{Tip,open} + L \cdot \tan(\alpha)$ | $2.1 \cdot 10^{-6}$ m | total radius of tip opening |
| $h_M$ | $\frac{d_{Tip}}{2}$ | $1.8 \cdot 10^{-7}$ m | height of liquid meniscus |
| $c_{0,Au}$ | 10 [mmol/L] | 10 mol/m³ | concentration of Au |
| $c_{0,Pt}$ | 110 [mmol/L] | 110 mol/m³ | concentration of Pt |
| $c_{0,Cu}$ | 10 [mmol/L] | 10 mol/m³ | concentration of Cu |
| $c_{0,H2SO4,bulk}$ | 500 [mmol/L] | 500 mol/m³ | concentration of bulk $H_2SO_4$ |
| $c_{0,H2SO4,b2}$ | 507.6 [mmol/L] | 507.6 mol/m³ | concentration of $H_2SO_4$ in barrel 2 |
| $D_{Au}$ | $D_{Au} = D_{Pt}$ | $1.2 \cdot 10^{-9}$ m²/s | diffusion coefficient $AuCl_4^-$ |
| $D_{Pt}$ | | $1.2 \cdot 10^{-9}$ m²/s | diffusion coefficient[1] $PtCl_6^{2-}$ |
| $D_{Cu}$ | | $0.714 \cdot 10^{-9}$ m²/s | diffusion coefficient[2] $Cu^{2+}$ |
| $D_H$ | | $9.311 \cdot 10^{-9}$ m²/s | diffusion coefficient[2] $H^+$ |
| $D_{SO4}$ | | $1.065 \cdot 10^{-9}$ m²/s | diffusion coefficient[2] $SO_4^{2-}$ |
| $u_{Au}$ | $\frac{1FD_{Au}}{RT}$ | $4.6731 \cdot 10^{-8}$ m²/s | mobility of $AuCl_4^-$ ions |
| $u_{Pt}$ | $\frac{2FD_{Pt}}{RT}$ | $9.3462 \cdot 10^{-8}$ m²/s | mobility of $PtCl_6^{2-}$ ions |
| $u_H$ | $\frac{1FD_H}{RT}$ | $3.6255 \cdot 10^{-7}$ m²/s | mobility of $H^+$ ions |
| $u_{Cu}$ | $\frac{2FD_{Cu}}{RT}$ | $5.5610 \cdot 10^{-8}$ m²/s | mobility of $Cu^{2+}$ ions |
| $u_{SO4}$ | $\frac{2FD_{SO4}}{RT}$ | $8.2948 \cdot 10^{-8}$ m²/s | mobility of $SO_4^{2-}$ ions |
| $V_{b1,Au}$ | | -1.05 V | Au-Pt potential right barrel 1 |
| $V_{b2,Pt}$ | | -0.75 V | Au-Pt potential left barrel 2 |
| $V_{b1,Cu\text{-}Au}$ | | -1.05 V | Cu-Au potential right barrel 1 |
| $V_{b2,empty}$ | | -0.75 V | Cu-Au potential left barrel 2 |
| $T$ | | 298.15 K | temperature |
| $R$ | | 8.31 J/(mol K) | gas constant |

| Name | Expression | Value | Description |
|---|---|---|---|
| $F$ | | 96485 C/mol | Faraday constant |

**Ion transport (Nernst-Planck model):**

The transport of metal ion precursor species in the double barrel and meniscus was described using the Nernst-Planck equation in the absence of fluid flow, resolved in a steady-state formulation:

$$\nabla \cdot \left(-D_i \nabla c_i - \frac{z_i F}{RT} D_i c_i \nabla V\right) = 0 \qquad \textbf{Eq SI-1}$$

where $D_i$, $c_i$, $z_i$, $V$ denote diffusion coefficient, concentration, ion charge number, and electric potential respectively.

The consumption of the species at the substrate was simulated as a concentration boundary condition, setting it to 0 for all electroactive species. This assumes infinitely fast kinetics of the electrodeposition process.

**Electric fields and current simulation**

To simulate the distribution of electric potential, the model solved the following relation

$$\nabla(\sigma E) = 0 \qquad \textbf{Eq SI-2}$$

Conductivity σ of the electrolyte was defined as

$$\sigma = F \cdot \sum_i z_i^2 c_i \frac{F D_i}{RT} \qquad \textbf{Eq SI-3}$$

thus, taking into account all ionic species.

## Model validation

In order to ensure that the model correctly describes physicochemical phenomena, the results of numerical simulations were compared to the values available from analytical approximation.

First, we validated the simulation of the species flux upon electrodeposition on the substrate using only diffusional mass-transport. The diffusion cases towards an ultra-microelectrode are illustrated with and without pipette restricted diffusion in **Error! Reference source not found.**Figure SI-3.

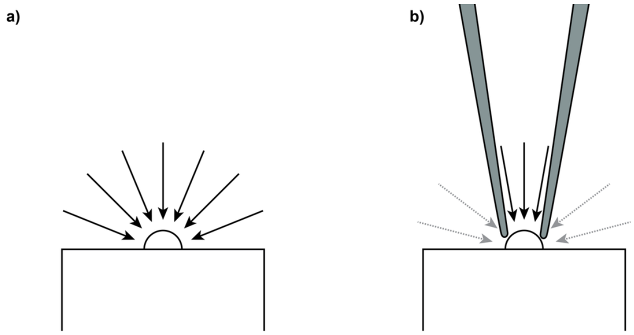


**Figure SI-3:** (a) Schematic of a hemispherical diffusion towards an ultra-microelectrode (UME) and (b) in a nanopipette configuration, where the diffusional transport is limited by a conical geometry.

The analytical expression for this model is based on approximation similar to a hemispherical diffusion equation, with the exception that for a nanopipette the diffusion is limited by its conical walls. In general, the spherical diffusion equation reads

$$I_d = \Omega\, n\, F\, D\, c\, r_0 \qquad \textbf{Eq SI-4}$$

where $I_d$, $n$, $r_0$ denote diffusion limited current, number of electrons transferred per molecule, and radius of spherical electrode respectively. $\Omega$ is a solid angle, defined as

$$\Omega = 2\pi[1 - cos(\alpha)] \qquad \textbf{Eq SI-5}$$

For a sphere the solid angle is 4π, for a hemisphere it turns to 2π and in the case of a nanopipette the equation becomes

$$I_d = 2\pi[1 - cos(\alpha)]n\; F\; D\; c\; r_0 \qquad \textbf{Eq SI-6}$$

with

$$r_0 = \frac{r_{Tip,open}}{\tan(\alpha)} \qquad \textbf{Eq SI-7}$$

We compared the value of a steady-state current calculated using this expression with the numerical value for electrodeposition of Au from 10 mM solution. The values of the electrodeposition current (170 pA vs 149 pA for analytical and numerical models, respectively) are different by ca. 12%. Given that the analytical solution is only approximation that does not take into account additional geometrical features of the nanopipette (meniscus height, presence of the septum), the values are in a relatively good agreement.

Second, we checked the combined electrical and mass-transport model against an analytical approximation. In this case, we used an equation that estimates inner resistance of the nanopipette as described elsewhere.[3] For a simulation, where the $HAuCl_4$ is present in both barrels at equal concentration, assuming no Au deposition at the substrate and at 0.1 V of applied bias, the conductance current through the tip approaches a value of 453.75 pA, which is only 6.9% different from the result of analytical expression (482.12 pA)

$$I_M = V_{bias} \cdot \frac{1}{2}\left[4\pi\, r_{Tip,open}\, \sigma \left(\frac{sin^2\left(\frac{\alpha}{4}\right)}{sin\left(\frac{\alpha}{2}\right)}\right)\right] \qquad \textbf{Eq SI-8}$$

Here, the factor 1/2 appears since the analytical solution here (vs original equation) takes into account ion transport through two barrels (vs one for the original equation formulation), while α/2

and $\alpha/4$ are used instead of $\alpha$ and $\alpha/2$ because the semi-angle of a single compartment in the barrel is halved.

**Comparison of substrate potentials $E_{sub}$ at various bias magnitudes for Cu-Au and Au-Pt**

As mentioned, in a double-barrel setup the conductivity of each barrel influences the $E_{sub}$ and therefore the electrodeposition. Due to the presence of two QRCEs in the pipette, the $E_{sub}$ has to be referenced to both applied potentials respectively following this equation:

$$E_{sub} = -(\alpha_v V_1 + (1 - \alpha_v) V_2) \qquad \textbf{Eq SI-9}$$

*$\alpha_v$: coefficient of symmetry between barrels; $V_1$: applied potential barrel 1; $V_2$: applied potential barrel 2*

In case of ideal symmetry and matched conductivity between the barrels, the influence of both barrels on the $E_{sub}$ is equal, with $\alpha_v$ of 0.5:

$$E_{sub} = -\frac{V_1 + V_2}{2} \qquad \textbf{Eq SI-10}$$

To determine the $E_{sub}$ for both the Cu-Au and Au-Pt systems, the average $E_{sub}$ was calculated in the center of the substrate electrode boundary for a bias range of -0.2 to +0.2 V with an increment step size of 10 mV. For the simulation, the barrel voltages were set as $V_2 = -V_1$. The obtained simulation data of both systems is visualized in Figure SI-4. For the Cu-Au system with matched barrel conductivity, practically no deviation from the expected $E_{sub}$ of 0 V is observable. From this data, $\alpha_v$ can be obtained from linear regression of the simulated $E_{sub}$ at different $V_1$, where the slope of the linear relationship between $E_{sub}$ and $V_1$ is (1-2$\alpha_v$), which is clear when substituting $V_2$ with -$V_1$ in the equation Eq SI-9.:

$$E_{sub} = -(\alpha_v V_1 + (1-\alpha_v)V_2) = -(\alpha_v V_1 - (1-\alpha_v)V_1) = (1-2\,\alpha_v)V_1 \qquad \textbf{Eq SI-11}$$

The Cu–Au system matches a symmetrical case with $\alpha_v$ of 0.500. Comparing those results to the Au-Pt system, barrel of higher conductivity (barrel 2, Pt-species) dominates the substrate potential. This is also indicated by $\alpha_v$ value of 0.201, obtained by applying Eq SI-11. This allows further estimation of the substrate voltages depending on the chosen $V_1$ and $V_2$ for Au-Pt system.

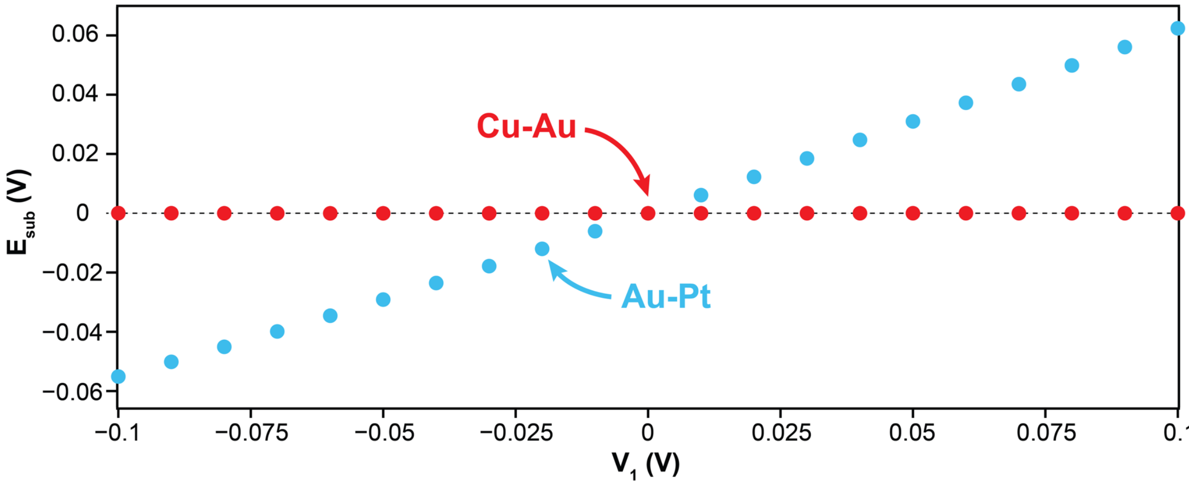


**Figure SI-4:** Simulated substrate potentials $E_{sub}$ at various barrel 1 voltages $V_1$ for the Cu-Au (red) and Au-Pt (blue) system.

## <u>Metal species fluxes at electrode substrate at various bias magnitudes</u>

The composition of deposited features is determined as the ratio of the flux magnitudes for both species towards the substrate electrode. Here, we simulated flux ratios between different metallic species for each system. The Cu-Au system was modeled in a bias range from -0.26 V to +0.26 V using an increment step size of 20 mV. The Au-Pt system was simulated in a slightly narrower range (-0.20 V to +0.20 V with same increment size) due to convergence issues.

The Cu-Au system shows that 50:50 metal ratio should occur at $V_{bias}$ = +0.03 V, slightly shifted to the Cu side (Figure SI-5a). $V_{bias}$ has a strong effect on the composition of the printed alloy, and biases exceeding +/- 0.2 V should lead to almost pure metals. In respect to this, the Au-Pt system shows a similar trend, but the 50:50 metal ratio is offset to lower bias potentials (Figure SI-5b) around $V_{bias}$ = -0.09 V.

These results indicate that $V_{bias}$ has a strong influence on the composition of the printed structures although the experimentally observed metal ratios do not quantitatively match the theoretical predictions.

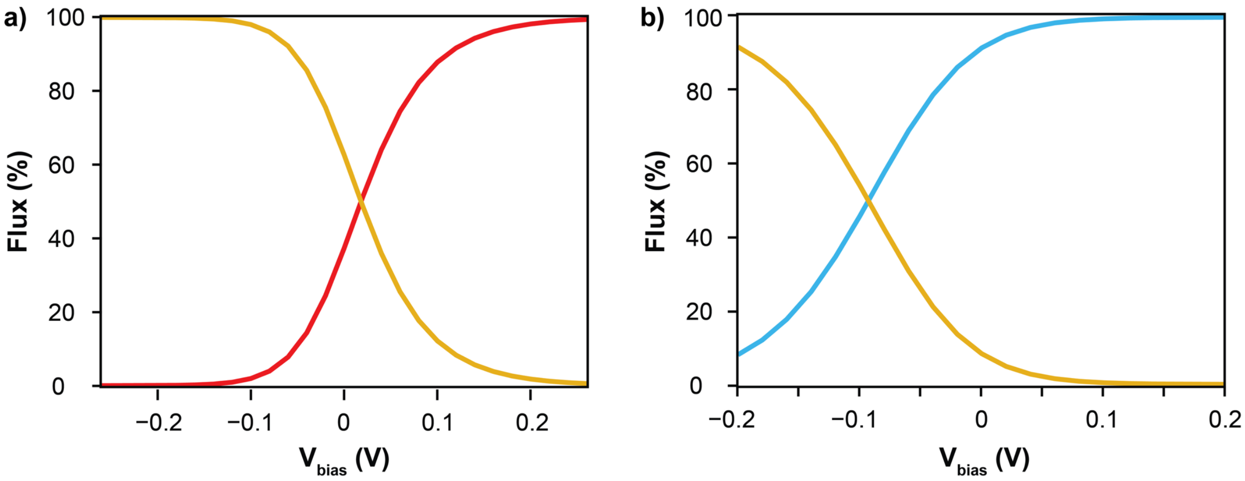


**Figure SI-5:** Flux simulation for a) Au (yellow) and Cu (red) and b) Au (yellow) and Pt (blue) vs $V_{bias}$ in Au-Cu and Au-Pt systems, respectively. The flux is simulated for the named ion species at the substrate electrode boundary without kinetic limitations, hence, total consumption. In the two-metal setup, the sum of both percentual fluxes is equal to the liquid composition at the interface.

As could be expected, a similar trend is observed for the ionic compositions inside the meniscus under the conditions when species are not consumed at the substrate (Figure SI-6). This result highlights that the control of the composition is provided by the bias-driven ionic mass-transport.

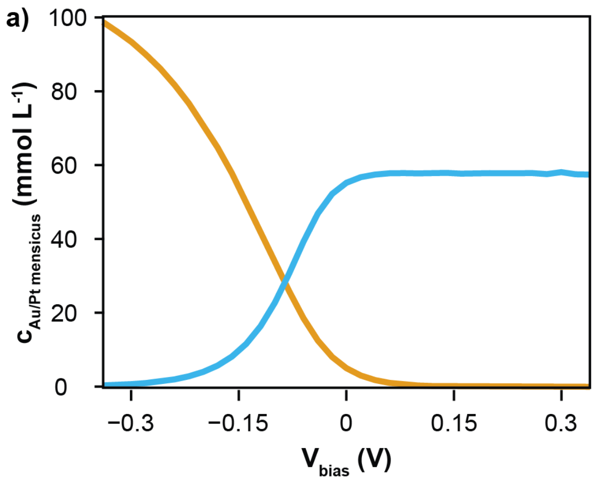


**Figure SI-6:** Simulated ionic concentrations (a) and molar fractions (b) of metal ion precursors (blue – Pt, orange – Au) inside the meniscus at different bias values for Pt-Au system.

## SI-3. EDX analysis of Cu-Au 8-segmented "zebra-striped" alternating pillar

The EDX signal intensities for Cu (red) and Au (yellow) for multi-segment Au-Cu pillar are shown in Figure SI-7. Chemical composition of the alternating Au and Cu sections was determined via high resolution quantitative EDX mapping of the pillar's side profile. Average segment compositions were determined by analyzing spectra traced across the individual sections of the pillar. The overlay plot displays the elemental fractions in at%, with Au indicated in yellow and Cu in red.

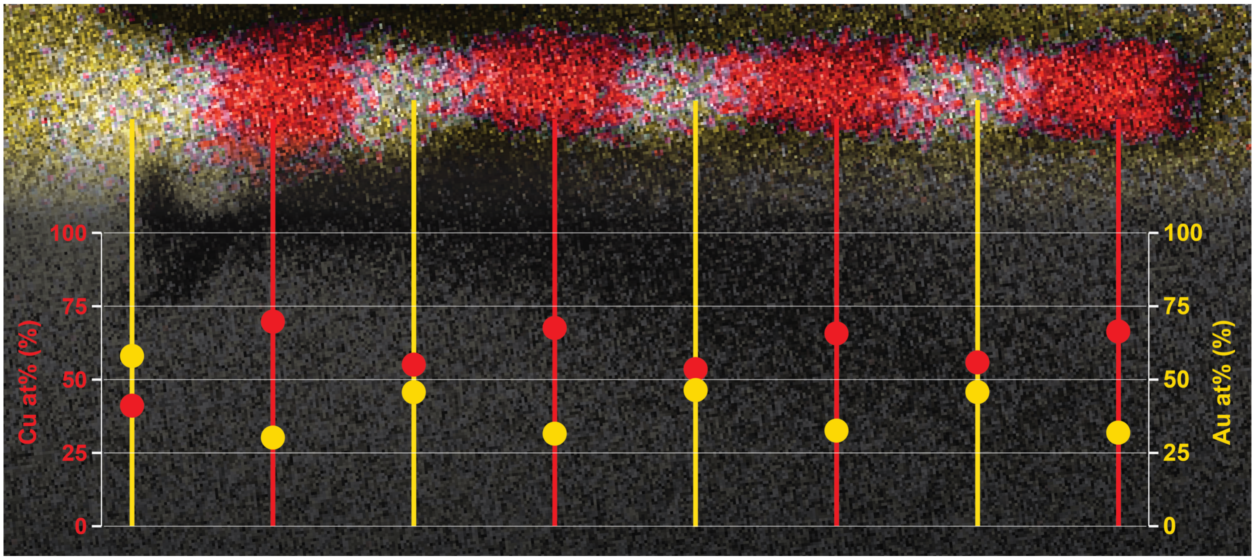


**Figure SI-7:** Quantitative EDX mapping analysis of alternating "zebra-striped" Cu-Au pillar structure. The overlay plot shows the elemental composition (at%) of the 8-segmented Cu-Au pillar, with Cu fractions indicated in red and Au fractions in yellow. Colored guiding lines link the data points to the corresponding "zebra-sections" and reflect the applied deposition biases (yellow: Au at $V_{bias}$ = -0.7 V, red: Cu at $V_{bias}$ = +0.7 V). Au-assigned segments average 49 at% Au and 51 at% Cu, with individual compositions of 54, 53, 54, and 42 at% Cu. Cu-assigned segments average 69 at% Cu and 31 at% Au, with individual segment values of 68, 68, 69, and 71 at% Cu. The periodic modulation of Cu and Au signals confirms the successful alternation of material deposition.

## SI-4. EDX analysis of Pt-Au 8-segmented "zebra-stripe" alternating pillar

Chemical composition of the alternating Au and Pt sections was determined via high resolution quantitative EDX mapping of the pillar's side profile. Average segment compositions were determined by analyzing spectra traced across the individual sections of the pillar. The overlay plot in Figure SI-8 displays the elemental fractions in at%, with Au indicated in yellow and Pt in blue. Connecting guiding lines link the data points to the corresponding "zebra-sections" and use the same color code as the applied deposition biases (yellow: Au at $V_{bias}$ = -0.3 V, blue: Pt at $V_{bias}$ = +0.3 V).

The periodic variation of Au and Pt signals demonstrates successful material alternation throughout the pillar. Segments assigned to Au contain on average 86 at% Au and 14 at% Pt, while

Pt-assigned segments show 61 at% Au and 39 at% Pt. For the individual Au segments (from top to bottom), the measured Au amounts reached 86, 86, 85, and 85 at%. The Pt segments yielded 39, 38, 40, and 46 at% Pt, respectively (the rest is Au).

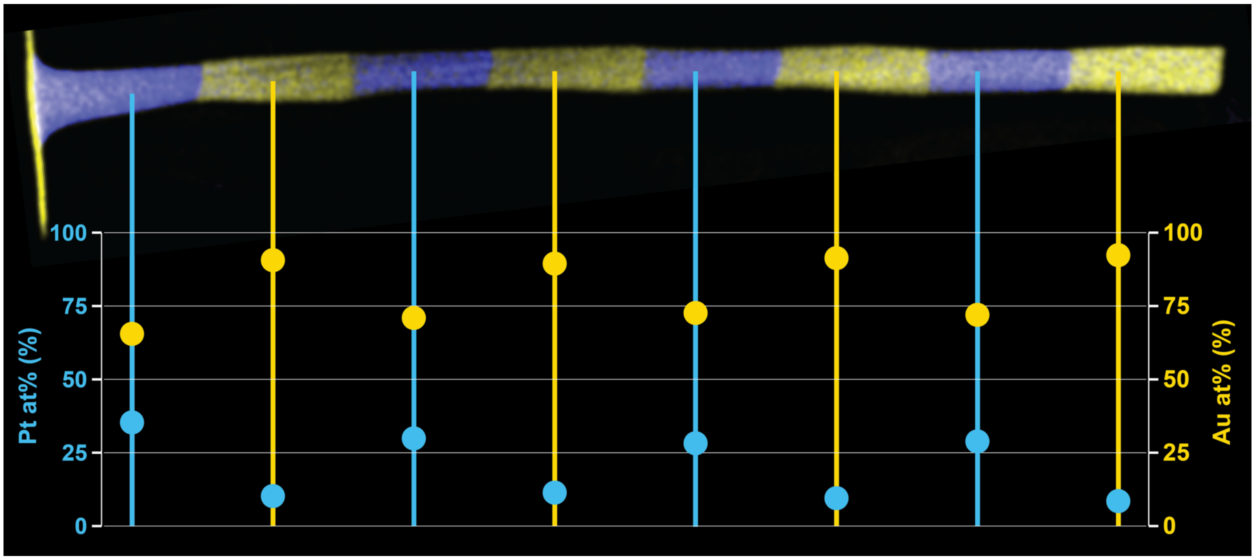


**Figure SI-8:** Quantitative high resolution EDX side profile mapping of 8-segmented "zebra-striped" Au-Pt pillar structure. Yellow and blue dots represent the Au and Pt atomic fractions, respectively, while guiding lines connect the data points to the corresponding "zebra-sections" and reflect the applied deposition biases (yellow: Au at $V_{bias}$ = -0.3 V, blue: Pt at $V_{bias}$ = +0.3 V). The alternating modulation of Au- and Pt-rich regions confirms successful compositional switching, with Au segments averaging 86 at% Au with 14 at% Pt, and Pt segments averaging 39 at% Pt with 61 at% Au.

## SI-5. Segment height comparison of print data, STEM, and EDX analysis

The sharp interfaces observed between adjacent Au and Pt sections suggest rapid switching of meniscus composition during printing. To further investigate the dynamics of this process, we cross-checked the switching events recorded across printer's data (piezo positioner's coordinate) with the segment heights from STEM and EDX measurements. In the recorded data, each change

of material is recorded with its precise z-position from the nanopositioning system acquired at 2 kHz frequency.

Figure SI-9 compares the nominal segment heights from the print data with the corresponding values measured from imaging with STEM (left image) and high-resolution elemental EDX mapping (right image). Across all eight segments, a close agreement is observed between the heights.

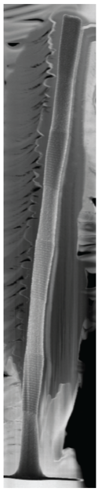

| Segment | STEM | Print Data | EDX |
|---|---|---|---|
| | Segment Height [μm] | | |
| Au | 0.769 | 0.762 | 0.766 |
| Pt | 0.723 | 0.733 | 0.714 |
| Au | 0.738 | 0.740 | 0.752 |
| Pt | 0.697 | 0.711 | 0.691 |
| Au | 0.765 | 0.759 | 0.770 |
| Pt | 0.730 | 0.731 | 0.699 |
| Au | 0.747 | 0.748 | 0.760 |
| Pt | 0.833 | 0.876 | 0.841 |

2 μm

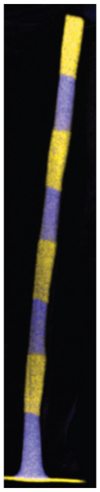

**Figure SI-9:** Comparison between segment heights measured by different methods for the 8-segmented Au-Pt pillar. The table lists the measured heights from the print data alongside values obtained from STEM and EDX. The left image shows the STEM micrograph of the pillar, while the right image displays the corresponding high-resolution EDX elemental map, both used for segment height measurements.

## SI-6. EDX compositions and print speed from mixed electrolyte single barrel printing

To compare the performance of double-barrel e-AM with conventional single-barrel iMCED, experiments were performed using a mixed electrolyte containing 10 mM $HAuCl_4$ and 110 mM $H_2PtCl_6$ with a single barrel pipette. By tuning the substrate potential ($E_{sub}$) between -0.40 V and -0.95 V, it was possible to modulate the relative deposition of Au and Pt within a single nozzle configuration.

Printing at $E_{sub}$ at -0.40 V, -0.45 V, and -0.50 V yielded similar compositions of 97 at% Au. This plateau suggests that below -0.50 V, further lowering of the potential does not significantly increase the Au fraction in the deposit. At more negative potentials, pillars printed at -0.90 V, -0.95 V, and -1.00 V, resulted in 32, 37, and 36 at% Pt, respectively. For comparison, printing a single pillar with double-barrel printing conditions at -0.95 V and Pt-bias resulted in 39 at%.

Strong differences in print speed between mixed-electrolyte single-barrel and double-barrel bias control configurations were observed. To illustrate this, the recorded growth rates of pillars printed at -0.40 V and -0.95 V in a single-barrel configuration were compared to the corresponding Au- and Pt-bias conditions in the dual-channel setup from the print file z-retraction data. For Au-rich compositions, the single-barrel mixed-electrolyte approach yielded a growth rate of only 12 nm $s^{-1}$ at $E_{sub}$ of -0.40 V, whereas double-barrel printing at -0.30 V $V_{bias}$ takes place at an order of magnitude higher rate of 159 nm $s^{-1}$. For Pt-rich compositions, the single-barrel configuration achieved 45 nm $s^{-1}$ at -0.95 V, while the double-barrel method reached 57 nm $s^{-1}$ at +0.30 V $V_{bias}$, both at -0.95 V $E_{sub}$ respectively. The print rate increase in double-barrel configuration is qualitatively confirmed by our simulations (Figure SI-10). The data shows that mass-fluxes (and print rates) can be significantly enhanced (or suppressed) as compared to the case with 0 V bias, which here would represent a single barrel case.

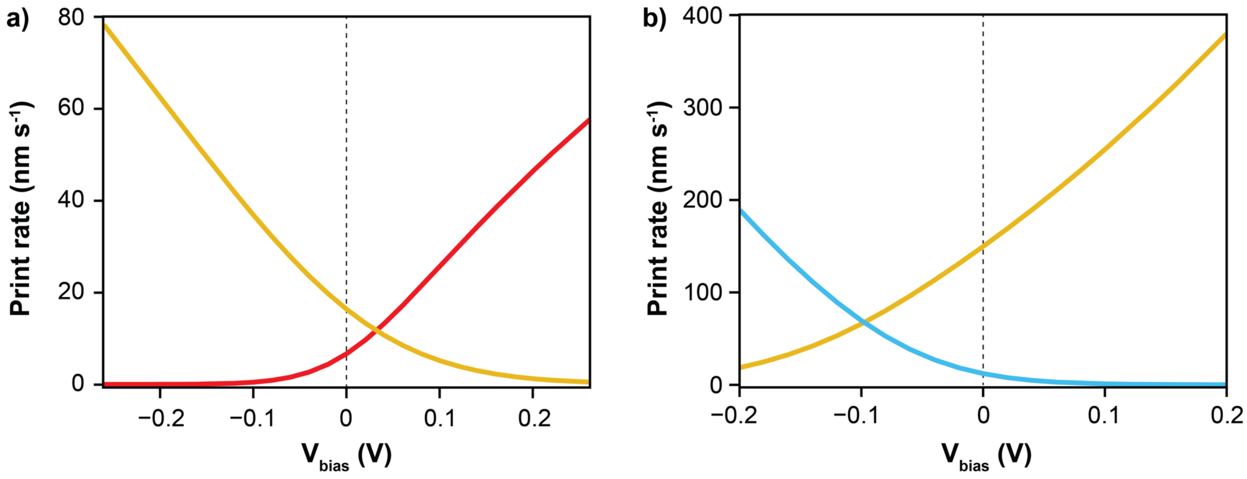


**Figure SI-10:** Print rates calculated from simulated fluxes for a) Au (yellow) and Cu (red) and b) Au (yellow) and Pt (blue) vs $V_{bias}$ in Au-Cu and Au-Pt systems, respectively, using Faraday's law.

## SI-7. EDX analysis of Pt-Au material gradient pillar

To validate gradual material composition change in the Pt-Au (top to bottom) gradient pillar, quantitative high-resolution EDX mapping was performed along the pillar axis. The overlay plot in Figure SI-11 shows the elemental fractions (at%), with Au represented in yellow and Pt in blue. At the Au-rich bottom of the pillar the composition reaches about 91 at% Au, while at the Pt-rich top, the Au amount gradually drops to 63 at%.

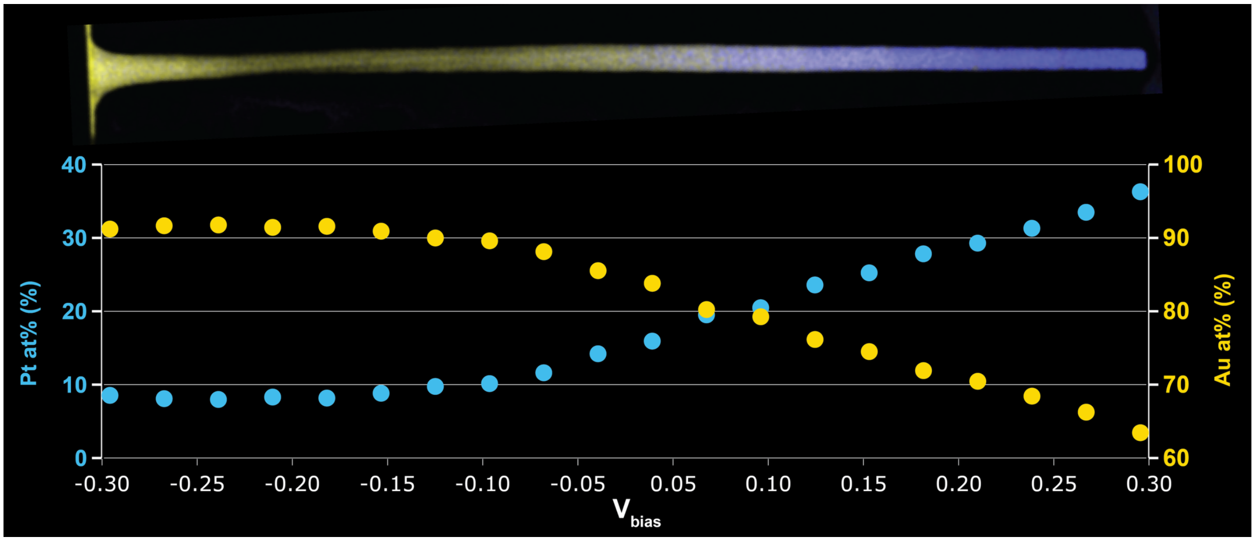


**Figure SI-11:** Quantitative EDX mapping of an Pt-Au (top to bottom) gradient pillar. The overlay plot shows elemental composition in at%, with Au indicated in yellow and Pt in blue.

## SI-8. Sharp compositional switching

The rapid composition change can be observed from microstructural features, print data, and EDX analysis. Figure SI-12a, for example, shows a clear change in the morphology that takes place upon switching, suggesting that this can occur as fast as within one (or maybe only a few) deposited layer(s). To confirm this visual observation from HR-TEM, one can also note the corresponding differences in electrodeposition data (Figure SI-12b), which shows current vs time and z piezo position vs time print data (Figure SI-12b). This shows the deposition duration of one layer with about 11 ms (see inset in Figure SI-12b). The bias change took place during the retract phase between two voxels which occurs within a longer time (about 1.6 s) due to the repositioning (retraction followed by reapproaching) of the nozzle between the voxels. As seen from the current signatures and vertical piezo data there is a sharp clear change that occurs upon switching the bias, provided by a clear distinction between Au and Pt current signatures and within a layer with thickness of ca. 21 nm, see inset in Figure SI-12c. Corresponding EDX measurements also show

sharp compositional switching (Figure SI-12c), although since the segment borders are not perfectly straight, there is a certain broadening in line profiles shown in the bottom part of Figure SI-12c.

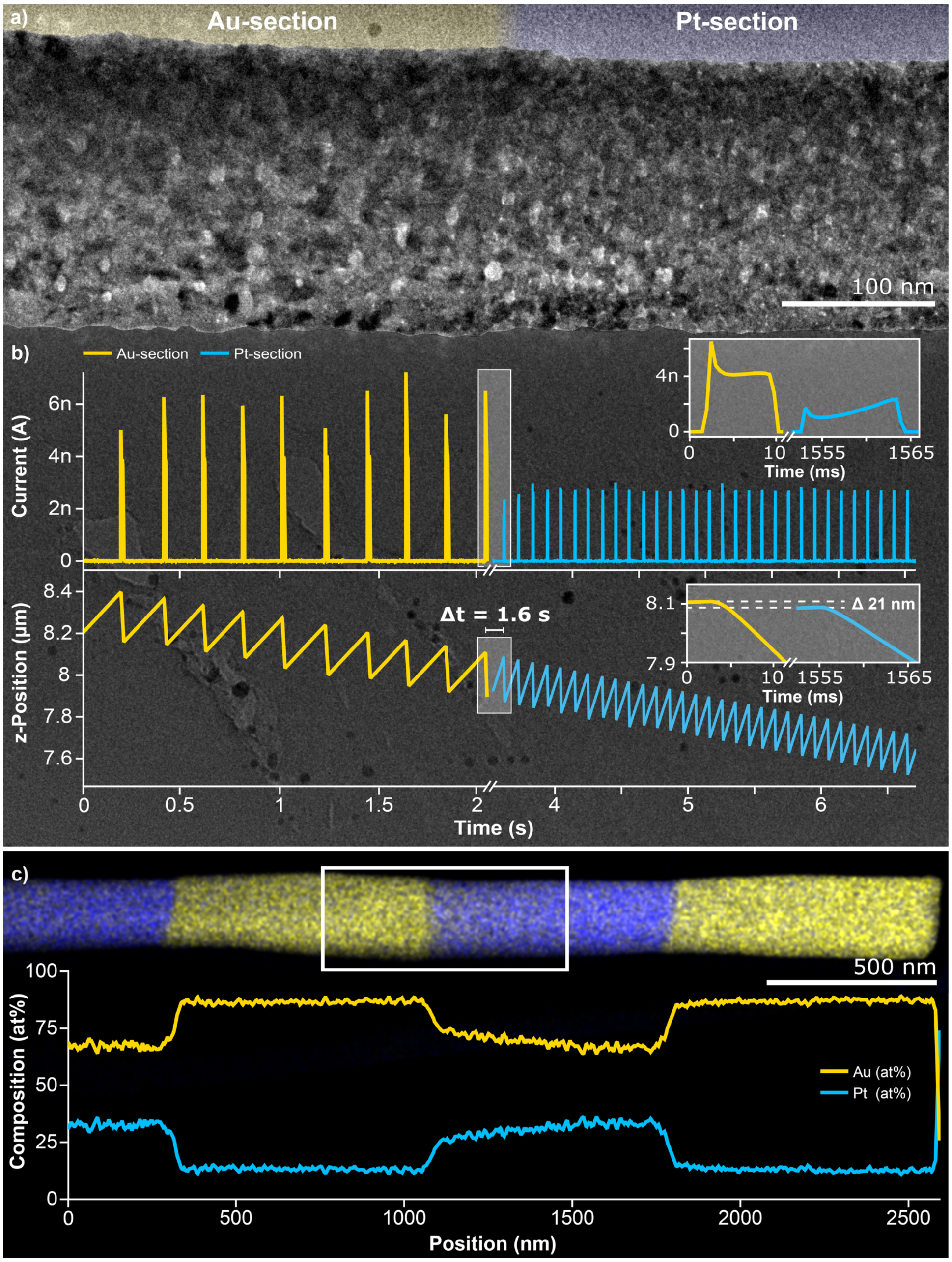


**Figure SI-12:** a) HR-TEM image overlayed with EDX composition data. b) Current and z-piezo position data showing switching between Au and Pt sections. c) STEM-EDX image and corresponding line profiles (averaged within the thickness of the pillar).

## SI-9. Adhesion of Au-Pt interface in iMCED deposition

The HR-TEM results of the Au–Pt alternating pillar (Figure 4a) showed no signs of adhesion issues changing the composition, however, this was sporadically observed printing single metal Pt pillars on an Au substrate (**Error! Reference source not found.** SI-13). While printing Au pillars on Au substrates resulted in good pillar adhesion to the substrate. Printing Pt pillars on similar substrates resulted in severe pillar delamination from the substrate in some structures, indicating a semi-stable material formation at the interface.

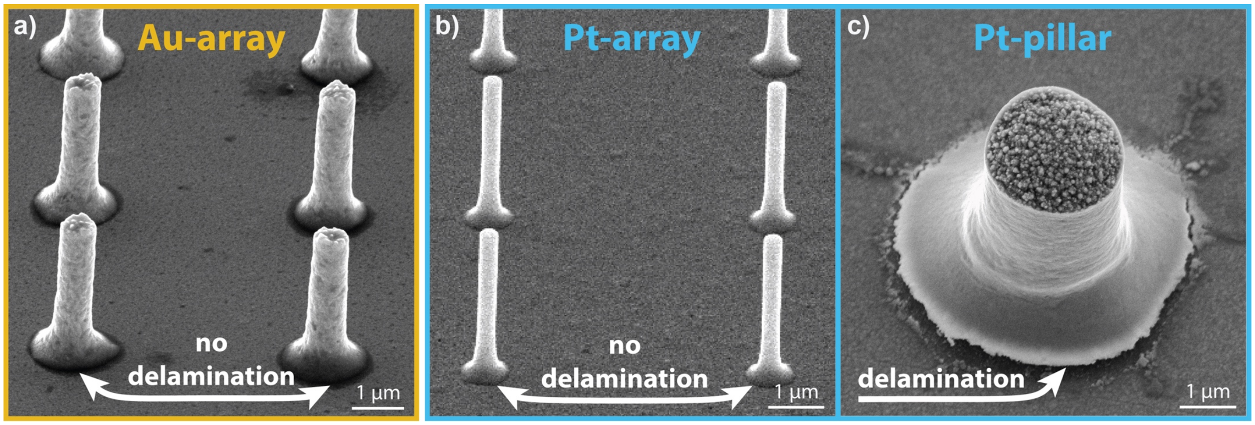


**Figure SI-13:** Comparison of single metal Au and Pt pillars printed with iMCED on an Au substrate visualizing the adhesion difference of (a) Au on Au and (b-c) Pt on Au. While Au printed on Au shows no delamination, deposition of Pt on Au sometimes is accompanied by delamination.

## SI-10. Microstructure of the 8-segmented "zebra-striped" and gradient pillar

The microstructure of the alternating 8-segmented Au-Pt "zebra-striped" pillar was examined by STEM-HAADF (a), STEM-BF (b), prepared by cutting a lamella and thinning it by focused ion beam (FIB) for electron transparency (c). Regions of interest highlighted in (c) were analyzed at higher magnification (d–f) for the green section and (g-i) for the blue highlighted section (Figure SI-14). Both inspected areas originate from Pt-rich sections, enabling a direct comparison of microstructural features within similar composition segments. The close-up images confirm a

polycrystalline structure with individual grains of ca. 10–20 nm, consistent across different segments. The crystallites appear densely packed and without observable porosity, supporting the conclusion that the iMCED process yields dense metal alloys.

The negligible atomic mobility in the iMCED-printed Au-Pt alloys can be quantitatively assessed from the diffusion coefficients of Au in Pt. Additionally, using the Arrhenius-based coefficients $D_{\mathrm{AuPt}} \approx 1.5 \cdot 10^{-23}$ cm$^2$ s$^{-1}$ at 600 K and $1.15 \cdot 10^{-16}$ cm$^2$ s$^{-1}$ at 600 K, the diffusion length $L = \sqrt{Dt}$ remains ca. 1 nm at 600 K (for $t = 10^3$ s) and only 3–6 nm at 900 K over 10–60 min ($4 \cdot 10^{-6}$ nm in 11 ms), i.e., far below what is required to partition 10–20 nm grains into equilibrium two-phase mixtures.

While thermal energy is the main driver for atomic rearrangement and phase segregation, electrochemical polarization can also act as a driver by altering surface electrochemical potential. However, in our deposition regime the metals are reduced under cathodic bias and remain far from dissolution conditions or equilibrium potential. Therefore, polarization does not provide electrochemically activated relaxation for the metastable alloy.

Furthermore, at the nanoscale, the thermodynamics of phase stability deviate from bulk behavior because the interfacial energy becomes a significant fraction of the total Gibbs free energy. The total free energy per unit volume of a two-phase system can be approximated as

$$G_{total} = x_\alpha G_\alpha + x_\beta G_\beta + \gamma A/V \qquad \textbf{Eq SI-12}$$

*$x_\alpha, x_\beta$: phase fractions ($x_\alpha + x_\beta = 1$); $G_\alpha, G_\beta$: bulk Gibbs free energies of each phase; $\gamma$: interfacial energy between the two phases (J/m$^2$); $A$: total interfacial area; $V$: total volume*

Then, $\gamma A/V$ represents the penalty associated with creating internal interfaces. In 10–20 nm grains, the $A/V$ratio is high, and the capillarity term can overcome bulk driving force for

segregation, thereby penalizing phase separation and effectively narrowing the miscibility gap. In other words, forming two phases would require the creation of numerous high-energy interfaces and strain within nanoscale grains, which is energetically less favorable than maintaining a homogeneous solid solution under the present deposition conditions.

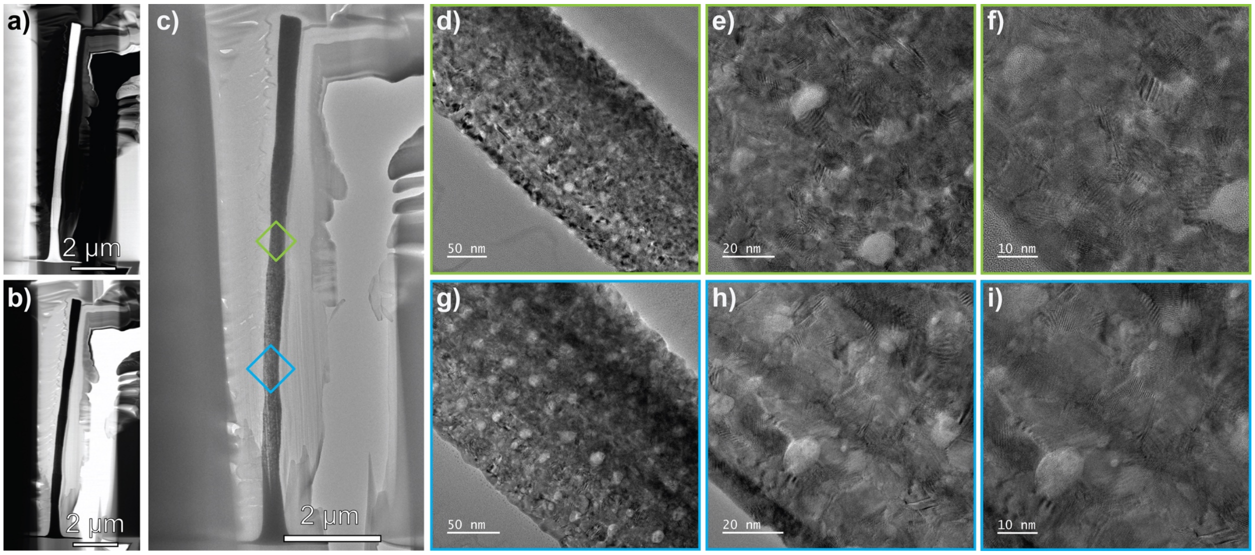


**Figure SI-14:** STEM analysis of a “zebra-striped” Au-Pt pillar visualized by (a) HAADF, (b) BF-STEM overview images, and (c) STEM lamella prepared for electron transmission with highlighted regions of two Pt-segments. (d–i) Close-ups from the color labeled Pt-segments

For the gradient pillar, the overview images (a–c) correspond to those shown in Figure SI-15. Three representative positions were analyzed in detail marked in (c): a Pt-rich regime (red, shown in d), a regime at 50% of the achieved material range of Pt (blue, shown in e), and an Au-rich regime (yellow, shown in f). Higher magnification of the Au-rich region (g–i) reveals its nanocrystalline morphology with grain sizes in the 10–20 nm range, consistent with the structures observed in the “zebra-striped” pillar. The comparison across Pt-, Au-, and intermediate regions shows that the alloy microstructure remains homogeneous throughout the gradient, with no detectable porosity or phase segregation over changing compositions.

Together, these results confirm that both compositionally discrete ("zebra") and continuously graded (gradient) Au-Pt structures are non-porous, polycrystalline alloys with nanoscale grain sizes, further corroborating the conclusions drawn from the main manuscript (Figure 4c–e).

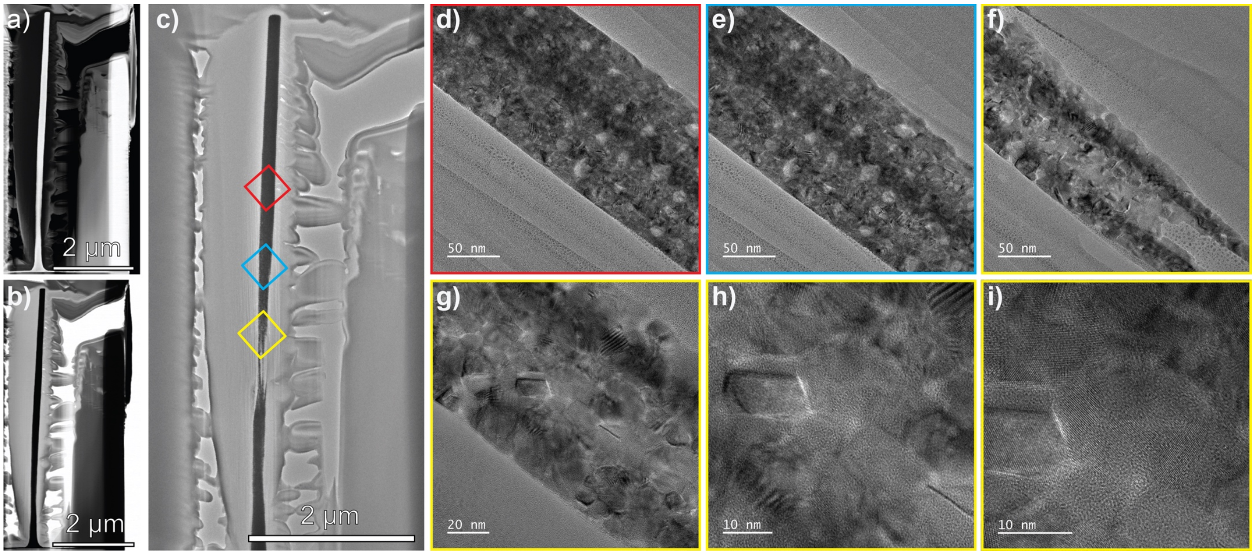


**Figure SI-15:** STEM analysis of the Pt-Au (top to bottom) gradient pillar visualized by (a) HAADF, (b) BF-STEM overview images, and (c) STEM lamella prepared for electron transmission with highlighted regions of different compositions. For microstructure comparison, (d) Pt-rich region, (e) region of intermediate material range composition, and (f) Au-rich region are highlighted in (c). (g–i) Close-ups from the color-labeled region shown in (f).

**SI-11. Equilibrium phase diagram of Au–Pt**

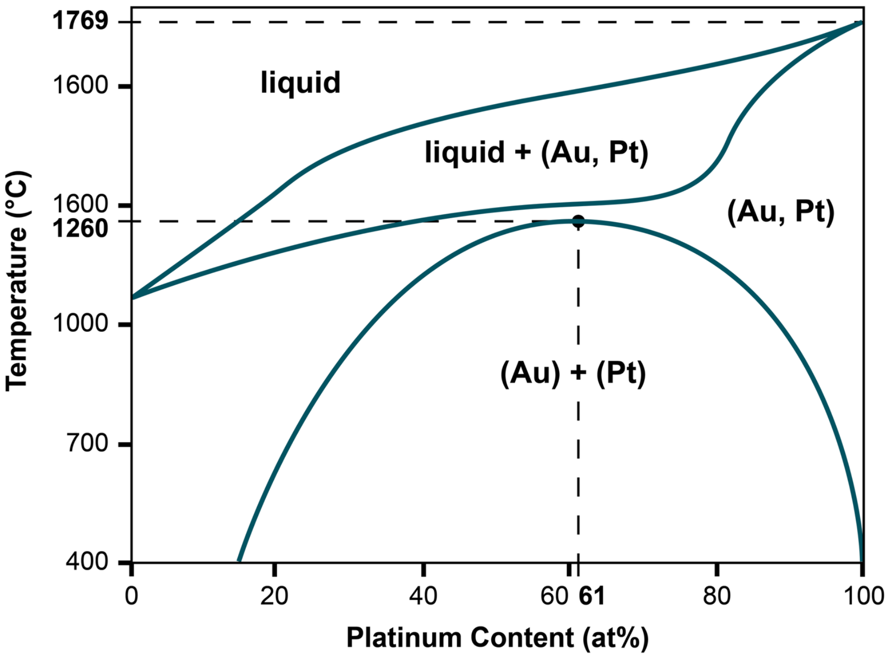


**Figure SI-16:** Equilibrium phase diagram of Au–Pt recreated after H. Okamoto and T. B. Massalski[4].

**SI-12. Selected area electron diffraction analysis**

Selected area electron diffraction (SAED) was performed on individual segments of the "zebra-striped" Pt-Au (top to bottom) pillar to assess crystallinity and phase composition. The corresponding diffraction patterns are shown in Figure SI-17 to the related "zebra-segments", with associated d-spacing values. The d-spacings were determined for the closest diffraction ring and correlated to the 111 plane. A correction factor for lens aberrations of 0.9968 was applied for the first measurement, while a lens aberration factor of 0.9893 corrected the second measurement.

All recorded patterns display well-defined diffraction rings, confirming the polycrystalline nature of the printed structures. Analysis of Pt-rich regions yields a first diffraction ring corresponding to a d-spacing of 2.319 Å, which translates to an alloy composition of 61 at% Au and 39 at% Pt using Vegard's law. This is in an excellent agreement also with the EDX data, confirming that Au-Pt in this case form a solid solution (single phase alloy) without phase

separation. In contrast, Au-rich regions exhibit a slightly larger d-spacing of 2.352 Å, which would translate into alloy composition of 97 at% Au and 3 at% Pt. However, EDX composition suggests somewhat higher amount of Pt in the structure (13-15%, depending on the segment). This suggests that Pt is only partly mixed with Au forming a phase with 3% Pt amount, while the rest of Pt content (10-12%) is not forming 111 Au-Pt phase.

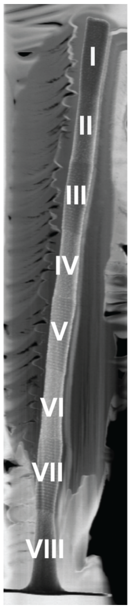


| Segment | d-spacing (Å) 111 diffr. plane | Composition according to Vegard's law from d-spacing data | | Composition according to HR-STEM EDX | |
|---|---|---|---|---|---|
| | | Pt (at%) | Au (at%) | Pt (at%) | Au (at%) |
| Au I | 2.352 | 3 | 97 | 13 | 87 |
| Pt II | 2.319 | 39 | 61 | 39 | 61 |
| Au III | 2.352 | 3 | 97 | 14 | 86 |
| Pt IV | 2.319 | 39 | 61 | 39 | 61 |
| Au V | 2.352 | 3 | 97 | 15 | 85 |
| Pt VI | 2.319 | 39 | 61 | 40 | 60 |
| Au VII | 2.352 | 3 | 97 | 15 | 85 |
| Pt VIII | 2.319 | 39 | 61 | 41 | 59 |

1 µm

10 $nm^{-1}$

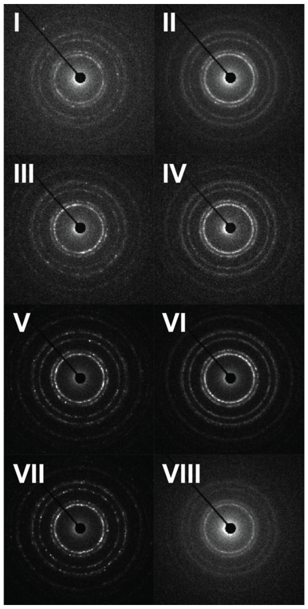


**Figure SI-17:** Selected area electron diffraction (SAED) analysis of a "zebra-striped" Pt-Au (top to bottom) pillar. Left: STEM overview of the analyzed lamella, showing positions of I–VIII used for analysis. Center: table summarizing measured d-spacings of the 111 diffraction plane (right) and corresponding alloy compositions derived using Vegard's law. Right: diffraction patterns from the individual segments, all showing well-defined rings characteristic of polycrystalline single-phase fcc alloys.

The stability of the crystal structure was confirmed by repeating the measurement after ca. 6 months post the initial characterization. The results of both measurements are presented in Figure SI-18.

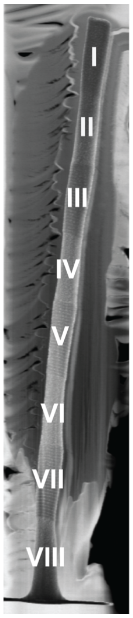


| | First analysis | Second analysis 6 month later | Composition according to Vegard's law from d-spacing data: First analysis | | Composition according to Vegard's law from d-spacing data: Second analysis 6 month later | |
|---|---|---|---|---|---|---|
| Segment | d-spacing (Å) 111 diffr. plane | d-spacing (Å) 111 diffr. plane | Pt (at%) | Au (at%) | Pt (at%) | Au (at%) |
| Au I | 2.352 | 2.352 | 3 | 97 | 3 | 97 |
| Pt II | 2.319 | 2.319 | 39 | 61 | 39 | 61 |
| Au III | 2.352 | 2.352 | 3 | 97 | 3 | 97 |
| Pt IV | 2.319 | 2.319 | 39 | 61 | 39 | 61 |
| Au V | 2.352 | 2.352 | 3 | 97 | 3 | 97 |
| Pt VI | 2.319 | 2.319 | 39 | 61 | 39 | 61 |
| Au VII | 2.352 | 2.352 | 3 | 97 | 3 | 97 |
| Pt VIII | 2.319 | 2.319 | 39 | 61 | 39 | 61 |

**Figure SI-18:** Selected area electron diffraction (SAED) analysis of a "zebra-striped" Pt-Au (top to bottom) pillar. Left: STEM overview of the analyzed lamella, showing positions of I–VIII used for analysis. Center: table summarizing measured d-spacings of the 111 diffraction plane obtained from first analysis compared to identical analysis performed six months after. Alloy compositions derived using Vegard's law from d-spacing data are compared from first analysis to six months later for time dependent change resulting in identical values.

## References

(1) Zhou, M.; Dick, J. E.; Bard, A. J. Electrodeposition of Isolated Platinum Atoms and Clusters on Bismuth-Characterization and Electrocatalysis. *J. Am. Chem. Soc.* **2017**, *139* (48), 17677-17682. DOI: 10.1021/jacs.7b10646.

(2) Lide, D. R. CRC Handbook of Chemistry and Physics: A Ready-Reference Book of Chemical and Physical Data. 85 ed.; CRC Press: Boca Raton, London, New York, Washington D.C., 2004.

(3) Momotenko, D.; Cortes-Salazar, F.; Josserand, J.; Liu, S.; Shao, Y.; Girault, H. H. Ion current rectification and rectification inversion in conical nanopores: a perm-selective view. *Phys. Chem. Chem. Phys.* **2011**, *13* (12), 5430-5440. DOI: 10.1039/c0cp02595j.

(4) Okamoto, H.; Massalski, T. B. The Au–Pt (Gold-Platinum) system. *Bull. Alloy Phase Diagrams* **1985**, *6*, 46–56. DOI: 10.1007/BF02871187.
