# Supplementary material for: Additive Manufacturing of Nanoscale Multimaterial Voxels Via Meniscus-Confined Electrodeposition
Source: ACS Nano. 2026 Apr 8;20(15):11604–13. doi: 10.1021/acsnano.5c16931 (PMC13104162; doi:10.1021/acsnano.5c16931)

Comsol model report

Report date	Sep 25, 2025, 6:04:05 PM

Contents

1. Global Definitions 3
1.1. Parameters 3
1.2. Shared Properties 4
2. Component 1 5
2.1. Definitions 5
2.2. Geometry 1 6
2.3. Electric Currents 10
2.4. Transport of Diluted Species 32
2.5. Mesh 1 69
3. Study 1 75
3.1. Parametric Sweep 75
3.2. Stationary 75
3.3. Solver Configurations 76
4. Results 92
4.1. Datasets 92
4.2. Derived Values 92
4.3. Tables 93
4.4. Plot Groups 96

1 Global Definitions

Date	Sep 23, 2025, 3:39:31 PM

GLOBAL SETTINGS

Name	CuAu-Basic geometry 3 ec tds sub Vbias 2.mph
Path	D:\Dmitry\Simulation results\2025\Theta pipette\3D\TEM geometry\Corrected D values\CuAu-Basic geometry_3_ec_tds_sub_Vbias_2.mph
Version	COMSOL Multiphysics 6.2 (Build: 658)
Unit system	SI

USED PRODUCTS

COMSOL Multiphysics
Chemical Reaction Engineering Module

COMPUTER INFORMATION

CPU	Intel64 Family 6 Model 165 Stepping 5, 10 cores, 127.9 GB RAM
Operating system	Windows 10

1.1 PARAMETERS

PARAMETERS 1

Name	Expression	Value	Description
a	5.6[degree]	0.097738 rad	cone semi angle
c0Au	10[mmol/L]	10 mol/m^3	concentration of Au
c0Cu	10 [mmol/L]	10 mol/m^3	concentration of Pt
c0H2SO4	500[mmol/L]	500 mol/m^3	bulk h2so4 concentration
c0H2SO42	507.6[mmol/L]	507.6 mol/m^3	concentration of H2SO4 in empty barrek
DAu	1.2e-5[cm^2/s]	1.2E−9 m^2/s	diffusion coeff AuCl4-
DCu	0.714e-5[cm^2/s]	7.14E−10 m^2/s	diffusion coeff Cu2+
DH	9.31e-9[m^2/s]	9.31E−9 m^2/s	diffusion coeff of H+
DSO4	1.065e-5[cm^2/s]	1.065E−9 m^2/s	diffusion coefficient SO4 2-
F	96485[C/mol]	96485 C/mol	Faraday constant
L	20[um]	2E−5 m	pipette length
offset	0[nm]	0 m	septum offset
R	8.31[J/mol/K]	8.31 J/(mol·K)	gas constant
rtip	160[nm]	1.6E−7 m	total tip size
T	298.15[K]	298.15 K	temperature
uAu	1*F*DAu/R/T	4.6731E−8 m^2/(V·s)	mobility of Au ions

Name	Expression	Value	Description
uCu	2*F*DCu/R/T	5.561E−8 $m^2/(V \cdot s)$	mobility of Pt ions
uH	1*F*DH/R/T	3.6255E−7 $m^2/(V \cdot s)$	mobility of H+
uSO4	2*F*DSO4/R/T	8.2948E−8 $m^2/(V \cdot s)$	mobility of SO4 2-
Vempty	-0.75[V]	−0.75 V	potential in left barrel
Vmetal	-1.05[V]	−1.05 V	potential in right barrel
wth	45[nm]	4.5E−8 m	wall thickness

1.2 SHARED PROPERTIES

1.2.1 Default Model Inputs

Tag	cminpt

2 Component 1

Date	Jul 14, 2025, 1:47:16 PM

SETTINGS

Description	Value
Unit system	Same as global system (SI)
Geometry shape function	Automatic

SPATIAL FRAME COORDINATES

First	Second	Third
x	y	z

MATERIAL FRAME COORDINATES

First	Second	Third
X	Y	Z

GEOMETRY FRAME COORDINATES

First	Second	Third
Xg	Yg	Zg

MESH FRAME COORDINATES

First	Second	Third
Xm	Ym	Zm

2.1 DEFINITIONS

2.1.1 Coordinate Systems

Boundary System 1

Coordinate system type	Boundary system
Tag	sys1

COORDINATE NAMES

First	Second	Third
t1	t2	n

2.2 GEOMETRY 1

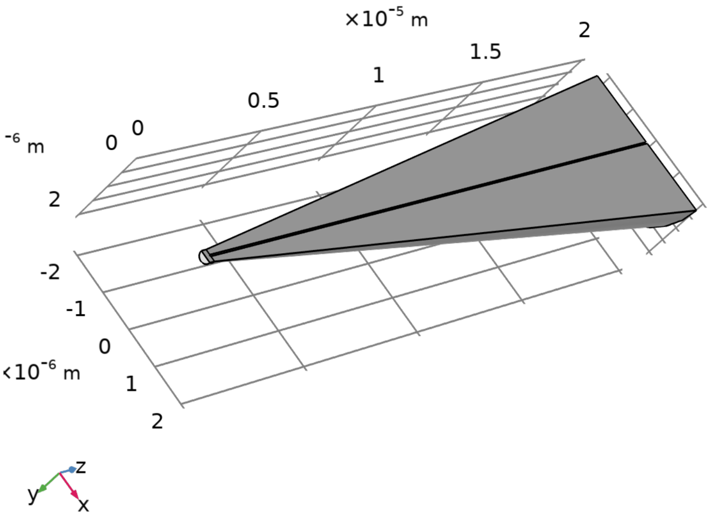

Geometry 1

UNITS

Length unit	m
Angular unit	deg

GEOMETRY STATISTICS

Description	Value
Space dimension	3
Number of domains	3
Number of boundaries	16
Number of edges	29
Number of vertices	17

2.2.1 Cone 1 (cone1)

POSITION

Description	Value
Position	{0, 0, rtip + wth/2}

AXIS

Description	Value
Axis type	z - axis

SIZE AND SHAPE

Description	Value
Bottom radius	rtip
Height	L
Specify top size using	Radius
Top radius	rtip + L*tan(a)

INFORMATION

Description	Value
Last build time	< 1 second
Built with	COMSOL 6.2.0.658 (win64), Jul 28, 2025, 2:35:11 PM

2.2.2 Block 1 (blk1)

POSITION

Description	Value
Position	{-(rtip + L*tan(a)), 0, rtip + wth/2}

AXIS

Description	Value
Axis type	z - axis

SIZE AND SHAPE

Description	Value
Width	2*(rtip + L*tan(a))
Depth	2*(rtip + L*tan(a))
Height	L

INFORMATION

Description	Value
Last build time	< 1 second
Built with	COMSOL 6.2.0.658 (win64), Jul 28, 2025, 2:35:11 PM

2.2.3 Intersection 1 (int1)

INFORMATION

Description	Value
Last build time	< 1 second
Built with	COMSOL 6.2.0.658 (win64), Jul 28, 2025, 2:35:11 PM

2.2.4 Block 2 (blk2)

POSITION

Description	Value
Position	{-wth/2, 0, rtip + wth/2}

AXIS

Description	Value
Axis type	z - axis

SIZE AND SHAPE

Description	Value
Width	wth
Depth	(rtip + L*tan(a))
Height	L

INFORMATION

Description	Value
Last build time	< 1 second
Built with	COMSOL 6.2.0.658 (win64), Jul 28, 2025, 2:35:11 PM

2.2.5 Difference 1 (dif1)

INFORMATION

Description	Value
Last build time	< 1 second
Built with	COMSOL 6.2.0.658 (win64), Jul 28, 2025, 2:35:11 PM

2.2.6 Cylinder 1 (cyl1)

POSITION

Description	Value
Position	{0, 0, 0}

AXIS

Description	Value
Axis type	z - axis

SIZE AND SHAPE

Description	Value
Radius	rtip
Height	rtip + wth/2

INFORMATION

Description	Value
Last build time	< 1 second
Built with	COMSOL 6.2.0.658 (win64), Jul 28, 2025, 2:35:11 PM

2.2.7 Block 3 (blk3)

POSITION

Description	Value
Position	{-(rtip + L*tan(a)), 0, 0}

AXIS

Description	Value
Axis type	z - axis

SIZE AND SHAPE

Description	Value
Width	2*(rtip + L*tan(a))
Depth	2*(rtip + L*tan(a))
Height	L

INFORMATION

Description	Value
Last build time	< 1 second
Built with	COMSOL 6.2.0.658 (win64), Jul 28, 2025, 2:35:11 PM

2.2.8 Intersection 2 (int2)

INFORMATION

Description	Value
Last build time	< 1 second
Built with	COMSOL 6.2.0.658 (win64), Jul 28, 2025, 2:35:11 PM

2.2.9 Cylinder 2 (cyl2)

POSITION

Description	Value
Position	{0, 0, rtip + wth/2}

AXIS

Description	Value
Axis type	y - axis

SIZE AND SHAPE

Description	Value
Radius	wth/2
Height	2*rtip

INFORMATION

Description	Value
Last build time	< 1 second
Built with	COMSOL 6.2.0.658 (win64), Jul 28, 2025, 2:35:11 PM

2.2.10 Difference 2 (dif2)

INFORMATION

Description	Value
Last build time	< 1 second
Built with	COMSOL 6.2.0.658 (win64), Jul 28, 2025, 2:35:11 PM

2.2.11 Form Union (fin)

INFORMATION

Description	Value
Details	{Formed union of 2 solid objects., Union has 3 domains, 16 boundaries, 29 edges, and 17 vertices.}
Last build time	< 1 second
Built with	COMSOL 6.2.0.658 (win64), Jul 28, 2025, 2:35:11 PM

2.3 ELECTRIC CURRENTS

USED PRODUCTS

COMSOL Multiphysics

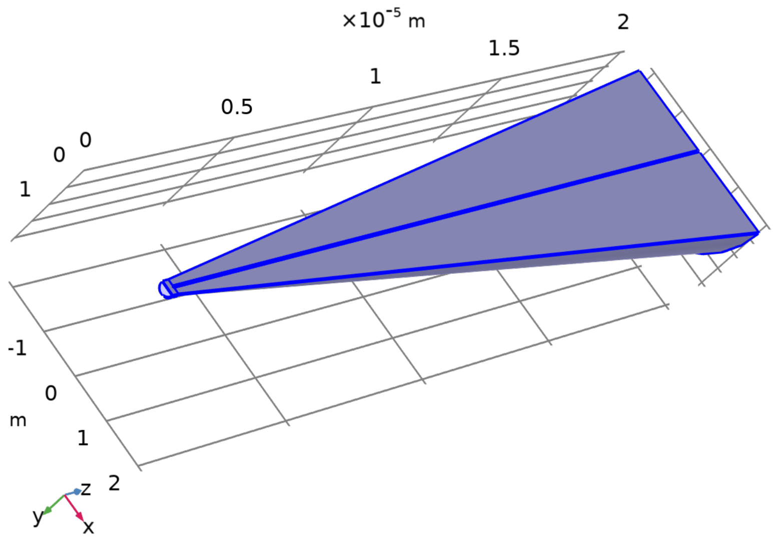

Electric Currents

SELECTION

Geometric entity level	Domain
Selection	Geometry geom1: Dimension 3: All domains

EQUATIONS

$$\nabla \cdot \mathbf{J} = Q_{j,v}$$

$$\mathbf{J} = \sigma \mathbf{E} + \mathbf{J}_e$$

$$\mathbf{E} = -\nabla V$$

2.3.1 Interface Settings

Discretization

SETTINGS

Description	Value
Electric potential	Quadratic

SETTINGS

Description	Value
Equation form	Study controlled

Manual Terminal Sweep Settings

SETTINGS

Description	Value	Unit
Use manual terminal sweep	Off	

Description	Value	Unit
Reference impedance	50	Ω

2.3.2 Variables

Name	Expression	Unit	Description	Selection	Details
ec.d	1	1	Contribution	Domains 1–3	
ec.I_sXX	(spatial.invF11*(spatial.invF11*ec.I_sxx+spatial.invF21*ec.I_syx+spatial.invF31*ec.I_szx)+spatial.invF21*(spatial.invF11*ec.I_sxy+spatial.invF21*ec.I_syy+spatial.invF31*ec.I_szy)+spatial.invF31*(spatial.invF11*ec.I_sxz+spatial.invF21*ec.I_syz+spatial.invF31*ec.I_szz))*spatial.detF	1	Spatial identity matrix, material frame, XX-component	Domains 1–3	
ec.I_sYX	(spatial.invF11*(spatial.invF12*ec.I_sxx+spatial.invF22*ec.I_syx+spatial.invF32*ec.I_szx)+spatial.invF21*(spatial.invF12*ec.I_sxy+spatial.invF22*ec.I_syy+spatial.invF32*ec.I_szy)+spatial.invF31*(spatial.invF12*ec.I_sxz+spatial.invF22*ec.I_syz+spatial.invF32*ec.I_szz))*spatial.detF	1	Spatial identity matrix, material frame, YX-component	Domains 1–3	
ec.I_sZX	(spatial.invF11*(spatial.invF13*ec.I_sxx+spatial.invF23*ec.I_syx+spatial.invF33*ec.I_szx)+spatial.invF21*(spatial.invF13*ec.I_sxy+spatial.invF23*ec.I_syy+spatial.invF33*ec.I_szy)+spatial.invF31*(spatial.invF13*ec.I_sxz+spatial.invF23*ec.I_syz+spatial.invF33*ec.I_szz))*spatial.detF	1	Spatial identity matrix, material frame, ZX-component	Domains 1–3	
ec.I_sXY	(spatial.invF12*(spatial.invF11*ec.I_sxx+spatial.invF21*ec.I_syx+spatial.invF31*ec.I_szx)+spatial.	1	Spatial identity matrix, material frame, XY-component	Domains 1–3	

Name	Expression	Unit	Description	Selection	Details
	invF22*(spatial.invF11*ec.I_sxy+spatial.invF21*ec.I_syy+spatial.invF31*ec.I_szy)+spatial.invF32*(spatial.invF11*ec.I_sxz+spatial.invF21*ec.I_syz+spatial.invF31*ec.I_szz))*spatial.detF				
ec.I_sYY	(spatial.invF12*(spatial.invF12*ec.I_sxx+spatial.invF22*ec.I_syx+spatial.invF32*ec.I_szx)+spatial.invF22*(spatial.invF12*ec.I_sxy+spatial.invF22*ec.I_syy+spatial.invF32*ec.I_szy)+spatial.invF32*(spatial.invF12*ec.I_sxz+spatial.invF22*ec.I_syz+spatial.invF32*ec.I_szz))*spatial.detF	1	Spatial identity matrix, material frame, YY-component	Domains 1–3	
ec.I_sZY	(spatial.invF12*(spatial.invF13*ec.I_sxx+spatial.invF23*ec.I_syx+spatial.invF33*ec.I_szx)+spatial.invF22*(spatial.invF13*ec.I_sxy+spatial.invF23*ec.I_syy+spatial.invF33*ec.I_szy)+spatial.invF32*(spatial.invF13*ec.I_sxz+spatial.invF23*ec.I_syz+spatial.invF33*ec.I_szz))*spatial.detF	1	Spatial identity matrix, material frame, ZY-component	Domains 1–3	
ec.I_sXZ	(spatial.invF13*(spatial.invF11*ec.I_sxx+spatial.invF21*ec.I_syx+spatial.invF31*ec.I_szx)+spatial.invF23*(spatial.invF11*ec.I_sxy+spatial.invF21*ec.I_syy+spatial.invF31*ec.I_szy)+spatial.invF33*(spatial.invF11*ec.I_sxz+spatial.invF21*ec.I_syz+spatial.invF31*ec.I_szz))*spatial.detF	1	Spatial identity matrix, material frame, XZ-component	Domains 1–3	
ec.I_sYZ	(spatial.invF13*(spatial.invF12*ec.I_sxx+spatial.i	1	Spatial identity matrix, material	Domains 1–3	

Name	Expression	Unit	Description	Selection	Details
	nvF22*ec.I_syx+spatial.invF32*ec.I_szx)+spatial.invF23*(spatial.invF12*ec.I_sxy+spatial.invF22*ec.I_syy+spatial.invF32*ec.I_szy)+spatial.invF33*(spatial.invF12*ec.I_sxz+spatial.invF22*ec.I_syz+spatial.invF32*ec.I_szz))*spatial.detF		frame, YZ-component		
ec.I_sZZ	(spatial.invF13*(spatial.invF13*ec.I_sxx+spatial.invF23*ec.I_syx+spatial.invF33*ec.I_szx)+spatial.invF23*(spatial.invF13*ec.I_sxy+spatial.invF23*ec.I_syy+spatial.invF33*ec.I_szy)+spatial.invF33*(spatial.invF13*ec.I_sxz+spatial.invF23*ec.I_syz+spatial.invF33*ec.I_szz))*spatial.detF	1	Spatial identity matrix, material frame, ZZ-component	Domains 1–3	
ec.I_sxx	1	1	Spatial identity matrix, xx-component	Domains 1–3	
ec.I_syx	0	1	Spatial identity matrix, yx-component	Domains 1–3	
ec.I_szx	0	1	Spatial identity matrix, zx-component	Domains 1–3	
ec.I_sxy	0	1	Spatial identity matrix, xy-component	Domains 1–3	
ec.I_syy	1	1	Spatial identity matrix, yy-component	Domains 1–3	
ec.I_szy	0	1	Spatial identity matrix, zy-component	Domains 1–3	
ec.I_sxz	0	1	Spatial identity matrix, xz-component	Domains 1–3	

Name	Expression	Unit	Description	Selection	Details
ec.I_syz	0	1	Spatial identity matrix, yz-component	Domains 1–3	
ec.I_szz	1	1	Spatial identity matrix, zz-component	Domains 1–3	
ec.nx	nx		Normal vector, x-component	Boundaries 7, 14	
ec.ny	ny		Normal vector, y-component	Boundaries 7, 14	
ec.nz	nz		Normal vector, z-component	Boundaries 7, 14	
ec.nx	dnx		Normal vector, x-component	Boundaries 1–6, 8–13, 15–16	
ec.ny	dny		Normal vector, y-component	Boundaries 1–6, 8–13, 15–16	
ec.nz	dnz		Normal vector, z-component	Boundaries 1–6, 8–13, 15–16	
ec.nmeshx	nxmesh		Mesh normal vector, x-component	Boundaries 7, 14	
ec.nmeshy	nymesh		Mesh normal vector, y-component	Boundaries 7, 14	
ec.nmeshz	nzmesh		Mesh normal vector, z-component	Boundaries 7, 14	
ec.nmeshx	dnxmesh		Mesh normal vector, x-component	Boundaries 1–6, 8–13, 15–16	
ec.nmeshy	dnymesh		Mesh normal vector, y-component	Boundaries 1–6, 8–13, 15–16	
ec.nmeshz	dnzmesh		Mesh normal vector, z-component	Boundaries 1–6, 8–13, 15–16	
ec.unmeshx	unxmesh		Mesh normal vector, upside, x-component	Boundaries 1–16	

Name	Expression	Unit	Description	Selection	Details
ec.unmeshy	unymesh		Mesh normal vector, upside, y-component	Boundaries 1–16	
ec.unmeshz	unzmesh		Mesh normal vector, upside, z-component	Boundaries 1–16	
ec.dnmeshx	dnxmesh		Mesh normal vector, downside, x-component	Boundaries 1–16	
ec.dnmeshy	dnymesh		Mesh normal vector, downside, y-component	Boundaries 1–16	
ec.dnmeshz	dnzmesh		Mesh normal vector, downside, z-component	Boundaries 1–16	
ec.unTx	ec.unTex	Pa	Maxwell upward surface stress tensor, x-component	Boundaries 1–16	
ec.unTy	ec.unTey	Pa	Maxwell upward surface stress tensor, y-component	Boundaries 1–16	
ec.unTz	ec.unTez	Pa	Maxwell upward surface stress tensor, z-component	Boundaries 1–16	
ec.dnTx	ec.dnTex	Pa	Maxwell downward surface stress tensor, x-component	Boundaries 1–16	
ec.dnTy	ec.dnTey	Pa	Maxwell downward surface stress tensor, y-component	Boundaries 1–16	
ec.dnTz	ec.dnTez	Pa	Maxwell downward surface stress tensor, z-component	Boundaries 1–16	

Name	Expression	Unit	Description	Selection	Details
ec.unx	unx		Normal vector up direction, x-component	Boundaries 1–16	
ec.uny	uny		Normal vector up direction, y-component	Boundaries 1–16	
ec.unz	unz		Normal vector up direction, z-component	Boundaries 1–16	
ec.dnx	dnx		Normal vector down direction, x-component	Boundaries 1–16	
ec.dny	dny		Normal vector down direction, y-component	Boundaries 1–16	
ec.dnz	dnz		Normal vector down direction, z-component	Boundaries 1–16	
ec.unTex	-0.5*ec.dnx*(real(up(ec.Dx))*real(up(ec.Ex))+real(up(ec.Dy))*real(up(ec.Ey))+real(up(ec.Dz))*real(up(ec.Ez)))+real(up(ec.Dx))*(real(up(ec.Ex))*ec.dnx+real(up(ec.Ey))*ec.dny+real(up(ec.Ez))*ec.dnz)	Pa	Maxwell upward electric surface stress tensor, x-component	Boundaries 7, 14	
ec.unTey	-0.5*ec.dny*(real(up(ec.Dx))*real(up(ec.Ex))+real(up(ec.Dy))*real(up(ec.Ey))+real(up(ec.Dz))*real(up(ec.Ez)))+real(up(ec.Dy))*(real(up(ec.Ex))*ec.dnx+real(up(ec.Ey))*ec.dny+real(up(ec.Ez))*ec.dnz)	Pa	Maxwell upward electric surface stress tensor, y-component	Boundaries 7, 14	
ec.unTez	-0.5*ec.dnz*(real(up(ec.Dx))*real(up(ec.Ex))+real(up(ec.Dy))*real(up(ec.Ey))+real(up(ec.Dz))*real(up(ec.Ez)))+real(up(ec.Dz))*(real(up(ec.Ex))*ec.	Pa	Maxwell upward electric surface stress tensor, z-component	Boundaries 7, 14	

Name	Expression	Unit	Description	Selection	Details
	dnx+real(up(ec.Ey))*ec.dny+real(up(ec.Ez))*ec.dnz)				
ec.unTex	0	Pa	Maxwell upward electric surface stress tensor, x-component	Boundaries 1–6, 8–13, 15–16	
ec.unTey	0	Pa	Maxwell upward electric surface stress tensor, y-component	Boundaries 1–6, 8–13, 15–16	
ec.unTez	0	Pa	Maxwell upward electric surface stress tensor, z-component	Boundaries 1–6, 8–13, 15–16	
ec.dnTex	-0.5*ec.unx*(real(down(ec.Dx))*real(down(ec.Ex))+real(down(ec.Dy))*real(down(ec.Ey))+real(down(ec.Dz))*real(down(ec.Ez)))+real(down(ec.Dx))*(real(down(ec.Ex))*ec.unx+real(down(ec.Ey))*ec.uny+real(down(ec.Ez))*ec.unz)	Pa	Maxwell downward electric surface stress tensor, x-component	Boundaries 1–16	
ec.dnTey	-0.5*ec.uny*(real(down(ec.Dx))*real(down(ec.Ex))+real(down(ec.Dy))*real(down(ec.Ey))+real(down(ec.Dz))*real(down(ec.Ez)))+real(down(ec.Dy))*(real(down(ec.Ex))*ec.unx+real(down(ec.Ey))*ec.uny+real(down(ec.Ez))*ec.unz)	Pa	Maxwell downward electric surface stress tensor, y-component	Boundaries 1–16	
ec.dnTez	-0.5*ec.unz*(real(down(ec.Dx))*real(down(ec.Ex))+real(down(ec.Dy))*real(down(ec.Ey))+real(down(ec.Dz))*real(down(ec.Ez)))+real(down(ec.Dz))*(real(down(ec.Ex))*ec.	Pa	Maxwell downward electric surface stress tensor, z-component	Boundaries 1–16	

Name	Expression	Unit	Description	Selection	Details
	unx+real(down(ec.Ey))*ec.uny+real(down(ec.Ez))*ec.unz)				
ec.intWe	ec.int_We(ec.d*ec.dWe)	J	Total electric energy	Global	+ operation
ec.Qh	0	W/m³	Volumetric loss density, electromagnetic	Domains 1–3	
ec.Qsh	0	W/m²	Surface loss density, electromagnetic	Boundaries 1–16	
ec.Qlh	0	W/m	Line loss density, electromagnetic	Edges 1–29	
ec.CPx	ec.int_CP(x)/ec.int_CP(1)	m	Physics selection center point, spatial frame, x-component	Global	
ec.CPy	ec.int_CP(y)/ec.int_CP(1)	m	Physics selection center point, spatial frame, y-component	Global	
ec.CPz	ec.int_CP(z)/ec.int_CP(1)	m	Physics selection center point, spatial frame, z-component	Global	
ec.zref	50[ohm]	Ω	Reference impedance	Global	

2.3.3 Current Conservation 1

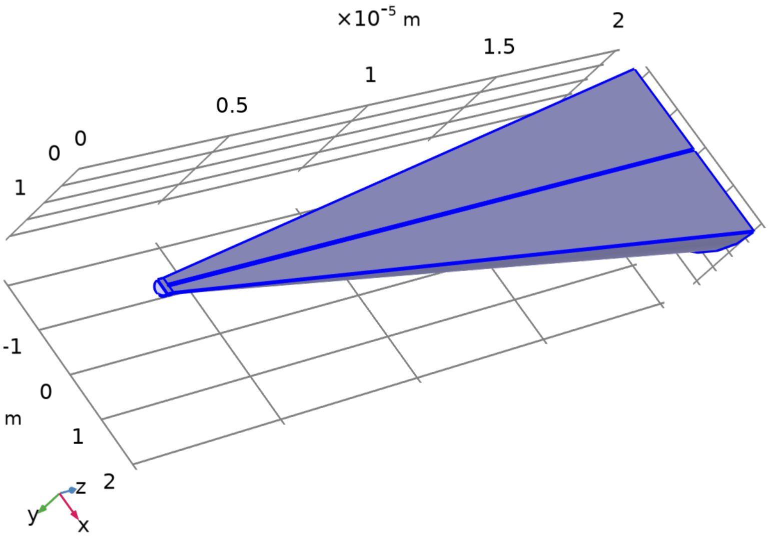

Current Conservation 1

SELECTION

Geometric entity level	Domain
Selection	Geometry geom1: Dimension 3: All domains

EQUATIONS

$$\nabla \cdot \mathbf{J} = Q_{j,v}$$

$$\mathbf{J} = \sigma \mathbf{E} + \mathbf{J}_e$$

$$\mathbf{E} = -\nabla V$$

Constitutive Relation Jc-E

SETTINGS

Description	Value	Unit
Conduction model	Electrical conductivity	
Electrical conductivity	User defined	
Electrical conductivity	F*(uH*cH + 2*uCu*cCu + uAu*cAu + 2*uSO4*cSO4)	S/m

Constitutive Relation D-E

SETTINGS

Description	Value
Dielectric model	Relative permittivity
Relative permittivity	User defined
Relative permittivity	78

Coordinate System Selection

SETTINGS

Description	Value
Coordinate system	Global coordinate system

Model Input

SETTINGS

Description	Value
Temperature	Common model input

Variables

Name	Expression	Unit	Description	Selection	Details
ec.Qh	ec.Qrh	W/m³	Volumetric loss density, electromagnetic	Domains 1–3	
ec.Jix	ec.sigmaxx*ec.Ex+ec.sigmaxy*ec.Ey+ec.sigmaxz*ec.Ez	A/m²	Conduction current density, x-component	Domains 1–3	
ec.Jiy	ec.sigmayx*ec.Ex+ec.sigmayy*ec.Ey+ec.sigmayz*ec.Ez	A/m²	Conduction current density, y-component	Domains 1–3	
ec.Jiz	ec.sigmazx*ec.Ex+ec.sigmazy*ec.Ey+ec.sigmazz*ec.Ez	A/m²	Conduction current density, z-component	Domains 1–3	
ec.Jdx	0	A/m²	Displacement current density, x-component	Domains 1–3	
ec.Jdy	0	A/m²	Displacement current density, y-component	Domains 1–3	
ec.Jdz	0	A/m²	Displacement current density, z-component	Domains 1–3	
ec.Jex	0	A/m²	External current density, x-component	Domains 1–3	+ operation
ec.Jey	0	A/m²	External current density, y-component	Domains 1–3	+ operation
ec.Jez	0	A/m²	External current density, z-component	Domains 1–3	+ operation

Name	Expression	Unit	Description	Selection	Details
ec.JpX	0	A/m²	Polarization current density, X-component	Domains 1–3	+ operation
ec.JpY	0	A/m²	Polarization current density, Y-component	Domains 1–3	+ operation
ec.JpZ	0	A/m²	Polarization current density, Z-component	Domains 1–3	+ operation
ec.Jx	ec.Jix+ec.Jdx+spatial.F11*ec.JpX*spatial.detInvF+spatial.F21*ec.JpY*spatial.detInvF+spatial.F31*ec.JpZ*spatial.detInvF+ec.Jex	A/m²	Current density, x-component	Domains 1–3	
ec.Jy	ec.Jiy+ec.Jdy+spatial.F12*ec.JpX*spatial.detInvF+spatial.F22*ec.JpY*spatial.detInvF+spatial.F32*ec.JpZ*spatial.detInvF+ec.Jey	A/m²	Current density, y-component	Domains 1–3	
ec.Jz	ec.Jiz+ec.Jdz+spatial.F13*ec.JpX*spatial.detInvF+spatial.F23*ec.JpY*spatial.detInvF+spatial.F33*ec.JpZ*spatial.detInvF+ec.Jez	A/m²	Current density, z-component	Domains 1–3	
ec.normJ	sqrt(realdot(ec.Jx,ec.Jx)+realdot(ec.Jy,ec.Jy)+realdot(ec.Jz,ec.Jz))	A/m²	Current density norm	Domains 1–3	
ec.rhoq	ppr(d(ec.Dx,x)+d(ec.Dy,y)+d(ec.Dz,z))	C/m³	Space charge density	Domains 1–3	
ec.sigmaxx	material.sigma11	S/m	Electrical conductivity, xx-component	Domains 1–3	Meta
ec.sigmayx	material.sigma21	S/m	Electrical conductivity, yx-component	Domains 1–3	Meta

Name	Expression	Unit	Description	Selection	Details
ec.sigmazx	material.sigma31	S/m	Electrical conductivity, zx-component	Domains 1–3	Meta
ec.sigmaxy	material.sigma12	S/m	Electrical conductivity, xy-component	Domains 1–3	Meta
ec.sigmayy	material.sigma22	S/m	Electrical conductivity, yy-component	Domains 1–3	Meta
ec.sigmazy	material.sigma32	S/m	Electrical conductivity, zy-component	Domains 1–3	Meta
ec.sigmaxz	material.sigma13	S/m	Electrical conductivity, xz-component	Domains 1–3	Meta
ec.sigmayz	material.sigma23	S/m	Electrical conductivity, yz-component	Domains 1–3	Meta
ec.sigmazz	material.sigma33	S/m	Electrical conductivity, zz-component	Domains 1–3	Meta
ec.sigma_iso	material.sigma_iso	S/m	Electrical conductivity, isotropic value	Domains 1–3	Meta
ec.epsilonrxx	78	1	Relative permittivity, xx-component	Domains 1–3	
ec.epsilonryx	0	1	Relative permittivity, yx-component	Domains 1–3	
ec.epsilonrzx	0	1	Relative permittivity, zx-component	Domains 1–3	
ec.epsilonrxy	0	1	Relative permittivity, xy-component	Domains 1–3	
ec.epsilonryy	78	1	Relative permittivity, yy-component	Domains 1–3	
ec.epsilonrzy	0	1	Relative permittivity, zy-component	Domains 1–3	

Name	Expression	Unit	Description	Selection	Details
ec.epsilonrxz	0	1	Relative permittivity, xz-component	Domains 1–3	
ec.epsilonryz	0	1	Relative permittivity, yz-component	Domains 1–3	
ec.epsilonrzz	78	1	Relative permittivity, zz-component	Domains 1–3	
ec.epsilonr_iso	78	1	Relative permittivity, isotropic value	Domains 1–3	
ec.Dx	epsilon0_const*ec.I_sxx*ec.Ex+epsilon0_const*ec.I_sxy*ec.Ey+epsilon0_const*ec.I_sxz*ec.Ez+ec.Px+ec.Pex+ec.Phx	C/m^2	Electric displacement field, x-component	Domains 1–3	
ec.Dy	epsilon0_const*ec.I_syx*ec.Ex+epsilon0_const*ec.I_syy*ec.Ey+epsilon0_const*ec.I_syz*ec.Ez+ec.Py+ec.Pey+ec.Phy	C/m^2	Electric displacement field, y-component	Domains 1–3	
ec.Dz	epsilon0_const*ec.I_szx*ec.Ex+epsilon0_const*ec.I_szy*ec.Ey+epsilon0_const*ec.I_szz*ec.Ez+ec.Pz+ec.Pez+ec.Phz	C/m^2	Electric displacement field, z-component	Domains 1–3	
ec.Px	epsilon0_const*(ec.chixx*ec.Ex+ec.chixy*ec.Ey+ec.chixz*ec.Ez)	C/m^2	Polarization, x-component	Domains 1–3	
ec.Py	epsilon0_const*(ec.chiyx*ec.Ex+ec.chiyy*ec.Ey+ec.chiyz*ec.Ez)	C/m^2	Polarization, y-component	Domains 1–3	
ec.Pz	epsilon0_const*(ec.chizx*ec.Ex+ec.chizy*ec.Ey+ec.chizz*ec.Ez)	C/m^2	Polarization, z-component	Domains 1–3	

Name	Expression	Unit	Description	Selection	Details
ec.normD	sqrt(realdot(ec.Dx,ec.Dx)+realdot(ec.Dy,ec.Dy)+realdot(ec.Dz,ec.Dz))	C/m^2	Electric displacement field norm	Domains 1–3	
ec.normP	sqrt(realdot(ec.Px,ec.Px)+realdot(ec.Py,ec.Py)+realdot(ec.Pz,ec.Pz))	C/m^2	Polarization norm	Domains 1–3	
ec.Pex	0	C/m^2	Polarization contribution, x-component	Domains 1–3	+ operation
ec.Pey	0	C/m^2	Polarization contribution, y-component	Domains 1–3	+ operation
ec.Pez	0	C/m^2	Polarization contribution, z-component	Domains 1–3	+ operation
ec.Phx	0	C/m^2	Polarization contribution, x-component	Domains 1–3	+ operation
ec.Phy	0	C/m^2	Polarization contribution, y-component	Domains 1–3	+ operation
ec.Phz	0	C/m^2	Polarization contribution, z-component	Domains 1–3	+ operation
ec.chixx	-1+ec.epsilonrxx	1	Electric susceptibility, xx-component	Domains 1–3	
ec.chiyx	ec.epsilonryx	1	Electric susceptibility, yx-component	Domains 1–3	
ec.chizx	ec.epsilonrzx	1	Electric susceptibility, zx-component	Domains 1–3	
ec.chixy	ec.epsilonrxy	1	Electric susceptibility, xy-component	Domains 1–3	
ec.chiyy	-1+ec.epsilonryy	1	Electric susceptibility, yy-component	Domains 1–3	

Name	Expression	Unit	Description	Selection	Details
ec.chizy	ec.epsilonrzy	1	Electric susceptibility, zy-component	Domains 1–3	
ec.chixz	ec.epsilonrxz	1	Electric susceptibility, xz-component	Domains 1–3	
ec.chiyz	ec.epsilonryz	1	Electric susceptibility, yz-component	Domains 1–3	
ec.chizz	-1+ec.epsilonrzz	1	Electric susceptibility, zz-component	Domains 1–3	
ec.Ex	-Vx	V/m	Electric field, x-component	Domains 1–3	
ec.Ey	-Vy	V/m	Electric field, y-component	Domains 1–3	
ec.Ez	-Vz	V/m	Electric field, z-component	Domains 1–3	
ec.tEx	-VTx	V/m	Tangential electric field, x-component	Boundaries 1–16	
ec.tEy	-VTy	V/m	Tangential electric field, y-component	Boundaries 1–16	
ec.tEz	-VTz	V/m	Tangential electric field, z-component	Boundaries 1–16	
ec.normE	sqrt(realdot(ec.Ex,ec.Ex)+realdot(ec.Ey,ec.Ey)+realdot(ec.Ez,ec.Ez))	V/m	Electric field norm	Domains 1–3	
ec.Qrh	ec.Jx*ec.Ex+ec.Jy*ec.Ey+ec.Jz*ec.Ez	W/m³	Volumetric loss density, electric	Domains 1–3	+ operation
ec.W	ec.We	J/m³	Energy density	Domains 1–3	+ operation
ec.dWe	ec.We	J/m³	Integrand for total electric energy	Domains 1–3	Meta
ec.We	0.5*epsilon0_const*(((ec.I_sxx+ec.chixx)*ec.Ex+(ec.I_sxy+ec.chixy)*ec.Ey+(ec.I_sxz+ec.chixz)*ec.Ez)*ec.Ex+((ec.I_syx+ec.chi	J/m³	Electric energy density	Domains 1–3	

Name	Expression	Unit	Description	Selection	Details
	yx)*ec.Ex+(ec.I_syy+ec.chiyy)*ec.Ey+(ec.I_syz+ec.chiyz)*ec.Ez)*ec.Ey+((ec.I_szx+ec.chizx)*ec.Ex+(ec.I_szy+ec.chizy)*ec.Ey+(ec.I_szz+ec.chizz)*ec.Ez)*ec.Ez)				
ec.rhoqs	ec.dnx*(up(ec.Dx)-down(ec.Dx))+ec.dny*(up(ec.Dy)-down(ec.Dy))+ec.dnz*(up(ec.Dz)-down(ec.Dz))	C/m²	Surface charge density	Boundaries 7, 14	
ec.rhoqs	-ec.dnx*down(ec.Dx)-ec.dny*down(ec.Dy)-ec.dnz*down(ec.Dz)	C/m²	Surface charge density	Boundaries 1–6, 8–13, 15–16	

Shape functions

Name	Shape function	Unit	Description	Shape frame	Selection
V	Lagrange (Quadratic)	V	Electric potential	Spatial	Domains 1–3
V	Lagrange (Quadratic)	V	Electric potential	Material	Domains 1–3
V	Lagrange (Quadratic)	V	Electric potential	Geometry	Domains 1–3
V	Lagrange (Quadratic)	V	Electric potential	Mesh	Domains 1–3

Weak Expressions

Weak expression	Integration order	Integration frame	Selection
(ec.Jx*test(Vx)+ec.Jy*test(Vy)+ec.Jz*test(Vz))*ec.d	4	Spatial	Domains 1–3

2.3.4 Electric Insulation 1

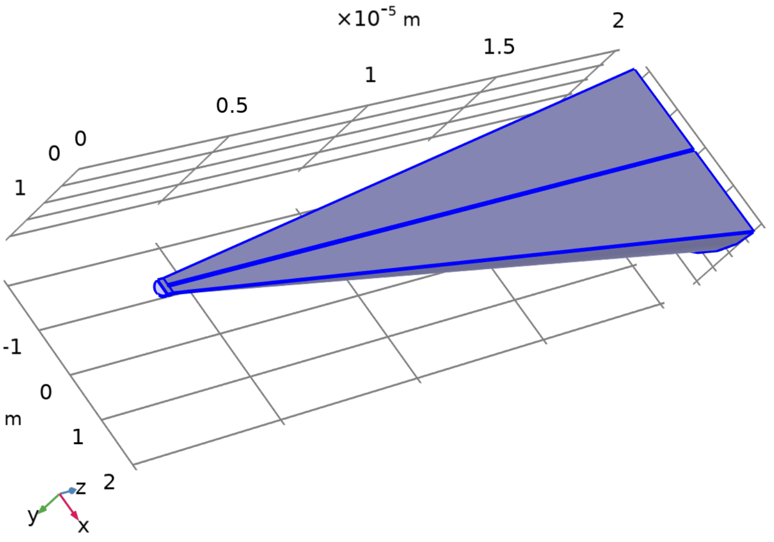

Electric Insulation 1

SELECTION

Geometric entity level	Boundary
Selection	Geometry geom1: Dimension 2: All boundaries

EQUATIONS

$$\mathbf{n}\cdot\mathbf{J}=0$$

Variables

Name	Expression	Unit	Description	Selection	Details
ec.nJ	0	A/m^2	Normal current density	Boundaries 1–2, 4–6, 8–13, 16	+ operation

Shape functions

Name	Shape function	Unit	Description	Shape frame	Selection	Details
V	Lagrange (Quadratic)	V	Electric potential	Spatial	No boundaries	Slit
V	Lagrange (Quadratic)	V	Electric potential	Material	No boundaries	Slit
V	Lagrange (Quadratic)	V	Electric potential	Geometry	No boundaries	Slit
V	Lagrange (Quadratic)	V	Electric potential	Mesh	No boundaries	Slit

2.3.5 Initial Values 1

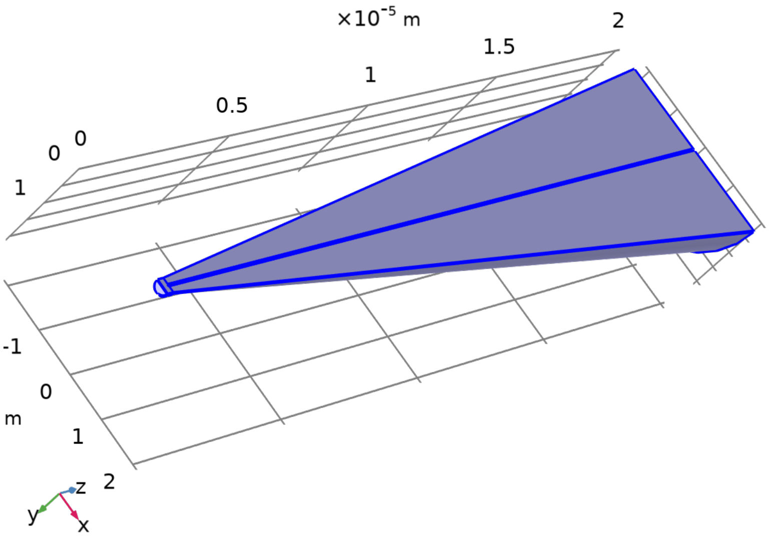

Initial Values 1

SELECTION

Geometric entity level	Domain
Selection	Geometry geom1: Dimension 3: All domains

SETTINGS

Description	Value	Unit
Electric potential	0	V

2.3.6 Electric Potential metals

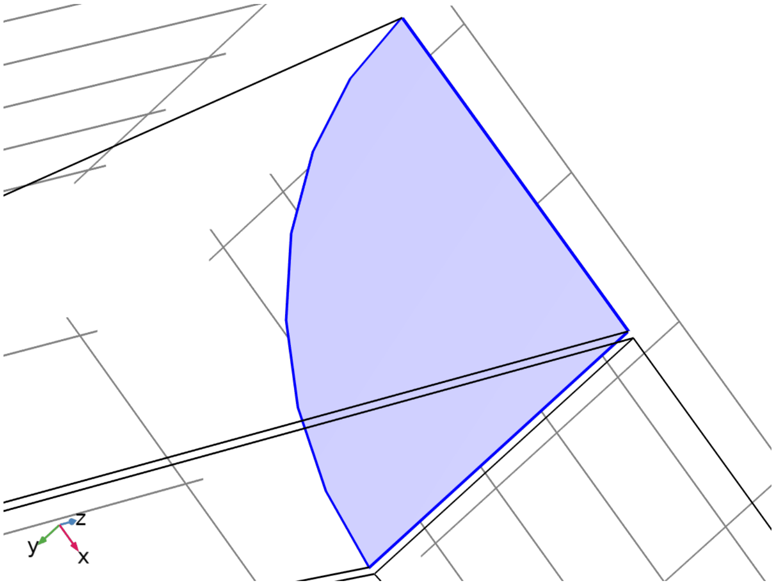

Electric Potential metals

SELECTION

Geometric entity level	Boundary
Selection	Geometry geom1: Dimension 2: Boundary 3

EQUATIONS

$$V = V_0$$

Electric Potential

SETTINGS

Description	Value	Unit
Electric potential	Vmetal	V

Constraint Settings

SETTINGS

Description	Value
Constraint	Pointwise constraints
Apply reaction terms on	All physics (symmetric)
Constraint method	Elemental

Variables

Name	Expression	Unit	Description	Selection	Details
ec.nJ	ec.unx*down(ec.Jx)+ec.uny*down(ec.Jy)+ec.unz*down(ec.Jz)	A/m^2	Normal current density	Boundary 3	+ operation

Name	Expression	Unit	Description	Selection	Details
ec.V0	Vmetal	V	Electric potential	Boundary 3	

Constraints

Constraint	Constraint force	Shape function	Selection	Details
ec.V0-V	test(ec.V0-V)	Lagrange (Quadratic)	Boundary 3	Elemental

2.3.7 Electric Potential empty

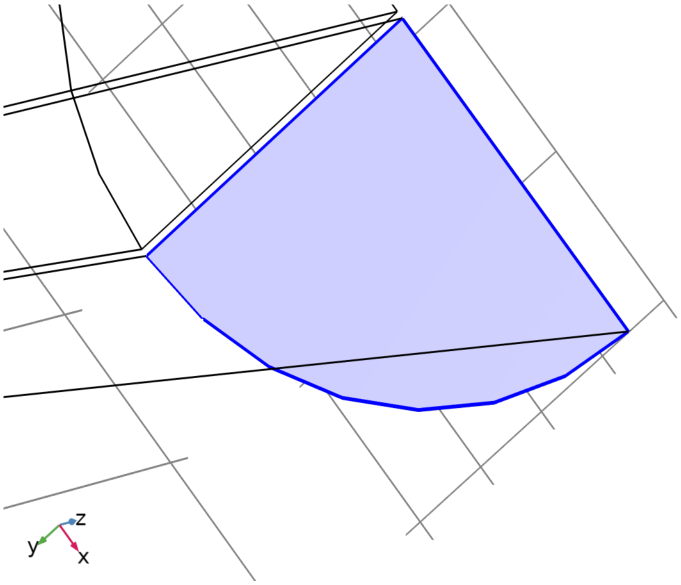

Electric Potential empty

SELECTION

Geometric entity level	Boundary
Selection	Geometry geom1: Dimension 2: Boundary 15

EQUATIONS

$$V = V_0$$

Electric Potential

SETTINGS

Description	Value	Unit
Electric potential	-Vmetal	V

Constraint Settings

SETTINGS

Description	Value
Constraint	Pointwise constraints
Apply reaction terms on	All physics (symmetric)
Constraint method	Elemental

Variables

Name	Expression	Unit	Description	Selection	Details
ec.nJ	ec.unx*down(ec.Jx)+ec.uny*down(ec.Jy)+ec.unz*down(ec.Jz)	A/m^2	Normal current density	Boundary 15	+ operation
ec.V0	-Vmetal	V	Electric potential	Boundary 15	

Constraints

Constraint	Constraint force	Shape function	Selection	Details
ec.V0-V	test(ec.V0-V)	Lagrange (Quadratic)	Boundary 15	Elemental

2.4 TRANSPORT OF DILUTED SPECIES

USED PRODUCTS

COMSOL Multiphysics
Chemical Reaction Engineering Module

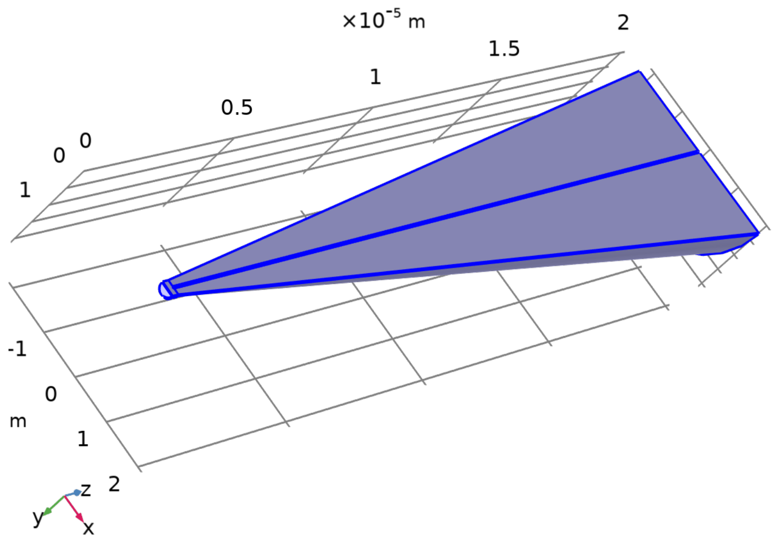

Transport of Diluted Species

SELECTION

Geometric entity level	Domain
Selection	Geometry geom1: Dimension 3: All domains

EQUATIONS

$$\nabla \cdot \mathbf{J}_i = R_i$$
$$\mathbf{J}_i = -D_i \nabla c_i - z_i u_{m,i} F c_i \nabla V$$

2.4.1 Interface Settings

Discretization

SETTINGS

Description	Value
Concentration	Linear

SETTINGS

Description	Value
Equation form	Study controlled

Species Activity

SETTINGS

Description	Value
Species activity	Ideal

Consistent Stabilization

SETTINGS

Description	Value
Streamline diffusion	On
Crosswind diffusion	On
Equation residual	Approximate residual
Crosswind diffusion type	Do Carmo and Galeão

Inconsistent Stabilization

SETTINGS

Description	Value
Isotropic diffusion	Off

Advanced Settings

SETTINGS

Description	Value
Convective term	Nonconservative form

Transport Mechanisms

SETTINGS

Description	Value
Convection	Off
Migration in electric field	On
Mass transfer in porous media	Off

2.4.2 Variables

Name	Expression	Unit	Description	Selection	Details
tds.d	1	1	Out-of-plane geometry extension	Global	
tds.f_cAu	1	1	Activity coefficient	Domains 1–3	
tds.f_cCu	1	1	Activity coefficient	Domains 1–3	
tds.f_cH	1	1	Activity coefficient	Domains 1–3	
tds.f_cSO4	1	1	Activity coefficient	Domains 1–3	
tds.nx	nx	1	Normal vector, x-component	Boundaries 7, 14	
tds.ny	ny	1	Normal vector, y-component	Boundaries 7, 14	
tds.nz	nz	1	Normal vector, z-component	Boundaries 7, 14	
tds.nx	dnx	1	Normal vector, x-component	Boundaries 1–6, 8–13, 15–16	
tds.ny	dny	1	Normal vector, y-component	Boundaries 1–6, 8–13, 15–16	
tds.nz	dnz	1	Normal vector, z-component	Boundaries 1–6, 8–13, 15–16	
tds.nX	nX	1	Normal vector, X-component	Boundaries 7, 14	
tds.nY	nY	1	Normal vector, Y-component	Boundaries 7, 14	
tds.nZ	nZ	1	Normal vector, Z-component	Boundaries 7, 14	
tds.nX	dnX	1	Normal vector, X-component	Boundaries 1–6, 8–13, 15–16	

Name	Expression	Unit	Description	Selection	Details
tds.nY	dnY	1	Normal vector, Y-component	Boundaries 1–6, 8–13, 15–16	
tds.nZ	dnZ	1	Normal vector, Z-component	Boundaries 1–6, 8–13, 15–16	
tds.nXg	nXg	1	Normal vector, Xg-component	Boundaries 7, 14	
tds.nYg	nYg	1	Normal vector, Yg-component	Boundaries 7, 14	
tds.nZg	nZg	1	Normal vector, Zg-component	Boundaries 7, 14	
tds.nXg	dnXg	1	Normal vector, Xg-component	Boundaries 1–6, 8–13, 15–16	
tds.nYg	dnYg	1	Normal vector, Yg-component	Boundaries 1–6, 8–13, 15–16	
tds.nZg	dnZg	1	Normal vector, Zg-component	Boundaries 1–6, 8–13, 15–16	
tds.nxmesh	nxmesh	1	Normal vector (mesh), x-component	Boundaries 7, 14	
tds.nymesh	nymesh	1	Normal vector (mesh), y-component	Boundaries 7, 14	
tds.nzmesh	nzmesh	1	Normal vector (mesh), z-component	Boundaries 7, 14	
tds.nxmesh	dnxmesh	1	Normal vector (mesh), x-component	Boundaries 1–6, 8–13, 15–16	
tds.nymesh	dnymesh	1	Normal vector (mesh), y-component	Boundaries 1–6, 8–13, 15–16	
tds.nzmesh	dnzmesh	1	Normal vector (mesh), z-component	Boundaries 1–6, 8–13, 15–16	
tds.nxc	nxc/tds.ncLen	1	Normal vector, x-component	Boundaries 1–16	

Name	Expression	Unit	Description	Selection	Details
tds.nyc	nyc/tds.ncLen	1	Normal vector, y-component	Boundaries 1–16	
tds.nzc	nzc/tds.ncLen	1	Normal vector, z-component	Boundaries 1–16	
tds.ncLen	sqrt(nxc^2+nyc^2+nzc^2+eps)	1	Help variable	Boundaries 1–16	
tds.R_cAu	0	mol/(m³·s)	Total rate expression	Domains 1–3	+ operation
tds.cP_cAu	0	mol/kg	Concentration species adsorbed to the solid	Domains 1–3	+ operation
tds.cP_cAu	0	mol/kg	Concentration species adsorbed to the solid	Boundaries 1–16	+ operation
tds.KP_cAu	0	m³/kg	Adsorption isotherm, first concentration derivative	Domains 1–3	+ operation
tds.KP_cAu	0	m³/kg	Adsorption isotherm, first concentration derivative	Boundaries 1–16	+ operation
tds.Rads_cAu	0	mol/(m³·s)	Total adsorption rate	Domains 1–3	+ operation
tds.DiT_cAu	0	m²/s	Turbulent diffusivity	Domains 1–3	
tds.cVar_cAu	cAu	mol/m³	Species	Boundaries 1–16	
tds.cVar_cAu	cAu	mol/m³	Species	Edges 1–29	
tds.cVar_cAu	cAu	mol/m³	Species	Points 1–17	
tds.R_cCu	0	mol/(m³·s)	Total rate expression	Domains 1–3	+ operation
tds.cP_cCu	0	mol/kg	Concentration species adsorbed to the solid	Domains 1–3	+ operation
tds.cP_cCu	0	mol/kg	Concentration species adsorbed to the solid	Boundaries 1–16	+ operation
tds.KP_cCu	0	m³/kg	Adsorption isotherm, first concentration derivative	Domains 1–3	+ operation

Name	Expression	Unit	Description	Selection	Details
tds.KP_cCu	0	m^3/kg	Adsorption isotherm, first concentration derivative	Boundaries 1–16	+ operation
tds.Rads_cCu	0	$mol/(m^3 \cdot s)$	Total adsorption rate	Domains 1–3	+ operation
tds.DiT_cCu	0	m^2/s	Turbulent diffusivity	Domains 1–3	
tds.cVar_cCu	cCu	mol/m^3	Species	Boundaries 1–16	
tds.cVar_cCu	cCu	mol/m^3	Species	Edges 1–29	
tds.cVar_cCu	cCu	mol/m^3	Species	Points 1–17	
tds.R_cH	0	$mol/(m^3 \cdot s)$	Total rate expression	Domains 1–3	+ operation
tds.cP_cH	0	mol/kg	Concentration species adsorbed to the solid	Domains 1–3	+ operation
tds.cP_cH	0	mol/kg	Concentration species adsorbed to the solid	Boundaries 1–16	+ operation
tds.KP_cH	0	m^3/kg	Adsorption isotherm, first concentration derivative	Domains 1–3	+ operation
tds.KP_cH	0	m^3/kg	Adsorption isotherm, first concentration derivative	Boundaries 1–16	+ operation
tds.Rads_cH	0	$mol/(m^3 \cdot s)$	Total adsorption rate	Domains 1–3	+ operation
tds.DiT_cH	0	m^2/s	Turbulent diffusivity	Domains 1–3	
tds.cVar_cH	cH	mol/m^3	Species	Boundaries 1–16	
tds.cVar_cH	cH	mol/m^3	Species	Edges 1–29	
tds.cVar_cH	cH	mol/m^3	Species	Points 1–17	
tds.R_cSO4	0	$mol/(m^3 \cdot s)$	Total rate expression	Domains 1–3	+ operation
tds.cP_cSO4	0	mol/kg	Concentration species adsorbed to the solid	Domains 1–3	+ operation

Name	Expression	Unit	Description	Selection	Details
tds.cP_cSO4	0	mol/kg	Concentration species adsorbed to the solid	Boundaries 1–16	+ operation
tds.KP_cSO4	0	m^3/kg	Adsorption isotherm, first concentration derivative	Domains 1–3	+ operation
tds.KP_cSO4	0	m^3/kg	Adsorption isotherm, first concentration derivative	Boundaries 1–16	+ operation
tds.Rads_cSO4	0	mol/(m^3·s)	Total adsorption rate	Domains 1–3	+ operation
tds.DiT_cSO4	0	m^2/s	Turbulent diffusivity	Domains 1–3	
tds.cVar_cSO4	cSO4	mol/m^3	Species	Boundaries 1–16	
tds.cVar_cSO4	cSO4	mol/m^3	Species	Edges 1–29	
tds.cVar_cSO4	cSO4	mol/m^3	Species	Points 1–17	
tds.poro	1	1	Porosity	Domains 1–3	
tds.theta_g	0	1	Gas volume fraction	Domains 1–3	
tds.theta_l	1	1	Liquid volume fraction	Domains 1–3	
tds.theta	tds.poro	1	Mobile fluid volume fraction	Domains 1–3	

2.4.3 Species Charges

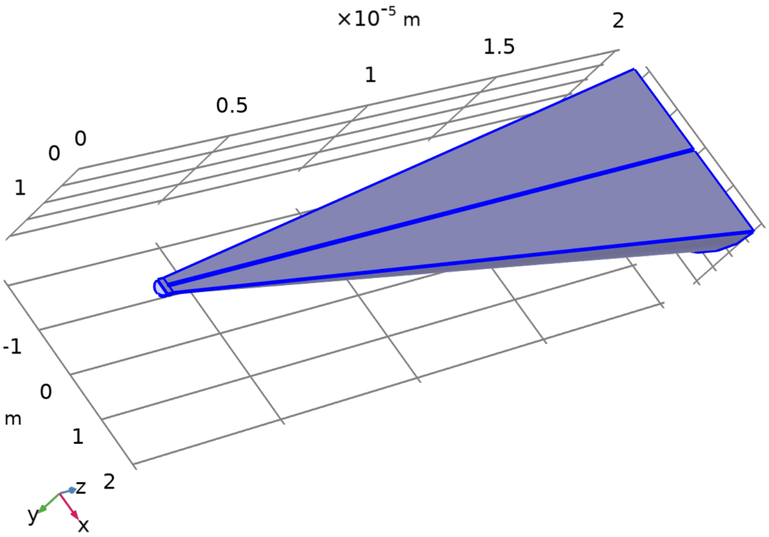

Species Charges

SELECTION

Geometric entity level	Domain
Selection	Geometry geom1: Dimension 3: All domains

Charge

SETTINGS

**Description**	**Value**
Charge number	{-1, 2, 1, -2}

Model Input

SETTINGS

**Description**	**Value**
Temperature	Common model input

Variables

**Name**	**Expression**	**Unit**	**Description**	**Selection**
tds.z_cAu	-1	1	Charge number	Domains 1–3
tds.z_cCu	2	1	Charge number	Domains 1–3
tds.z_cH	1	1	Charge number	Domains 1–3
tds.z_cSO4	-2	1	Charge number	Domains 1–3

2.4.4 Transport Properties 1

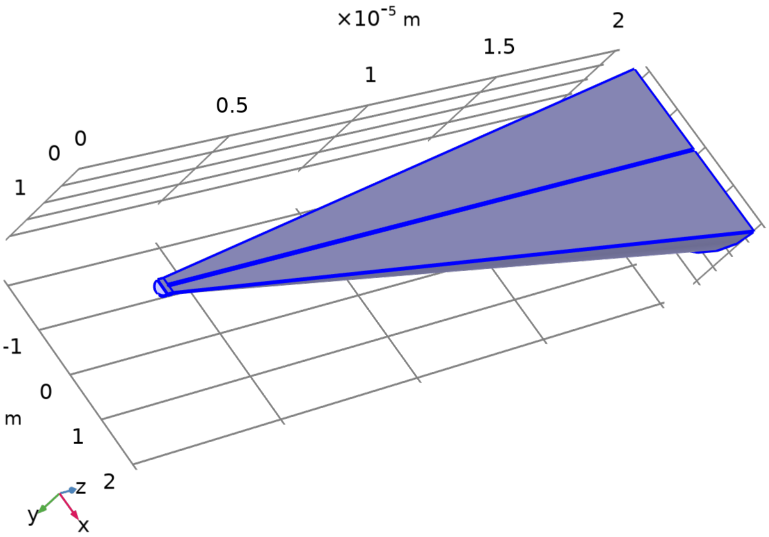

Transport Properties 1

SELECTION

Geometric entity level	Domain
Selection	Geometry geom1: Dimension 3: All domains

EQUATIONS

$$\nabla \cdot \mathbf{J}_i = R_i$$

$$\mathbf{J}_i = -D_i \nabla c_i - z_i u_{m,i} F c_i \nabla V$$

Diffusion

SETTINGS

**Description**	**Value**	**Unit**
Source	Chemistry	
Diffusion coefficient	User defined	
Diffusion coefficient	DAu	m^2/s
Diffusion coefficient	User defined	
Diffusion coefficient	DCu	m^2/s
Diffusion coefficient	User defined	
Diffusion coefficient	DH	m^2/s
Diffusion coefficient	User defined	
Diffusion coefficient	DSO4	m^2/s

Migration in Electric Field

SETTINGS

Description	Value
Electric potential	Electric potential (ec)
Mobility	Nernst - Einstein relation

Coordinate System Selection

SETTINGS

Description	Value
Coordinate system	Global coordinate system

Model Input

SETTINGS

Description	Value
Temperature	Common model input

Variables

Name	Expression	Unit	Description	Selection	Details
domflux.cAux	(tds.dflux_cAux+tds.mflux_cAux)*tds.d	mol/(m²·s)	Domain flux, x-component	Domains 1–3	
domflux.cAuy	(tds.dflux_cAuy+tds.mflux_cAuy)*tds.d	mol/(m²·s)	Domain flux, y-component	Domains 1–3	
domflux.cAuz	(tds.dflux_cAuz+tds.mflux_cAuz)*tds.d	mol/(m²·s)	Domain flux, z-component	Domains 1–3	
domflux.cCux	(tds.dflux_cCux+tds.mflux_cCux)*tds.d	mol/(m²·s)	Domain flux, x-component	Domains 1–3	
domflux.cCuy	(tds.dflux_cCuy+tds.mflux_cCuy)*tds.d	mol/(m²·s)	Domain flux, y-component	Domains 1–3	
domflux.cCuz	(tds.dflux_cCuz+tds.mflux_cCuz)*tds.d	mol/(m²·s)	Domain flux, z-component	Domains 1–3	
domflux.cHx	(tds.dflux_cHx+tds.mflux_cHx)*tds.d	mol/(m²·s)	Domain flux, x-component	Domains 1–3	
domflux.cHy	(tds.dflux_cHy+tds.mflux_cHy)*tds.d	mol/(m²·s)	Domain flux, y-component	Domains 1–3	

Name	Expression	Unit	Description	Selection	Details
domflux.cHz	(tds.dflux_cHz+tds.mflux_cHz)*tds.d	mol/(m²·s)	Domain flux, z-component	Domains 1–3	
domflux.cSO4x	(tds.dflux_cSO4x+tds.mflux_cSO4x)*tds.d	mol/(m²·s)	Domain flux, x-component	Domains 1–3	
domflux.cSO4y	(tds.dflux_cSO4y+tds.mflux_cSO4y)*tds.d	mol/(m²·s)	Domain flux, y-component	Domains 1–3	
domflux.cSO4z	(tds.dflux_cSO4z+tds.mflux_cSO4z)*tds.d	mol/(m²·s)	Domain flux, z-component	Domains 1–3	
tds.ndflux_cAu	tds.dflux_cAux*tds.nxc+tds.dflux_cAuy*tds.nyc+tds.dflux_cAuz*tds.nzc	mol/(m²·s)	Normal diffusive flux	Boundaries 1–16	
tds.nmflux_cAu	tds.mflux_cAux*tds.nxc+tds.mflux_cAuy*tds.nyc+tds.mflux_cAuz*tds.nzc	mol/(m²·s)	Normal electrophoretic flux	Boundaries 1–16	
tds.ntflux_cAu	tds.bndFlux_cAu	mol/(m²·s)	Normal total flux	Boundaries 1–16	
tds.ndflux_cCu	tds.dflux_cCux*tds.nxc+tds.dflux_cCuy*tds.nyc+tds.dflux_cCuz*tds.nzc	mol/(m²·s)	Normal diffusive flux	Boundaries 1–16	
tds.nmflux_cCu	tds.mflux_cCux*tds.nxc+tds.mflux_cCuy*tds.nyc+tds.mflux_cCuz*tds.nzc	mol/(m²·s)	Normal electrophoretic flux	Boundaries 1–16	
tds.ntflux_cCu	tds.bndFlux_cCu	mol/(m²·s)	Normal total flux	Boundaries 1–16	
tds.ndflux_cH	tds.dflux_cHx*tds.nxc+tds.dflux_cHy*tds.nyc+tds.dflux_cHz*tds.nzc	mol/(m²·s)	Normal diffusive flux	Boundaries 1–16	
tds.nmflux_cH	tds.mflux_cHx*tds.nxc+tds.mflux_cHy*tds.nyc+tds.mflux_cHz*tds.nzc	mol/(m²·s)	Normal electrophoretic flux	Boundaries 1–16	
tds.ntflux_cH	tds.bndFlux_cH	mol/(m²·s)	Normal total flux	Boundaries 1–16	
tds.ndflux_cSO4	tds.dflux_cSO4x*tds.nxc+tds.dflux_cS	mol/(m²·s)	Normal diffusive flux	Boundaries 1–16	

Name	Expression	Unit	Description	Selection	Details
	O4y*tds.nyc+tds.dflux_cSO4z*tds.nzc				
tds.nmflux_cSO4	tds.mflux_cSO4x*tds.nxc+tds.mflux_cSO4y*tds.nyc+tds.mflux_cSO4z*tds.nzc	mol/(m²·s)	Normal electrophoretic flux	Boundaries 1–16	
tds.ntflux_cSO4	tds.bndFlux_cSO4	mol/(m²·s)	Normal total flux	Boundaries 1–16	
tds.bndFlux_cAu	0.5*(uflux_spatial(cAu)-dflux_spatial(cAu))/tds.d	mol/(m²·s)	Boundary flux	Boundaries 7, 14	Meta
tds.bndFlux_cAu	-dflux_spatial(cAu)/tds.d	mol/(m²·s)	Boundary flux	Boundaries 1–6, 8–13, 15–16	Meta
tds.bndFlux_cCu	0.5*(uflux_spatial(cCu)-dflux_spatial(cCu))/tds.d	mol/(m²·s)	Boundary flux	Boundaries 7, 14	Meta
tds.bndFlux_cCu	-dflux_spatial(cCu)/tds.d	mol/(m²·s)	Boundary flux	Boundaries 1–6, 8–13, 15–16	Meta
tds.bndFlux_cH	0.5*(uflux_spatial(cH)-dflux_spatial(cH))/tds.d	mol/(m²·s)	Boundary flux	Boundaries 7, 14	Meta
tds.bndFlux_cH	-dflux_spatial(cH)/tds.d	mol/(m²·s)	Boundary flux	Boundaries 1–6, 8–13, 15–16	Meta
tds.bndFlux_cSO4	0.5*(uflux_spatial(cSO4)-dflux_spatial(cSO4))/tds.d	mol/(m²·s)	Boundary flux	Boundaries 7, 14	Meta
tds.bndFlux_cSO4	-dflux_spatial(cSO4)/tds.d	mol/(m²·s)	Boundary flux	Boundaries 1–6, 8–13, 15–16	Meta
tds.DF_cAuxx	model.input.Dchem_cAu11	m²/s	Fluid diffusion coefficient, xx-component	Domains 1–3	Meta
tds.DF_cAuyx	model.input.Dchem_cAu21	m²/s	Fluid diffusion coefficient, yx-component	Domains 1–3	Meta

Name	Expression	Unit	Description	Selection	Details
tds.DF_cAuzx	model.input.Dchem_cAu31	m^2/s	Fluid diffusion coefficient, zx-component	Domains 1–3	Meta
tds.DF_cAuxy	model.input.Dchem_cAu12	m^2/s	Fluid diffusion coefficient, xy-component	Domains 1–3	Meta
tds.DF_cAuyy	model.input.Dchem_cAu22	m^2/s	Fluid diffusion coefficient, yy-component	Domains 1–3	Meta
tds.DF_cAuzy	model.input.Dchem_cAu32	m^2/s	Fluid diffusion coefficient, zy-component	Domains 1–3	Meta
tds.DF_cAuxz	model.input.Dchem_cAu13	m^2/s	Fluid diffusion coefficient, xz-component	Domains 1–3	Meta
tds.DF_cAuyz	model.input.Dchem_cAu23	m^2/s	Fluid diffusion coefficient, yz-component	Domains 1–3	Meta
tds.DF_cAuzz	model.input.Dchem_cAu33	m^2/s	Fluid diffusion coefficient, zz-component	Domains 1–3	Meta
tds.D_cAuxx	tds.DF_cAuxx+tds.DiT_cAu	m^2/s	Diffusion coefficient, xx-component	Domains 1–3	+ operation
tds.D_cAuyx	tds.DF_cAuyx	m^2/s	Diffusion coefficient, yx-component	Domains 1–3	+ operation
tds.D_cAuzx	tds.DF_cAuzx	m^2/s	Diffusion coefficient, zx-component	Domains 1–3	+ operation
tds.D_cAuxy	tds.DF_cAuxy	m^2/s	Diffusion coefficient, xy-component	Domains 1–3	+ operation
tds.D_cAuyy	tds.DF_cAuyy+tds.DiT_cAu	m^2/s	Diffusion coefficient, yy-component	Domains 1–3	+ operation
tds.D_cAuzy	tds.DF_cAuzy	m^2/s	Diffusion coefficient, zy-component	Domains 1–3	+ operation
tds.D_cAuxz	tds.DF_cAuxz	m^2/s	Diffusion coefficient, xz-component	Domains 1–3	+ operation

Name	Expression	Unit	Description	Selection	Details
tds.D_cAuyz	tds.DF_cAuyz	m²/s	Diffusion coefficient, yz-component	Domains 1–3	+ operation
tds.D_cAuzz	tds.DF_cAuzz+tds.DiT_cAu	m²/s	Diffusion coefficient, zz-component	Domains 1–3	+ operation
tds.DF_cCuxx	model.input.Dchem_cCu11	m²/s	Fluid diffusion coefficient, xx-component	Domains 1–3	Meta
tds.DF_cCuyx	model.input.Dchem_cCu21	m²/s	Fluid diffusion coefficient, yx-component	Domains 1–3	Meta
tds.DF_cCuzx	model.input.Dchem_cCu31	m²/s	Fluid diffusion coefficient, zx-component	Domains 1–3	Meta
tds.DF_cCuxy	model.input.Dchem_cCu12	m²/s	Fluid diffusion coefficient, xy-component	Domains 1–3	Meta
tds.DF_cCuyy	model.input.Dchem_cCu22	m²/s	Fluid diffusion coefficient, yy-component	Domains 1–3	Meta
tds.DF_cCuzy	model.input.Dchem_cCu32	m²/s	Fluid diffusion coefficient, zy-component	Domains 1–3	Meta
tds.DF_cCuxz	model.input.Dchem_cCu13	m²/s	Fluid diffusion coefficient, xz-component	Domains 1–3	Meta
tds.DF_cCuyz	model.input.Dchem_cCu23	m²/s	Fluid diffusion coefficient, yz-component	Domains 1–3	Meta
tds.DF_cCuzz	model.input.Dchem_cCu33	m²/s	Fluid diffusion coefficient, zz-component	Domains 1–3	Meta
tds.D_cCuxx	tds.DF_cCuxx+tds.DiT_cCu	m²/s	Diffusion coefficient, xx-component	Domains 1–3	+ operation
tds.D_cCuyx	tds.DF_cCuyx	m²/s	Diffusion coefficient, yx-component	Domains 1–3	+ operation
tds.D_cCuzx	tds.DF_cCuzx	m²/s	Diffusion coefficient, zx-component	Domains 1–3	+ operation

Name	Expression	Unit	Description	Selection	Details
tds.D_cCuxy	tds.DF_cCuxy	m^2/s	Diffusion coefficient, xy-component	Domains 1–3	+ operation
tds.D_cCuyy	tds.DF_cCuyy+tds.DiT_cCu	m^2/s	Diffusion coefficient, yy-component	Domains 1–3	+ operation
tds.D_cCuzy	tds.DF_cCuzy	m^2/s	Diffusion coefficient, zy-component	Domains 1–3	+ operation
tds.D_cCuxz	tds.DF_cCuxz	m^2/s	Diffusion coefficient, xz-component	Domains 1–3	+ operation
tds.D_cCuyz	tds.DF_cCuyz	m^2/s	Diffusion coefficient, yz-component	Domains 1–3	+ operation
tds.D_cCuzz	tds.DF_cCuzz+tds.DiT_cCu	m^2/s	Diffusion coefficient, zz-component	Domains 1–3	+ operation
tds.DF_cHxx	model.input.Dchem_cH11	m^2/s	Fluid diffusion coefficient, xx-component	Domains 1–3	Meta
tds.DF_cHyx	model.input.Dchem_cH21	m^2/s	Fluid diffusion coefficient, yx-component	Domains 1–3	Meta
tds.DF_cHzx	model.input.Dchem_cH31	m^2/s	Fluid diffusion coefficient, zx-component	Domains 1–3	Meta
tds.DF_cHxy	model.input.Dchem_cH12	m^2/s	Fluid diffusion coefficient, xy-component	Domains 1–3	Meta
tds.DF_cHyy	model.input.Dchem_cH22	m^2/s	Fluid diffusion coefficient, yy-component	Domains 1–3	Meta
tds.DF_cHzy	model.input.Dchem_cH32	m^2/s	Fluid diffusion coefficient, zy-component	Domains 1–3	Meta
tds.DF_cHxz	model.input.Dchem_cH13	m^2/s	Fluid diffusion coefficient, xz-component	Domains 1–3	Meta
tds.DF_cHyz	model.input.Dchem_cH23	m^2/s	Fluid diffusion coefficient, yz-component	Domains 1–3	Meta

Name	Expression	Unit	Description	Selection	Details
tds.DF_cHzz	model.input.Dchem_cH33	m²/s	Fluid diffusion coefficient, zz-component	Domains 1–3	Meta
tds.D_cHxx	tds.DF_cHxx+tds.DiT_cH	m²/s	Diffusion coefficient, xx-component	Domains 1–3	+ operation
tds.D_cHyx	tds.DF_cHyx	m²/s	Diffusion coefficient, yx-component	Domains 1–3	+ operation
tds.D_cHzx	tds.DF_cHzx	m²/s	Diffusion coefficient, zx-component	Domains 1–3	+ operation
tds.D_cHxy	tds.DF_cHxy	m²/s	Diffusion coefficient, xy-component	Domains 1–3	+ operation
tds.D_cHyy	tds.DF_cHyy+tds.DiT_cH	m²/s	Diffusion coefficient, yy-component	Domains 1–3	+ operation
tds.D_cHzy	tds.DF_cHzy	m²/s	Diffusion coefficient, zy-component	Domains 1–3	+ operation
tds.D_cHxz	tds.DF_cHxz	m²/s	Diffusion coefficient, xz-component	Domains 1–3	+ operation
tds.D_cHyz	tds.DF_cHyz	m²/s	Diffusion coefficient, yz-component	Domains 1–3	+ operation
tds.D_cHzz	tds.DF_cHzz+tds.DiT_cH	m²/s	Diffusion coefficient, zz-component	Domains 1–3	+ operation
tds.DF_cSO4xx	model.input.Dchem_cSO411	m²/s	Fluid diffusion coefficient, xx-component	Domains 1–3	Meta
tds.DF_cSO4yx	model.input.Dchem_cSO421	m²/s	Fluid diffusion coefficient, yx-component	Domains 1–3	Meta
tds.DF_cSO4zx	model.input.Dchem_cSO431	m²/s	Fluid diffusion coefficient, zx-component	Domains 1–3	Meta
tds.DF_cSO4xy	model.input.Dchem_cSO412	m²/s	Fluid diffusion coefficient, xy-component	Domains 1–3	Meta

Name	Expression	Unit	Description	Selection	Details
tds.DF_cSO4yy	model.input.Dchem_cSO422	m^2/s	Fluid diffusion coefficient, yy-component	Domains 1–3	Meta
tds.DF_cSO4zy	model.input.Dchem_cSO432	m^2/s	Fluid diffusion coefficient, zy-component	Domains 1–3	Meta
tds.DF_cSO4xz	model.input.Dchem_cSO413	m^2/s	Fluid diffusion coefficient, xz-component	Domains 1–3	Meta
tds.DF_cSO4yz	model.input.Dchem_cSO423	m^2/s	Fluid diffusion coefficient, yz-component	Domains 1–3	Meta
tds.DF_cSO4zz	model.input.Dchem_cSO433	m^2/s	Fluid diffusion coefficient, zz-component	Domains 1–3	Meta
tds.D_cSO4xx	tds.DF_cSO4xx+tds.DiT_cSO4	m^2/s	Diffusion coefficient, xx-component	Domains 1–3	+ operation
tds.D_cSO4yx	tds.DF_cSO4yx	m^2/s	Diffusion coefficient, yx-component	Domains 1–3	+ operation
tds.D_cSO4zx	tds.DF_cSO4zx	m^2/s	Diffusion coefficient, zx-component	Domains 1–3	+ operation
tds.D_cSO4xy	tds.DF_cSO4xy	m^2/s	Diffusion coefficient, xy-component	Domains 1–3	+ operation
tds.D_cSO4yy	tds.DF_cSO4yy+tds.DiT_cSO4	m^2/s	Diffusion coefficient, yy-component	Domains 1–3	+ operation
tds.D_cSO4zy	tds.DF_cSO4zy	m^2/s	Diffusion coefficient, zy-component	Domains 1–3	+ operation
tds.D_cSO4xz	tds.DF_cSO4xz	m^2/s	Diffusion coefficient, xz-component	Domains 1–3	+ operation
tds.D_cSO4yz	tds.DF_cSO4yz	m^2/s	Diffusion coefficient, yz-component	Domains 1–3	+ operation
tds.D_cSO4zz	tds.DF_cSO4zz+tds.DiT_cSO4	m^2/s	Diffusion coefficient, zz-component	Domains 1–3	+ operation

Name	Expression	Unit	Description	Selection	Details
tds.Dav_cAu	(tds.D_cAuxx+tds.D_cAuyy+tds.D_cAuzz)/3	m²/s	Average diffusion coefficient	Domains 1–3	
tds.Dav_cCu	(tds.D_cCuxx+tds.D_cCuyy+tds.D_cCuzz)/3	m²/s	Average diffusion coefficient	Domains 1–3	
tds.Dav_cH	(tds.D_cHxx+tds.D_cHyy+tds.D_cHzz)/3	m²/s	Average diffusion coefficient	Domains 1–3	
tds.Dav_cSO4	(tds.D_cSO4xx+tds.D_cSO4yy+tds.D_cSO4zz)/3	m²/s	Average diffusion coefficient	Domains 1–3	
tds.tflux_cAux	tds.dflux_cAux+tds.mflux_cAux	mol/(m²·s)	Total flux, x-component	Domains 1–3	+ operation
tds.tflux_cAuy	tds.dflux_cAuy+tds.mflux_cAuy	mol/(m²·s)	Total flux, y-component	Domains 1–3	+ operation
tds.tflux_cAuz	tds.dflux_cAuz+tds.mflux_cAuz	mol/(m²·s)	Total flux, z-component	Domains 1–3	+ operation
tds.dfluxMag_cAu	sqrt(tds.dflux_cAux^2+tds.dflux_cAuy^2+tds.dflux_cAuz^2)	mol/(m²·s)	Diffusive flux magnitude	Domains 1–3	
tds.tfluxMag_cAu	sqrt(tds.tflux_cAux^2+tds.tflux_cAuy^2+tds.tflux_cAuz^2)	mol/(m²·s)	Total flux magnitude	Domains 1–3	
tds.dpflux_cAux	0	mol/(m²·s)	Dispersive flux, x-component	Domains 1–3	
tds.dpflux_cAuy	0	mol/(m²·s)	Dispersive flux, y-component	Domains 1–3	
tds.dpflux_cAuz	0	mol/(m²·s)	Dispersive flux, z-component	Domains 1–3	
tds.mflux_cAux	tds.z_cAu*F_const*cAu*(-tds.um_cAuxx*d(tds.V,x)-tds.um_cAuxy*d(tds.V,y)-tds.um_cAuxz*d(tds.V,z))	mol/(m²·s)	Electrophoretic flux, x-component	Domains 1–3	
tds.mflux_cAuy	tds.z_cAu*F_const*cAu*(-tds.um_cAuyx*d(td	mol/(m²·s)	Electrophoretic flux, y-component	Domains 1–3	

Name	Expression	Unit	Description	Selection	Details
	s.V,x)-tds.um_cAuyy*d(tds.V,y)-tds.um_cAuyz*d(tds.V,z))				
tds.mflux_cAuz	tds.z_cAu*F_const*cAu*(-tds.um_cAuzx*d(tds.V,x)-tds.um_cAuzy*d(tds.V,y)-tds.um_cAuzz*d(tds.V,z))	mol/(m²·s)	Electrophoretic flux, z-component	Domains 1–3	
tds.mfluxMag_cAu	sqrt(tds.mflux_cAux^2+tds.mflux_cAuy^2+tds.mflux_cAuz^2)	mol/(m²·s)	Electrophoretic flux magnitude	Domains 1–3	
tds.tflux_cCux	tds.dflux_cCux+tds.mflux_cCux	mol/(m²·s)	Total flux, x-component	Domains 1–3	+ operation
tds.tflux_cCuy	tds.dflux_cCuy+tds.mflux_cCuy	mol/(m²·s)	Total flux, y-component	Domains 1–3	+ operation
tds.tflux_cCuz	tds.dflux_cCuz+tds.mflux_cCuz	mol/(m²·s)	Total flux, z-component	Domains 1–3	+ operation
tds.dfluxMag_cCu	sqrt(tds.dflux_cCux^2+tds.dflux_cCuy^2+tds.dflux_cCuz^2)	mol/(m²·s)	Diffusive flux magnitude	Domains 1–3	
tds.tfluxMag_cCu	sqrt(tds.tflux_cCux^2+tds.tflux_cCuy^2+tds.tflux_cCuz^2)	mol/(m²·s)	Total flux magnitude	Domains 1–3	
tds.dpflux_cCux	0	mol/(m²·s)	Dispersive flux, x-component	Domains 1–3	
tds.dpflux_cCuy	0	mol/(m²·s)	Dispersive flux, y-component	Domains 1–3	
tds.dpflux_cCuz	0	mol/(m²·s)	Dispersive flux, z-component	Domains 1–3	
tds.mflux_cCux	tds.z_cCu*F_const*cCu*(-tds.um_cCuxx*d(tds.V,x)-tds.um_cCuxy*d(tds.V,y)-	mol/(m²·s)	Electrophoretic flux, x-component	Domains 1–3	

Name	Expression	Unit	Description	Selection	Details
	tds.um_cCuxz*d(tds.V,z))				
tds.mflux_cCuy	tds.z_cCu*F_const*cCu*(-tds.um_cCuyx*d(tds.V,x)-tds.um_cCuyy*d(tds.V,y)-tds.um_cCuyz*d(tds.V,z))	mol/(m²·s)	Electrophoretic flux, y-component	Domains 1–3	
tds.mflux_cCuz	tds.z_cCu*F_const*cCu*(-tds.um_cCuzx*d(tds.V,x)-tds.um_cCuzy*d(tds.V,y)-tds.um_cCuzz*d(tds.V,z))	mol/(m²·s)	Electrophoretic flux, z-component	Domains 1–3	
tds.mfluxMag_cCu	sqrt(tds.mflux_cCux^2+tds.mflux_cCuy^2+tds.mflux_cCuz^2)	mol/(m²·s)	Electrophoretic flux magnitude	Domains 1–3	
tds.tflux_cHx	tds.dflux_cHx+tds.mflux_cHx	mol/(m²·s)	Total flux, x-component	Domains 1–3	+ operation
tds.tflux_cHy	tds.dflux_cHy+tds.mflux_cHy	mol/(m²·s)	Total flux, y-component	Domains 1–3	+ operation
tds.tflux_cHz	tds.dflux_cHz+tds.mflux_cHz	mol/(m²·s)	Total flux, z-component	Domains 1–3	+ operation
tds.dfluxMag_cH	sqrt(tds.dflux_cHx^2+tds.dflux_cHy^2+tds.dflux_cHz^2)	mol/(m²·s)	Diffusive flux magnitude	Domains 1–3	
tds.tfluxMag_cH	sqrt(tds.tflux_cHx^2+tds.tflux_cHy^2+tds.tflux_cHz^2)	mol/(m²·s)	Total flux magnitude	Domains 1–3	
tds.dpflux_cHx	0	mol/(m²·s)	Dispersive flux, x-component	Domains 1–3	
tds.dpflux_cHy	0	mol/(m²·s)	Dispersive flux, y-component	Domains 1–3	
tds.dpflux_cHz	0	mol/(m²·s)	Dispersive flux, z-component	Domains 1–3	

Name	Expression	Unit	Description	Selection	Details
tds.mflux_cHx	tds.z_cH*F_const*cH*(-tds.um_cHxx*d(tds.V,x)-tds.um_cHxy*d(tds.V,y)-tds.um_cHxz*d(tds.V,z))	mol/(m²·s)	Electrophoretic flux, x-component	Domains 1–3	
tds.mflux_cHy	tds.z_cH*F_const*cH*(-tds.um_cHyx*d(tds.V,x)-tds.um_cHyy*d(tds.V,y)-tds.um_cHyz*d(tds.V,z))	mol/(m²·s)	Electrophoretic flux, y-component	Domains 1–3	
tds.mflux_cHz	tds.z_cH*F_const*cH*(-tds.um_cHzx*d(tds.V,x)-tds.um_cHzy*d(tds.V,y)-tds.um_cHzz*d(tds.V,z))	mol/(m²·s)	Electrophoretic flux, z-component	Domains 1–3	
tds.mfluxMag_cH	sqrt(tds.mflux_cHx^2+tds.mflux_cHy^2+tds.mflux_cHz^2)	mol/(m²·s)	Electrophoretic flux magnitude	Domains 1–3	
tds.tflux_cSO4x	tds.dflux_cSO4x+tds.mflux_cSO4x	mol/(m²·s)	Total flux, x-component	Domains 1–3	+ operation
tds.tflux_cSO4y	tds.dflux_cSO4y+tds.mflux_cSO4y	mol/(m²·s)	Total flux, y-component	Domains 1–3	+ operation
tds.tflux_cSO4z	tds.dflux_cSO4z+tds.mflux_cSO4z	mol/(m²·s)	Total flux, z-component	Domains 1–3	+ operation
tds.dfluxMag_cSO4	sqrt(tds.dflux_cSO4x^2+tds.dflux_cSO4y^2+tds.dflux_cSO4z^2)	mol/(m²·s)	Diffusive flux magnitude	Domains 1–3	
tds.tfluxMag_cSO4	sqrt(tds.tflux_cSO4x^2+tds.tflux_cSO4y^2+tds.tflux_cSO4z^2)	mol/(m²·s)	Total flux magnitude	Domains 1–3	
tds.dpflux_cSO4x	0	mol/(m²·s)	Dispersive flux, x-component	Domains 1–3	

Name	Expression	Unit	Description	Selection	Details
tds.dpflux_cSO4y	0	mol/(m²·s)	Dispersive flux, y-component	Domains 1–3	
tds.dpflux_cSO4z	0	mol/(m²·s)	Dispersive flux, z-component	Domains 1–3	
tds.mflux_cSO4x	tds.z_cSO4*F_const*cSO4*(-tds.um_cSO4xx*d(tds.V,x)-tds.um_cSO4xy*d(tds.V,y)-tds.um_cSO4xz*d(tds.V,z))	mol/(m²·s)	Electrophoretic flux, x-component	Domains 1–3	
tds.mflux_cSO4y	tds.z_cSO4*F_const*cSO4*(-tds.um_cSO4yx*d(tds.V,x)-tds.um_cSO4yy*d(tds.V,y)-tds.um_cSO4yz*d(tds.V,z))	mol/(m²·s)	Electrophoretic flux, y-component	Domains 1–3	
tds.mflux_cSO4z	tds.z_cSO4*F_const*cSO4*(-tds.um_cSO4zx*d(tds.V,x)-tds.um_cSO4zy*d(tds.V,y)-tds.um_cSO4zz*d(tds.V,z))	mol/(m²·s)	Electrophoretic flux, z-component	Domains 1–3	
tds.mfluxMag_cSO4	sqrt(tds.mflux_cSO4x^2+tds.mflux_cSO4y^2+tds.mflux_cSO4z^2)	mol/(m²·s)	Electrophoretic flux magnitude	Domains 1–3	
tds.cAu_material	cAu*spatial.detF	mol/m³	Concentration	Domains 1–3	
tds.dflux_cAux	-tds.D_cAuxx*cAux-tds.D_cAuxy*cAuy-tds.D_cAuxz*cAuz	mol/(m²·s)	Diffusive flux, x-component	Domains 1–3	+ operation
tds.dflux_cAuy	-tds.D_cAuyx*cAux-tds.D_cAuyy*cAuy-tds.D_cAuyz*cAuz	mol/(m²·s)	Diffusive flux, y-component	Domains 1–3	+ operation
tds.dflux_cAuz	-tds.D_cAuzx*cAux-	mol/(m²·s)	Diffusive flux, z-component	Domains 1–3	+ operation

Name	Expression	Unit	Description	Selection	Details
	tds.D_cAuzy*cAuy-tds.D_cAuzz*cAuz				
tds.grad_cAux	cAux	mol/m^4	Concentration gradient, x-component	Domains 1–3	
tds.grad_cAuy	cAuy	mol/m^4	Concentration gradient, y-component	Domains 1–3	
tds.grad_cAuz	cAuz	mol/m^4	Concentration gradient, z-component	Domains 1–3	
tds.cCu_material	cCu*spatial.detF	mol/m^3	Concentration	Domains 1–3	
tds.dflux_cCux	-tds.D_cCuxx*cCux-tds.D_cCuxy*cCuy-tds.D_cCuxz*cCuz	mol/(m^2·s)	Diffusive flux, x-component	Domains 1–3	+ operation
tds.dflux_cCuy	-tds.D_cCuyx*cCux-tds.D_cCuyy*cCuy-tds.D_cCuyz*cCuz	mol/(m^2·s)	Diffusive flux, y-component	Domains 1–3	+ operation
tds.dflux_cCuz	-tds.D_cCuzx*cCux-tds.D_cCuzy*cCuy-tds.D_cCuzz*cCuz	mol/(m^2·s)	Diffusive flux, z-component	Domains 1–3	+ operation
tds.grad_cCux	cCux	mol/m^4	Concentration gradient, x-component	Domains 1–3	
tds.grad_cCuy	cCuy	mol/m^4	Concentration gradient, y-component	Domains 1–3	
tds.grad_cCuz	cCuz	mol/m^4	Concentration gradient, z-component	Domains 1–3	
tds.cH_material	cH*spatial.detF	mol/m^3	Concentration	Domains 1–3	
tds.dflux_cHx	-tds.D_cHxx*cHx-tds.D_cHxy*cHy-tds.D_cHxz*cHz	mol/(m^2·s)	Diffusive flux, x-component	Domains 1–3	+ operation
tds.dflux_cHy	-tds.D_cHyx*cHx-tds.D_cHyy*cHy-tds.D_cHyz*cHz	mol/(m^2·s)	Diffusive flux, y-component	Domains 1–3	+ operation
tds.dflux_cHz	-tds.D_cHzx*cHx-tds.D_cHzy*cHy-tds.D_cHzz*cHz	mol/(m^2·s)	Diffusive flux, z-component	Domains 1–3	+ operation

Name	Expression	Unit	Description	Selection	Details
tds.grad_cHx	cHx	mol/m^4	Concentration gradient, x-component	Domains 1–3	
tds.grad_cHy	cHy	mol/m^4	Concentration gradient, y-component	Domains 1–3	
tds.grad_cHz	cHz	mol/m^4	Concentration gradient, z-component	Domains 1–3	
tds.cSO4_material	cSO4*spatial.detF	mol/m^3	Concentration	Domains 1–3	
tds.dflux_cSO4x	-tds.D_cSO4xx*cSO4x-tds.D_cSO4xy*cSO4y-tds.D_cSO4xz*cSO4z	mol/(m^2·s)	Diffusive flux, x-component	Domains 1–3	+ operation
tds.dflux_cSO4y	-tds.D_cSO4yx*cSO4x-tds.D_cSO4yy*cSO4y-tds.D_cSO4yz*cSO4z	mol/(m^2·s)	Diffusive flux, y-component	Domains 1–3	+ operation
tds.dflux_cSO4z	-tds.D_cSO4zx*cSO4x-tds.D_cSO4zy*cSO4y-tds.D_cSO4zz*cSO4z	mol/(m^2·s)	Diffusive flux, z-component	Domains 1–3	+ operation
tds.grad_cSO4x	cSO4x	mol/m^4	Concentration gradient, x-component	Domains 1–3	
tds.grad_cSO4y	cSO4y	mol/m^4	Concentration gradient, y-component	Domains 1–3	
tds.grad_cSO4z	cSO4z	mol/m^4	Concentration gradient, z-component	Domains 1–3	
tds.um_cAuxx	tds.D_cAuxx/(R_const*tds.T)	s·mol/kg	Mobility, xx-component	Domains 1–3	

Name	Expression	Unit	Description	Selection	Details
tds.um_cAuyx	tds.D_cAuyx/(R_const*tds.T)	s·mol/kg	Mobility, yx-component	Domains 1–3	
tds.um_cAuzx	tds.D_cAuzx/(R_const*tds.T)	s·mol/kg	Mobility, zx-component	Domains 1–3	
tds.um_cAuxy	tds.D_cAuxy/(R_const*tds.T)	s·mol/kg	Mobility, xy-component	Domains 1–3	
tds.um_cAuyy	tds.D_cAuyy/(R_const*tds.T)	s·mol/kg	Mobility, yy-component	Domains 1–3	
tds.um_cAuzy	tds.D_cAuzy/(R_const*tds.T)	s·mol/kg	Mobility, zy-component	Domains 1–3	
tds.um_cAuxz	tds.D_cAuxz/(R_const*tds.T)	s·mol/kg	Mobility, xz-component	Domains 1–3	
tds.um_cAuyz	tds.D_cAuyz/(R_const*tds.T)	s·mol/kg	Mobility, yz-component	Domains 1–3	
tds.um_cAuzz	tds.D_cAuzz/(R_const*tds.T)	s·mol/kg	Mobility, zz-component	Domains 1–3	
tds.um_cCuxx	tds.D_cCuxx/(R_const*tds.T)	s·mol/kg	Mobility, xx-component	Domains 1–3	
tds.um_cCuyx	tds.D_cCuyx/(R_const*tds.T)	s·mol/kg	Mobility, yx-component	Domains 1–3	
tds.um_cCuzx	tds.D_cCuzx/(R_const*tds.T)	s·mol/kg	Mobility, zx-component	Domains 1–3	
tds.um_cCuxy	tds.D_cCuxy/(R_const*tds.T)	s·mol/kg	Mobility, xy-component	Domains 1–3	
tds.um_cCuyy	tds.D_cCuyy/(R_const*tds.T)	s·mol/kg	Mobility, yy-component	Domains 1–3	
tds.um_cCuzy	tds.D_cCuzy/(R_const*tds.T)	s·mol/kg	Mobility, zy-component	Domains 1–3	
tds.um_cCuxz	tds.D_cCuxz/(R_const*tds.T)	s·mol/kg	Mobility, xz-component	Domains 1–3	
tds.um_cCuyz	tds.D_cCuyz/(R_const*tds.T)	s·mol/kg	Mobility, yz-component	Domains 1–3	
tds.um_cCuzz	tds.D_cCuzz/(R_const*tds.T)	s·mol/kg	Mobility, zz-component	Domains 1–3	
tds.um_cHxx	tds.D_cHxx/(R_const*tds.T)	s·mol/kg	Mobility, xx-component	Domains 1–3	
tds.um_cHyx	tds.D_cHyx/(R_const*tds.T)	s·mol/kg	Mobility, yx-component	Domains 1–3	
tds.um_cHzx	tds.D_cHzx/(R_const*tds.T)	s·mol/kg	Mobility, zx-component	Domains 1–3	

Name	Expression	Unit	Description	Selection	Details
tds.um_cHxy	tds.D_cHxy/(R_const*tds.T)	s·mol/kg	Mobility, xy-component	Domains 1–3	
tds.um_cHyy	tds.D_cHyy/(R_const*tds.T)	s·mol/kg	Mobility, yy-component	Domains 1–3	
tds.um_cHzy	tds.D_cHzy/(R_const*tds.T)	s·mol/kg	Mobility, zy-component	Domains 1–3	
tds.um_cHxz	tds.D_cHxz/(R_const*tds.T)	s·mol/kg	Mobility, xz-component	Domains 1–3	
tds.um_cHyz	tds.D_cHyz/(R_const*tds.T)	s·mol/kg	Mobility, yz-component	Domains 1–3	
tds.um_cHzz	tds.D_cHzz/(R_const*tds.T)	s·mol/kg	Mobility, zz-component	Domains 1–3	
tds.um_cSO4xx	tds.D_cSO4xx/(R_const*tds.T)	s·mol/kg	Mobility, xx-component	Domains 1–3	
tds.um_cSO4yx	tds.D_cSO4yx/(R_const*tds.T)	s·mol/kg	Mobility, yx-component	Domains 1–3	
tds.um_cSO4zx	tds.D_cSO4zx/(R_const*tds.T)	s·mol/kg	Mobility, zx-component	Domains 1–3	
tds.um_cSO4xy	tds.D_cSO4xy/(R_const*tds.T)	s·mol/kg	Mobility, xy-component	Domains 1–3	
tds.um_cSO4yy	tds.D_cSO4yy/(R_const*tds.T)	s·mol/kg	Mobility, yy-component	Domains 1–3	
tds.um_cSO4zy	tds.D_cSO4zy/(R_const*tds.T)	s·mol/kg	Mobility, zy-component	Domains 1–3	
tds.um_cSO4xz	tds.D_cSO4xz/(R_const*tds.T)	s·mol/kg	Mobility, xz-component	Domains 1–3	
tds.um_cSO4yz	tds.D_cSO4yz/(R_const*tds.T)	s·mol/kg	Mobility, yz-component	Domains 1–3	
tds.um_cSO4zz	tds.D_cSO4zz/(R_const*tds.T)	s·mol/kg	Mobility, zz-component	Domains 1–3	
tds.V	model.input.V	V	Electric potential	Domains 1–3	Meta
tds.T	tds.cdm1.minput_temperature	K	Temperature	Domains 1–3	
tds.Rlin_cAu	0	1/s	Linear source term coefficient	Domains 1–3	+ operation
tds.Res_cAu	d(cAu*tds.z_cAu*F_const*(-tds.um_cAuxx*d(tds.V,x)-tds.um_cAuxy*d(tds.V,y)-	mol/(m³·s)	Equation residual	Domains 1–3	

Name	Expression	Unit	Description	Selection	Details
	tds.um_cAuxz*d(tds.V,z)),x)+d(cAu*tds.z_cAu*F_const*(-tds.um_cAuyx*d(tds.V,x)-tds.um_cAuyy*d(tds.V,y)-tds.um_cAuyz*d(tds.V,z)),y)+d(cAu*tds.z_cAu*F_const*(-tds.um_cAuzx*d(tds.V,x)-tds.um_cAuzy*d(tds.V,y)-tds.um_cAuzz*d(tds.V,z)),z)-cAu*tds.Rlin_cAu-tds.R_cAu				
tds.Rlin_cCu	0	1/s	Linear source term coefficient	Domains 1–3	+ operation
tds.Res_cCu	d(cCu*tds.z_cCu*F_const*(-tds.um_cCuxx*d(tds.V,x)-tds.um_cCuxy*d(tds.V,y)-tds.um_cCuxz*d(tds.V,z)),x)+d(cCu*tds.z_cCu*F_const*(-tds.um_cCuyx*d(tds.V,x)-tds.um_cCuyy*d(tds.V,y)-tds.um_cCuyz*d(tds.V,z)),y)+d(cCu*tds.z_cCu*F_const*(-tds.um_cCuzx*d(tds.V,x)-tds.um_cCuzy*d(tds.V,y)-tds.um_cCuzz*d(tds.V,z)),z)-cCu*tds.Rlin_cCu-tds.R_cCu	mol/(m^3·s)	Equation residual	Domains 1–3	
tds.Rlin_cH	0	1/s	Linear source term coefficient	Domains 1–3	+ operation

Name	Expression	Unit	Description	Selection	Details
tds.Res_cH	d(cH*tds.z_cH*F_const*(-tds.um_cHxx*d(tds.V,x)-tds.um_cHxy*d(tds.V,y)-tds.um_cHxz*d(tds.V,z)),x)+d(cH*tds.z_cH*F_const*(-tds.um_cHyx*d(tds.V,x)-tds.um_cHyy*d(tds.V,y)-tds.um_cHyz*d(tds.V,z)),y)+d(cH*tds.z_cH*F_const*(-tds.um_cHzx*d(tds.V,x)-tds.um_cHzy*d(tds.V,y)-tds.um_cHzz*d(tds.V,z)),z)-cH*tds.Rlin_cH-tds.R_cH	mol/(m³·s)	Equation residual	Domains 1–3	
tds.Rlin_cSO4	0	1/s	Linear source term coefficient	Domains 1–3	+ operation
tds.Res_cSO4	d(cSO4*tds.z_cSO4*F_const*(-tds.um_cSO4xx*d(tds.V,x)-tds.um_cSO4xy*d(tds.V,y)-tds.um_cSO4xz*d(tds.V,z)),x)+d(cSO4*tds.z_cSO4*F_const*(-tds.um_cSO4yx*d(tds.V,x)-tds.um_cSO4yy*d(tds.V,y)-tds.um_cSO4yz*d(tds.V,z)),y)+d(cSO4*tds.z_cSO4*F_const*(-tds.um_cSO4zx*d(tds.V,x)-tds.um_cSO4zy*d(t	mol/(m³·s)	Equation residual	Domains 1–3	

Name	Expression	Unit	Description	Selection	Details
	ds.V,y)-tds.um_cSO4zz*d(tds.V,z)),z)-cSO4*tds.Rlin_cSO4-tds.R_cSO4				

Shape functions

Name	Shape function	Unit	Description	Shape frame	Selection
cAu	Lagrange (Linear)	mol/m^3	Concentration	Spatial	Domains 1–3
cCu	Lagrange (Linear)	mol/m^3	Concentration	Spatial	Domains 1–3
cH	Lagrange (Linear)	mol/m^3	Concentration	Spatial	Domains 1–3
cSO4	Lagrange (Linear)	mol/m^3	Concentration	Spatial	Domains 1–3

Weak Expressions

Weak expression	Integration order	Integration frame	Selection
(tds.dflux_cAux*test(cAux)+tds.dflux_cAuy*test(cAuy)+tds.dflux_cAuz*test(cAuz))*tds.d	2	Spatial	Domains 1–3
(tds.dflux_cCux*test(cCux)+tds.dflux_cCuy*test(cCuy)+tds.dflux_cCuz*test(cCuz))*tds.d	2	Spatial	Domains 1–3
(tds.dflux_cHx*test(cHx)+tds.dflux_cHy*test(cHy)+tds.dflux_cHz*test(cHz))*tds.d	2	Spatial	Domains 1–3
(tds.dflux_cSO4x*test(cSO4x)+tds.dflux_cSO4y*test(cSO4y)+tds.dflux_cSO4z*test(cSO4z))*tds.d	2	Spatial	Domains 1–3
tds.z_cAu*F_const*cAu*((-tds.um_cAuxx*d(tds.V,x)-tds.um_cAuxy*d(tds.V,y)-tds.um_cAuxz*d(tds.V,z))*test(cAux)+(-tds.um_cAuyx*d(tds.V,x)-tds.um_cAuyy*d(tds.V,y)-tds.um_cAuyz*d(tds.V,z))*test(cAuy)+(-tds.um_cAuzx*d(tds.V,x)-tds.um_cAuzy*d(tds.V,y)-tds.um_cAuzz*d(tds.V,z))*test(cAuz))*tds.d	2	Spatial	Domains 1–3
tds.z_cCu*F_const*cCu*((-tds.um_cCuxx*d(tds.V,x)-tds.um_cCuxy*d(tds.V,y)-tds.um_cCuxz*d(tds.V,z))*test(cCux)+(-tds.um_cCuyx*d(tds.V,x)-	2	Spatial	Domains 1–3

Weak expression	Integration order	Integration frame	Selection
tds.um_cCuyy*d(tds.V,y)-tds.um_cCuyz*d(tds.V,z))*test(cCuy)+(-tds.um_cCuzx*d(tds.V,x)-tds.um_cCuzy*d(tds.V,y)-tds.um_cCuzz*d(tds.V,z))*test(cCuz))*tds.d			
tds.z_cH*F_const*cH*((-tds.um_cHxx*d(tds.V,x)-tds.um_cHxy*d(tds.V,y)-tds.um_cHxz*d(tds.V,z))*test(cHx)+(-tds.um_cHyx*d(tds.V,x)-tds.um_cHyy*d(tds.V,y)-tds.um_cHyz*d(tds.V,z))*test(cHy)+(-tds.um_cHzx*d(tds.V,x)-tds.um_cHzy*d(tds.V,y)-tds.um_cHzz*d(tds.V,z))*test(cHz))*tds.d	2	Spatial	Domains 1–3
tds.z_cSO4*F_const*cSO4*((-tds.um_cSO4xx*d(tds.V,x)-tds.um_cSO4xy*d(tds.V,y)-tds.um_cSO4xz*d(tds.V,z))*test(cSO4x)+(-tds.um_cSO4yx*d(tds.V,x)-tds.um_cSO4yy*d(tds.V,y)-tds.um_cSO4yz*d(tds.V,z))*test(cSO4y)+(-tds.um_cSO4zx*d(tds.V,x)-tds.um_cSO4zy*d(tds.V,y)-tds.um_cSO4zz*d(tds.V,z))*test(cSO4z))*tds.d	2	Spatial	Domains 1–3
tds.streamline*(isScalingSystemDomain==0)*tds.d	2	Spatial	Domains 1–3
tds.crosswind*(isScalingSystemDomain==0)*tds.d	4	Spatial	Domains 1–3

2.4.5 No Flux 1

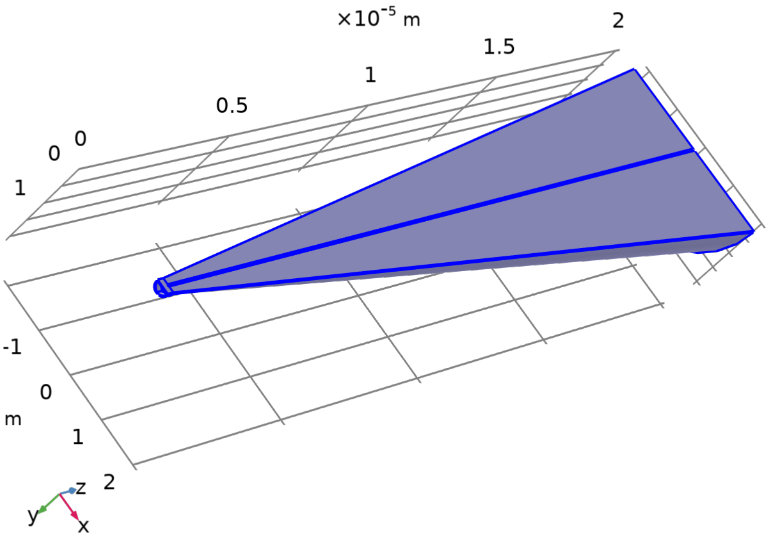

No Flux 1

SELECTION

Geometric entity level	Boundary
Selection	Geometry geom1: Dimension 2: All boundaries

EQUATIONS

$$-\mathbf{n} \cdot \mathbf{J}_i = 0$$

2.4.6 Initial Values 1

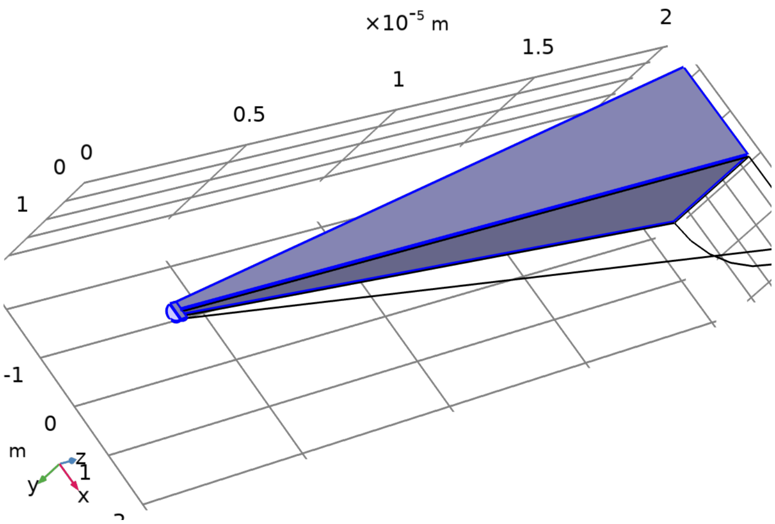

Initial Values 1

SELECTION

Geometric entity level	Domain
Selection	Geometry geom1: Dimension 3: All domains

Initial Values

SETTINGS

Description	Value	Unit
Concentration	{c0Au, c0Cu, 2*c0H2SO4, c0H2SO4}	mol/m³

Variables

Name	Expression	Unit	Description	Selection	Details
tds.c0_cAu	c0Au	mol/m³	Concentration	Domains 1–2	+ operation
tds.c0_cCu	c0Cu	mol/m³	Concentration	Domains 1–2	+ operation
tds.c0_cH	2*c0H2SO4	mol/m³	Concentration	Domains 1–2	+ operation
tds.c0_cSO4	c0H2SO4	mol/m³	Concentration	Domains 1–2	+ operation

2.4.7 Initial Values 2

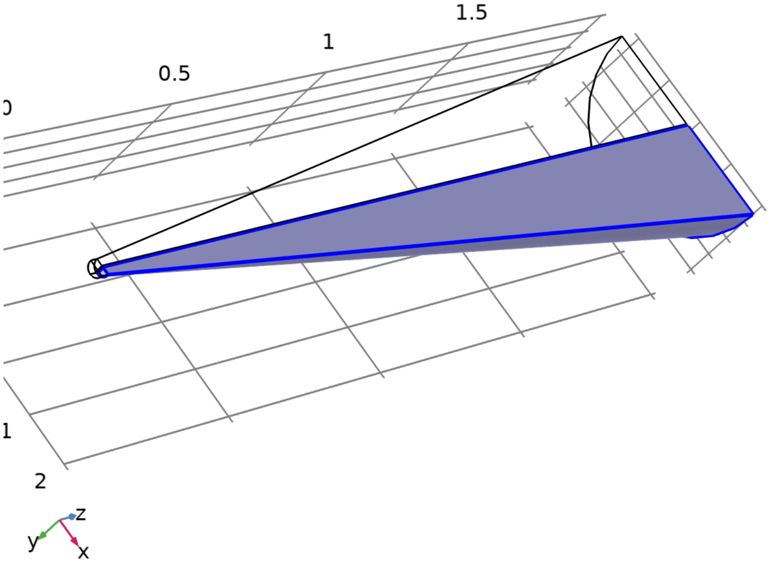

Initial Values 2

SELECTION

Geometric entity level	Domain
Selection	Geometry geom1: Dimension 3: Domain 3

Initial Values

SETTINGS

Description	Value	Unit
Concentration	{0.001*c0Au, 0.001*c0Cu, 2*c0H2SO42, c0H2SO42}	mol/m^3

Variables

Name	Expression	Unit	Description	Selection	Details
tds.c0_cAu	0.001*c0Au	mol/m^3	Concentration	Domain 3	+ operation
tds.c0_cCu	0.001*c0Cu	mol/m^3	Concentration	Domain 3	+ operation
tds.c0_cH	2*c0H2SO42	mol/m^3	Concentration	Domain 3	+ operation
tds.c0_cSO4	c0H2SO42	mol/m^3	Concentration	Domain 3	+ operation

2.4.8 Concentration 1

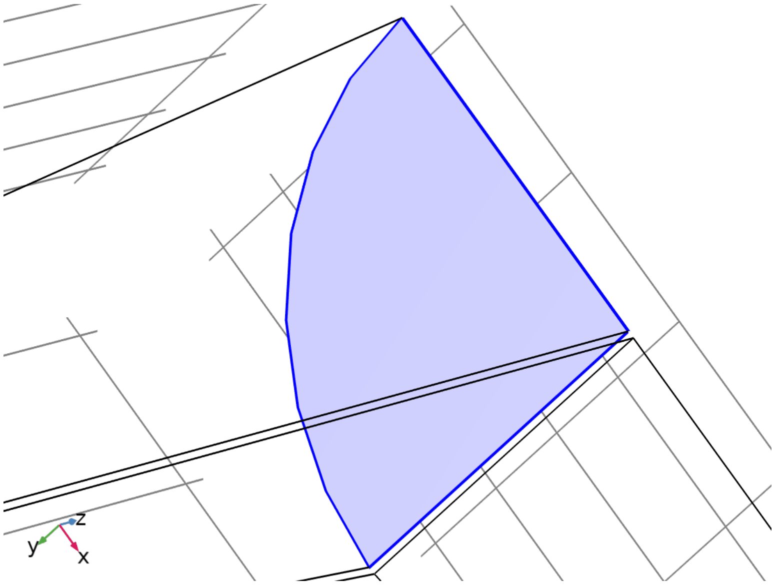

Concentration 1

SELECTION

Geometric entity level	Boundary
Selection	Geometry geom1: Dimension 2: Boundary 3

EQUATIONS

$$c_i = c_{0,j}$$

Concentration

SETTINGS

Description	Value	Unit
Species cAu	On	
Species cCu	On	

Description	Value	Unit
Species cH	On	
Species cSO4	On	
Concentration	{c0Au, c0Cu, 2*c0H2SO4 + c0Au, c0H2SO4 + c0Cu}	mol/m³

Constraint Settings

SETTINGS

Description	Value
Constraint	Pointwise constraints
Apply reaction terms on	All physics (symmetric)
Constraint method	Elemental

Variables

Name	Expression	Unit	Description	Selection	Details
tds.c0_cAu	c0Au	mol/m³	Concentration	Boundary 3	+ operation
tds.c0_cCu	c0Cu	mol/m³	Concentration	Boundary 3	+ operation
tds.c0_cH	2*c0H2SO4+c0Au	mol/m³	Concentration	Boundary 3	+ operation
tds.c0_cSO4	c0H2SO4+c0Cu	mol/m³	Concentration	Boundary 3	+ operation
tds.conc1.nmflow_cAu	tds.conc1.int(tds.ntflux_cAu)*tds.d	mol/s	Normal molar flow rate	Global	
tds.conc1.nmflow_cCu	tds.conc1.int(tds.ntflux_cCu)*tds.d	mol/s	Normal molar flow rate	Global	
tds.conc1.nmflow_cH	tds.conc1.int(tds.ntflux_cH)*tds.d	mol/s	Normal molar flow rate	Global	
tds.conc1.nmflow_cSO4	tds.conc1.int(tds.ntflux_cSO4)*tds.d	mol/s	Normal molar flow rate	Global	

Constraints

Constraint	Constraint force	Shape function	Selection	Details
-tds.cVar_cAu+tds.c0_cAu	test(-tds.cVar_cAu+tds.c0_cAu)	Lagrange (Linear)	Boundary 3	Elemental
-tds.cVar_cCu+tds.c0_cCu	test(-tds.cVar_cCu+tds.c0_cCu)	Lagrange (Linear)	Boundary 3	Elemental
-tds.cVar_cH+tds.c0_cH	test(-tds.cVar_cH+tds.c0_cH)	Lagrange (Linear)	Boundary 3	Elemental

Constraint	Constraint force	Shape function	Selection	Details
-tds.cVar_cSO4+tds.c0_cSO4	test(-tds.cVar_cSO4+tds.c0_cSO4)	Lagrange (Linear)	Boundary 3	Elemental

2.4.9 Concentration 2

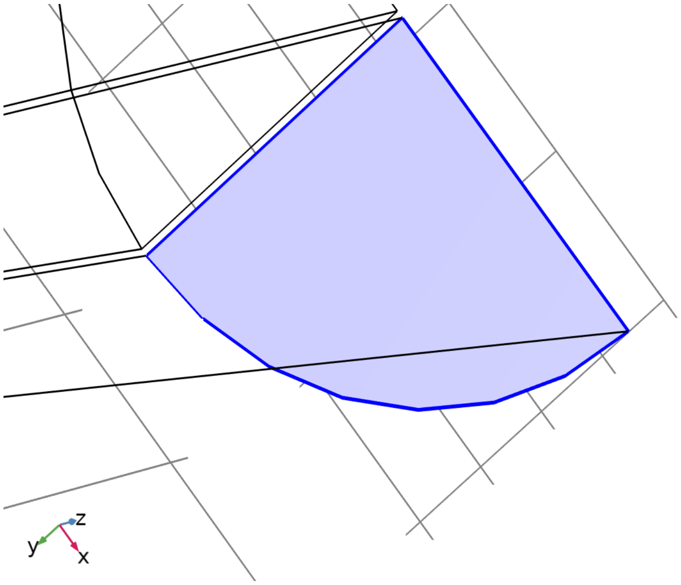

Concentration 2

SELECTION

Geometric entity level	Boundary
Selection	Geometry geom1: Dimension 2: Boundary 15

EQUATIONS

$$c_i = c_{0,j}$$

Concentration

SETTINGS

Description	Value	Unit
Species cAu	On	
Species cCu	On	
Species cH	On	
Species cSO4	On	
Concentration	{0.001*c0Au, 0.001*c0Cu, 2*c0H2SO42 + 0.001*c0Au, c0H2SO42 + 0.001*c0Cu}	mol/m^3

Constraint Settings

SETTINGS

Description	Value
Constraint	Pointwise constraints
Apply reaction terms on	All physics (symmetric)
Constraint method	Elemental

Variables

Name	Expression	Unit	Description	Selection	Details
tds.c0_cAu	0.001*c0Au	mol/m³	Concentration	Boundary 15	+ operation
tds.c0_cCu	0.001*c0Cu	mol/m³	Concentration	Boundary 15	+ operation
tds.c0_cH	2*c0H2SO42+0.001*c0Au	mol/m³	Concentration	Boundary 15	+ operation
tds.c0_cSO4	c0H2SO42+0.001*c0Cu	mol/m³	Concentration	Boundary 15	+ operation
tds.conc2.nmflow_cAu	tds.conc2.int(tds.ntflux_cAu)*tds.d	mol/s	Normal molar flow rate	Global	
tds.conc2.nmflow_cCu	tds.conc2.int(tds.ntflux_cCu)*tds.d	mol/s	Normal molar flow rate	Global	
tds.conc2.nmflow_cH	tds.conc2.int(tds.ntflux_cH)*tds.d	mol/s	Normal molar flow rate	Global	
tds.conc2.nmflow_cSO4	tds.conc2.int(tds.ntflux_cSO4)*tds.d	mol/s	Normal molar flow rate	Global	

Constraints

Constraint	Constraint force	Shape function	Selection	Details
-tds.cVar_cAu+tds.c0_cAu	test(-tds.cVar_cAu+tds.c0_cAu)	Lagrange (Linear)	Boundary 15	Elemental
-tds.cVar_cCu+tds.c0_cCu	test(-tds.cVar_cCu+tds.c0_cCu)	Lagrange (Linear)	Boundary 15	Elemental
-tds.cVar_cH+tds.c0_cH	test(-tds.cVar_cH+tds.c0_cH)	Lagrange (Linear)	Boundary 15	Elemental
-tds.cVar_cSO4+tds.c0_cSO4	test(-tds.cVar_cSO4+tds.c0_cSO4)	Lagrange (Linear)	Boundary 15	Elemental

2.4.10 Concentration 3

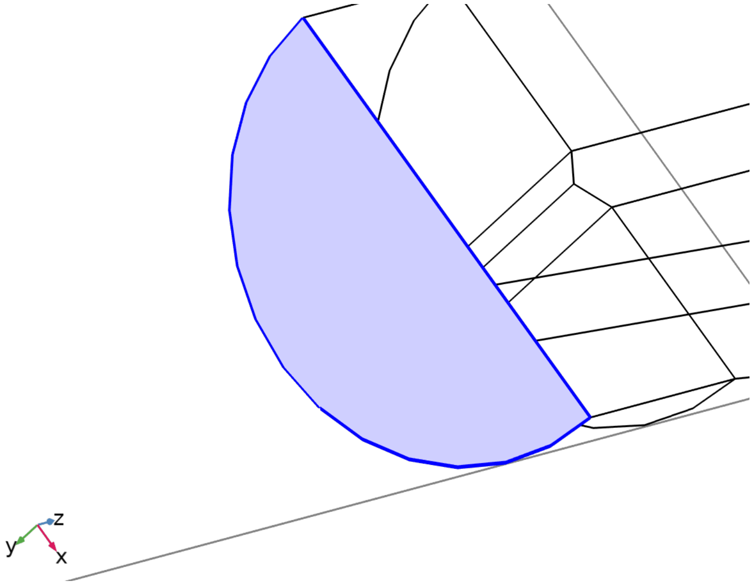

Concentration 3

SELECTION

Geometric entity level	Boundary
Selection	Geometry geom1: Dimension 2: Boundary 6

EQUATIONS

$$c_i = c_{0,i}$$

Concentration

SETTINGS

Description	Value	Unit
Species cAu	On	
Species cCu	On	
Species cH	Off	
Species cSO4	Off	
Concentration	{0, 0, 0, 0}	mol/m^3

Constraint Settings

SETTINGS

Description	Value
Constraint	Pointwise constraints
Apply reaction terms on	All physics (symmetric)
Constraint method	Elemental

Variables

Name	Expression	Unit	Description	Selection	Details
tds.c0_cAu	0	mol/m³	Concentration	Boundary 6	+ operation
tds.c0_cCu	0	mol/m³	Concentration	Boundary 6	+ operation
tds.conc3.nmflow_cAu	tds.conc3.int(tds.ntflux_cAu)*tds.d	mol/s	Normal molar flow rate	Global	
tds.conc3.nmflow_cCu	tds.conc3.int(tds.ntflux_cCu)*tds.d	mol/s	Normal molar flow rate	Global	
tds.conc3.nmflow_cH	tds.conc3.int(tds.ntflux_cH)*tds.d	mol/s	Normal molar flow rate	Global	
tds.conc3.nmflow_cSO4	tds.conc3.int(tds.ntflux_cSO4)*tds.d	mol/s	Normal molar flow rate	Global	

Constraints

Constraint	Constraint force	Shape function	Selection	Details
-tds.cVar_cAu+tds.c0_cAu	test(-tds.cVar_cAu+tds.c0_cAu)	Lagrange (Linear)	Boundary 6	Elemental
-tds.cVar_cCu+tds.c0_cCu	test(-tds.cVar_cCu+tds.c0_cCu)	Lagrange (Linear)	Boundary 6	Elemental
0	0	Lagrange (Linear)	Boundary 6	Elemental
0	0	Lagrange (Linear)	Boundary 6	Elemental

2.5 MESH 1

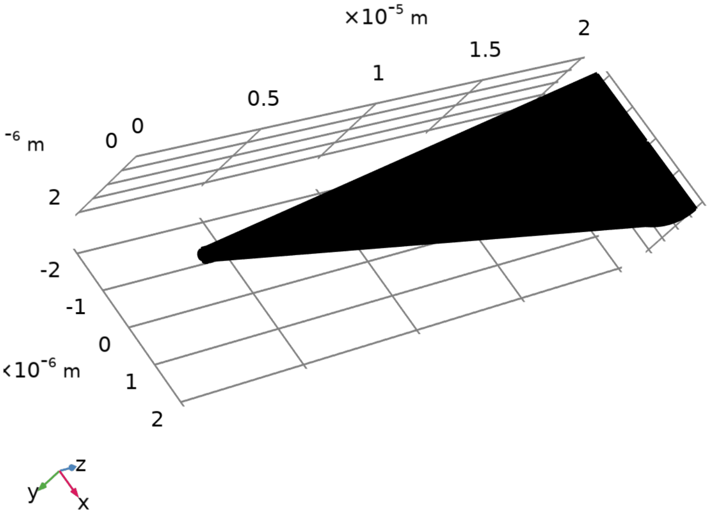

Mesh 1

MESH STATISTICS

Description	Value
Status	Complete mesh
Mesh vertices	468691
Tetrahedra	2408010
Triangles	283032
Edge elements	4913
Vertex elements	17
Number of elements	2408010
Minimum element quality	0.1793
Average element quality	0.6633
Element volume ratio	1.7869E-8
Mesh volume	4.99E-17 m^3

2.5.1 Size (size)

SETTINGS

Description	Value
Maximum element size	4.04E-7
Minimum element size	4.04E-9
Curvature factor	0.2
Maximum element growth rate	1.3
Predefined size	Extremely fine

2.5.2 Free Tetrahedral 2 (ftet2)

SELECTION

Geometric entity level	Domain
Selection	Remaining

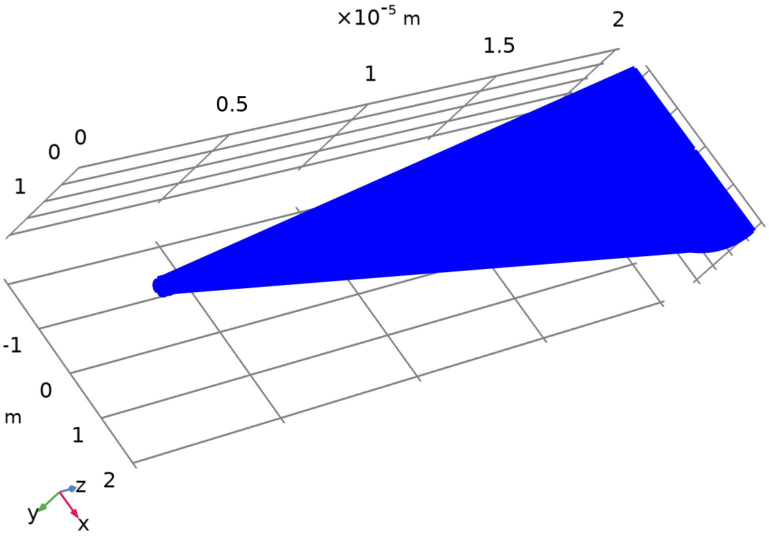

Free Tetrahedral 2

SETTINGS

Description	Value
Avoid inverted curved elements	On

INFORMATION

Description	Value
Last build time	25 seconds
Built with	COMSOL 6.2.0.658 (win64), Jul 28, 2025, 2:36:14 PM

substrate (size1)

SELECTION

Geometric entity level	Boundary
Selection	Geometry geom1: Dimension 2: Boundary 6

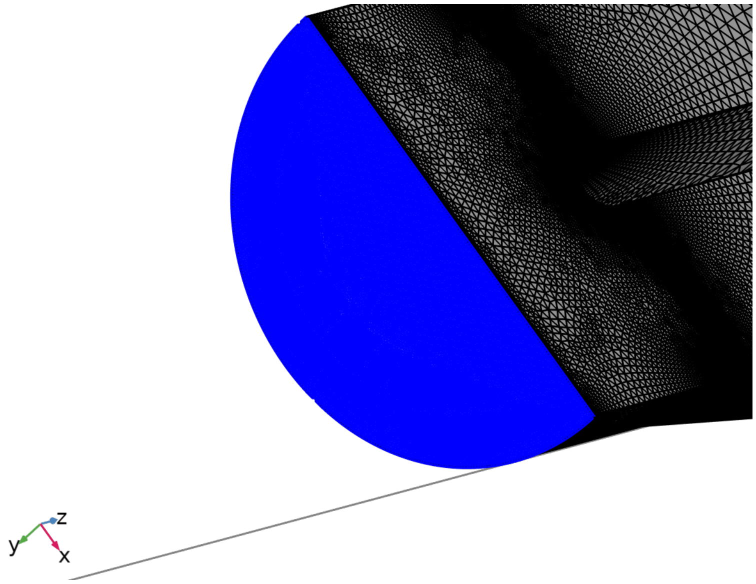

substrate

SETTINGS

Description	Value
Maximum element size	2E-9
Minimum element size	1E-12
Curvature factor	0.2
Predefined size	Extremely fine
Custom element size	Custom

Size 2 (size2)

SELECTION

Geometric entity level	Boundary
Selection	Geometry geom1: Dimension 2: Boundaries 1–2, 8, 12–13, 16

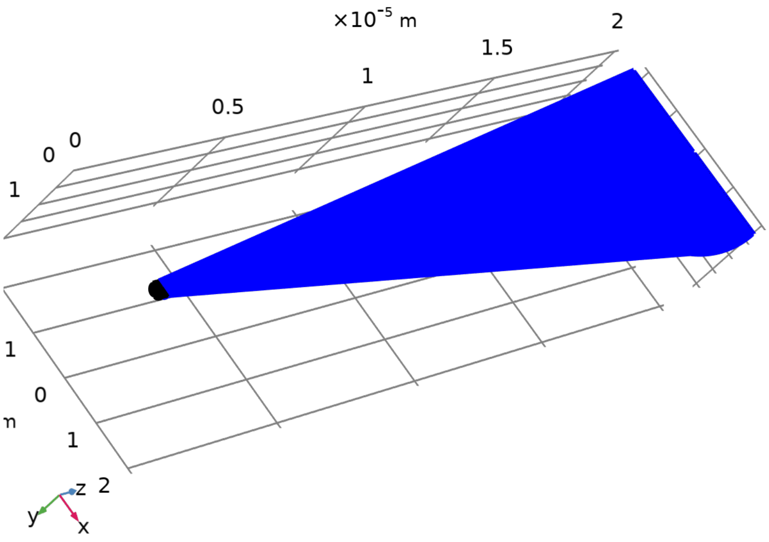

Size 2

SETTINGS

Description	Value
Maximum element size	5E-8
Minimum element size	1E-12
Curvature factor	0.2
Maximum element growth rate	1.05
Predefined size	Extremely fine
Custom element size	Custom

Size 3 (size3)

SELECTION

Geometric entity level	Edge
Selection	Geometry geom1: Dimension 1: Edges 7–8, 10–11, 16, 19, 21–22, 26

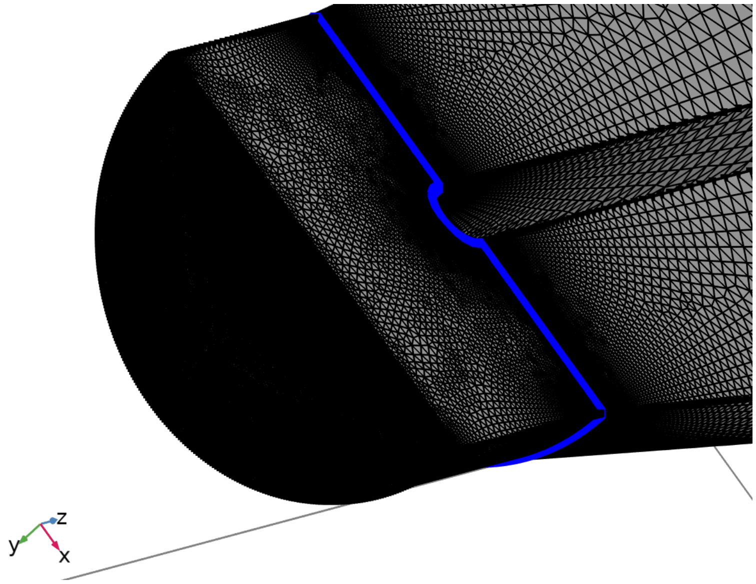

Size 3

SETTINGS

Description	Value
Maximum element size	1E-9
Minimum element size	1E-12
Curvature factor	0.2
Maximum element growth rate	1.05
Predefined size	Extremely fine
Custom element size	Custom

3 Study 1

COMPUTATION INFORMATION

Computation time	13 h 28 min 59 s

3.1 PARAMETRIC SWEEP

Parameter name	Parameter value list	Parameter unit
Vmetal	range(0.13,-0.01,0.01),-1e-6, range(-0.01,-0.01,-0.13)	V

STUDY SETTINGS

Description	Value
Sweep type	Specified combinations
Parameter name	Vmetal
Unit	V

PARAMETERS

Parameter name	Parameter value list	Parameter unit
Vmetal (potential in right barrel)	range(0.13,-0.01,0.01),-1e-6, range(-0.01,-0.01,-0.13)	V

3.2 STATIONARY

STUDY SETTINGS

Description	Value
Include geometric nonlinearity	Off

PHYSICS AND VARIABLES SELECTION

Physics interface	Solve for	Equation form
Electric Currents (ec)	On	Automatic (Stationary)
Transport of Diluted Species (tds)	On	Automatic (Stationary)

STORE IN OUTPUT

Interface	Output	Selection
Electric Currents (ec)	Physics controlled	
Transport of Diluted Species (tds)	Physics controlled	

MESH SELECTION

Component	Mesh
Component 1	Mesh 1

3.3 SOLVER CONFIGURATIONS

3.3.1 Solution 1

Compile Equations: Stationary (st1)

STUDY AND STEP

Description	Value
Use study	Study 1
Use study step	Stationary

LOG

```
<---- Compile Equations: Stationary in Study 1/Solution 1 (sol1) ---------------
Started at Sep 23, 2025, 4:41:16 PM.
Geometry shape function: Linear Lagrange
Running on Intel64 Family 6 Model 165 Stepping 5, GenuineIntel.
Using 1 socket with 10 cores in total on DESKTOP-QEOL9O1.
Available memory: 130.97 GB.
Time: 17 s.
Physical memory: 4.95 GB
Virtual memory: 5.78 GB
Ended at Sep 23, 2025, 4:41:33 PM.
----- Compile Equations: Stationary in Study 1/Solution 1 (sol1) -------------->
```

Dependent Variables 1 (v1)

GENERAL

Description	Value
Defined by study step	Step 1: Stationary

INITIAL VALUE CALCULATION CONSTANTS

Constant name	Initial value source
Vmetal	range(0.13,-0.01,0.01)[V] -1e-6[V] range(-0.01,-0.01,-0.13)[V]

LOG

```
<---- Dependent Variables 1 in Study 1/Solution 1 (sol1) -----------------------
Started at Sep 23, 2025, 4:41:33 PM.
Solution time: 4 s.
Physical memory: 4.9 GB
Virtual memory: 5.71 GB
Ended at Sep 23, 2025, 4:41:37 PM.
----- Dependent Variables 1 in Study 1/Solution 1 (sol1) ---------------------->
```

Concentration (comp1.cAu) (comp1_cAu)

GENERAL

Description	Value
Field components	comp1.cAu

Description	Value
Internal variables	{comp1.uflux.cAu, comp1.dflux.cAu}

Concentration (comp1.cH) (comp1_cH)

GENERAL

Description	Value
Field components	comp1.cH
Internal variables	{comp1.uflux.cH, comp1.dflux.cH}

Electric Potential (comp1.V) (comp1_V)

GENERAL

Description	Value
Field components	comp1.V

Concentration (comp1.cCu) (comp1_cCu)

GENERAL

Description	Value
Field components	comp1.cCu
Internal variables	{comp1.uflux.cCu, comp1.dflux.cCu}

Concentration (comp1.cSO4) (comp1_cSO4)

GENERAL

Description	Value
Field components	comp1.cSO4
Internal variables	{comp1.uflux.cSO4, comp1.dflux.cSO4}

Stationary Solver 1 (s1)

GENERAL

Description	Value
Defined by study step	Step 1: Stationary

RESULTS WHILE SOLVING

Description	Value
Probes	None

LOG

```
   1          20   1.0000000          20  417  197  197    2e-14  2.1e-15
Solution error estimates for segregated groups
0.92, 0.82, 20
Residual error estimates for segregated groups
1.4e+03, 9.5e+03, 9.5e+03
Segregated solver iteration 2.
Electric Currents
Iter      SolEst     Damping    Stepsize #Res #Jac #Sol   LinErr   LinRes
   1       0.018   1.0000000       0.018  419  198  198    8e-14  1.1e-11
Concentrations
Iter      SolEst      ResEst     Damping    Stepsize #Res #Jac #Sol   LinErr   LinRe
s
   1        0.43     2.3e+03   0.3500000        0.43  969  550  550  3.2e-15  1.7e-
15
   2         0.4     1.5e+03   0.3500000         0.4  970  551  551    3e-15  1.7e-
15
   3        0.37     9.7e+02   0.3500000        0.37  971  552  552  5.2e-15  1.7e-
15
Segregated Step 1
Iter      SolEst     Damping    Stepsize #Res #Jac #Sol   LinErr   LinRes
   1      0.0031   1.0000000      0.0031  419  198  198  3.4e-14  6.6e-13
Solution error estimates for segregated groups
0.018, 0.64, 0.0031
Residual error estimates for segregated groups
50, 9.7e+02, 97
Segregated solver iteration 3.
Electric Currents
Iter      SolEst     Damping    Stepsize #Res #Jac #Sol   LinErr   LinRes
   1       0.015   1.0000000       0.015  421  199  199  6.2e-14  2.9e-11
Concentrations
Iter      SolEst      ResEst     Damping    Stepsize #Res #Jac #Sol   LinErr   LinRe
s
   1        0.35     6.3e+02   0.3500000        0.35  974  553  553  3.5e-15  1.6e-
15
   2        0.32     4.1e+02   0.3500000        0.32  975  554  554  2.5e-15  1.7e-
15
   3        0.22     2.7e+02   0.3500000        0.22  976  555  555  3.2e-15  1.7e-
15
Segregated Step 1
Iter      SolEst     Damping    Stepsize #Res #Jac #Sol   LinErr   LinRes
   1      0.0018   1.0000000      0.0018  421  199  199  7.9e-15  8.3e-13
Solution error estimates for segregated groups
0.015, 0.38, 0.0018
Residual error estimates for segregated groups
25, 2.7e+02, 38
Segregated solver iteration 4.
Electric Currents
Iter      SolEst     Damping    Stepsize #Res #Jac #Sol   LinErr   LinRes
   1       0.012   1.0000000       0.012  423  200  200  4.1e-14  5.9e-11
Concentrations
Iter      SolEst      ResEst     Damping    Stepsize #Res #Jac #Sol   LinErr   LinRe
s
   1        0.15     1.7e+02   0.3500000        0.15  979  556  556  3.8e-15  1.7e-
15
   2       0.095     1.1e+02   0.3500000       0.095  980  557  557  3.8e-15  1.7e-
15
```

```
   3       0.062          74   0.3500000       0.062  981  558  558  4.6e-15  1.7e-
15
Segregated Step 1
Iter      SolEst     Damping    Stepsize #Res #Jac #Sol   LinErr   LinRes
   1     0.00072   1.0000000     0.00072  423  200  200  1.7e-14  5.8e-13
Solution error estimates for segregated groups
0.012, 0.11, 0.00072
Residual error estimates for segregated groups
25, 74, 23
Segregated solver iteration 5.
Electric Currents
Iter      SolEst     Damping    Stepsize #Res #Jac #Sol   LinErr   LinRes
   1      0.0086   1.0000000      0.0086  425  201  201  1.3e-13  5.8e-11
Concentrations
Iter      SolEst      ResEst     Damping    Stepsize #Res #Jac #Sol   LinErr   LinRe
s
   1       0.041          49   0.3500000       0.041  984  559  559  6.7e-15  1.7e-
15
   2       0.027          33   0.3500000       0.027  985  560  560  3.6e-15  1.7e-
15
   3       0.017          22   0.3500000       0.017  986  561  561  1.1e-14  1.7e-
15
Segregated Step 1
Iter      SolEst     Damping    Stepsize #Res #Jac #Sol   LinErr   LinRes
   1     0.00024   1.0000000     0.00024  425  201  201  2.8e-14  3.2e-13
Solution error estimates for segregated groups
0.0086, 0.03, 0.00024
Residual error estimates for segregated groups
28, 22, 14
Segregated solver iteration 6.
Electric Currents
Iter      SolEst     Damping    Stepsize #Res #Jac #Sol   LinErr   LinRes
   1      0.0055   1.0000000      0.0055  427  202  202  1.4e-13  4.1e-11
Concentrations
Iter      SolEst      ResEst     Damping    Stepsize #Res #Jac #Sol   LinErr   LinRe
s
   1       0.011          17   0.3500000       0.011  989  562  562  1.5e-14    2e-
15
   2      0.0074          14   0.3500000      0.0074  990  563  563  3.2e-14    2e-
15
   3      0.0048          10   0.3500000      0.0048  991  564  564    1e-14    2e-
15
Segregated Step 1
Iter      SolEst     Damping    Stepsize #Res #Jac #Sol   LinErr   LinRes
   1     0.00033   1.0000000     0.00033  427  202  202  2.9e-14  7.8e-13
Solution error estimates for segregated groups
0.0055, 0.0082, 0.00033
Residual error estimates for segregated groups
28, 10, 10
Segregated solver iteration 7.
Electric Currents
Iter      SolEst     Damping    Stepsize #Res #Jac #Sol   LinErr   LinRes
   1      0.0029   1.0000000      0.0029  429  203  203  5.6e-14  2.8e-11
Concentrations
Iter      SolEst      ResEst     Damping    Stepsize #Res #Jac #Sol   LinErr   LinRe
s
```

```
   1      0.0032          12   0.3500000      0.0032  994  565  565  5.6e-14  3.7e-
15
   2      0.0021          11   0.3500000      0.0021  995  566  566  2.9e-14  3.8e-
15
   3      0.0014         9.1   0.3500000      0.0014  996  567  567  2.1e-14  3.8e-
15
Segregated Step 1
Iter      SolEst     Damping    Stepsize #Res #Jac #Sol   LinErr   LinRes
   1     0.00046   1.0000000     0.00046  429  203  203  2.9e-14  1.7e-12
Solution error estimates for segregated groups
0.0029, 0.0023, 0.00046
Residual error estimates for segregated groups
25, 9.1, 11
Segregated solver iteration 8.
Electric Currents
Iter      SolEst     Damping    Stepsize #Res #Jac #Sol   LinErr   LinRes
   1       0.001   1.0000000       0.001  431  204  204  1.8e-13    2e-11
Concentrations
Iter      SolEst      ResEst     Damping    Stepsize #Res #Jac #Sol   LinErr   LinRe
s
   1      0.0011          11   0.3500000      0.0011  999  568  568  1.4e-14  9.8e-
15
   2     0.00069         9.6   0.3500000     0.00069 1000  569  569  4.4e-14  9.4e-
15
Segregated Step 1
Iter      SolEst     Damping    Stepsize #Res #Jac #Sol   LinErr   LinRes
   1     0.00048   1.0000000     0.00048  431  204  204  4.8e-14  2.2e-12
Solution error estimates for segregated groups
0.001, 0.00069, 0.00048
Residual error estimates for segregated groups
17, 9.6, 15
Segregated solver iteration 9.
Electric Currents
Iter      SolEst     Damping    Stepsize #Res #Jac #Sol   LinErr   LinRes
   1     0.00032   1.0000000     0.00032  433  205  205  6.3e-14  1.8e-11
Concentrations
Iter      SolEst      ResEst     Damping    Stepsize #Res #Jac #Sol   LinErr   LinRe
s
   1     0.00062          10   0.3500000     0.00062 1003  570  570    3e-14  1.8e-
14
Segregated Step 1
Iter      SolEst     Damping    Stepsize #Res #Jac #Sol   LinErr   LinRes
   1     0.00036   1.0000000     0.00036  433  205  205  8.2e-15    2e-12
Solution error estimates for segregated groups
0.00032, 0.0004, 0.00036
Residual error estimates for segregated groups
8.8, 10, 11

Parameter Vmetal = -0.11.
Segregated solver iteration 1.
Electric Currents
Iter      SolEst     Damping    Stepsize #Res #Jac #Sol   LinErr   LinRes
   1        0.92   1.0000000        0.92  436  206  206    9e-14  1.4e-14
Concentrations
Iter      SolEst      ResEst     Damping    Stepsize #Res #Jac #Sol   LinErr   LinRe
s
```

```
   1        0.68     5.2e+03   0.3500000        0.68 1007  571  571  5.6e-15  1.6e-
15
   2        0.55       4e+03   0.3500000        0.55 1008  572  572    3e-15  1.6e-
15
   3        0.47     2.6e+03   0.3500000        0.47 1009  573  573  1.9e-15  1.6e-
15
Segregated Step 1
Iter      SolEst     Damping    Stepsize #Res #Jac #Sol   LinErr   LinRes
   1          21   1.0000000          21  436  206  206  1.6e-14  2.1e-15
Solution error estimates for segregated groups
0.92, 0.81, 21
Residual error estimates for segregated groups
1.5e+03, 9.5e+03, 9.5e+03
Segregated solver iteration 2.
Electric Currents
Iter      SolEst     Damping    Stepsize #Res #Jac #Sol   LinErr   LinRes
   1       0.017   1.0000000       0.017  438  207  207    2e-13    1e-11
Concentrations
Iter      SolEst      ResEst     Damping    Stepsize #Res #Jac #Sol   LinErr   LinRe
s
   1        0.43     2.3e+03   0.3500000        0.43 1012  574  574  3.4e-15  1.6e-
15
   2        0.39     1.5e+03   0.3500000        0.39 1013  575  575  3.8e-15  1.6e-
15
   3        0.37     9.6e+02   0.3500000        0.37 1014  576  576  2.6e-15  1.6e-
15
Segregated Step 1
Iter      SolEst     Damping    Stepsize #Res #Jac #Sol   LinErr   LinRes
   1      0.0032   1.0000000      0.0032  438  207  207  4.4e-14  6.1e-13
Solution error estimates for segregated groups
0.017, 0.64, 0.0032
Residual error estimates for segregated groups
52, 9.6e+02, 86
Segregated solver iteration 3.
Electric Currents
Iter      SolEst     Damping    Stepsize #Res #Jac #Sol   LinErr   LinRes
   1       0.015   1.0000000       0.015  440  208  208  1.4e-13  2.8e-11
Concentrations
Iter      SolEst      ResEst     Damping    Stepsize #Res #Jac #Sol   LinErr   LinRe
s
   1        0.35     6.2e+02   0.3500000        0.35 1017  577  577  2.4e-15  1.6e-
15
   2        0.32     4.1e+02   0.3500000        0.32 1018  578  578  2.4e-15  1.7e-
15
   3        0.22     2.6e+02   0.3500000        0.22 1019  579  579  7.6e-15  1.7e-
15
Segregated Step 1
Iter      SolEst     Damping    Stepsize #Res #Jac #Sol   LinErr   LinRes
   1      0.0018   1.0000000      0.0018  440  208  208  2.1e-14  7.6e-13
Solution error estimates for segregated groups
0.015, 0.37, 0.0018
Residual error estimates for segregated groups
27, 2.6e+02, 38
Segregated solver iteration 4.
Electric Currents
Iter      SolEst     Damping    Stepsize #Res #Jac #Sol   LinErr   LinRes
```

```
   1       0.012   1.0000000       0.012  442  209  209  8.3e-14  5.7e-11
Concentrations
Iter      SolEst      ResEst     Damping    Stepsize #Res #Jac #Sol   LinErr   LinRe
s
   1        0.14     1.7e+02   0.3500000        0.14 1022  580  580    1e-14  1.6e-
15
   2       0.093     1.1e+02   0.3500000       0.093 1023  581  581  2.8e-15  1.7e-
15
   3       0.061          73   0.3500000       0.061 1024  582  582  5.3e-15  1.7e-
15
Segregated Step 1
Iter      SolEst     Damping    Stepsize #Res #Jac #Sol   LinErr   LinRes
   1     0.00078   1.0000000     0.00078  442  209  209  1.9e-14  5.9e-13
Solution error estimates for segregated groups
0.012, 0.1, 0.00078
Residual error estimates for segregated groups
25, 73, 25
Segregated solver iteration 5.
Electric Currents
Iter      SolEst     Damping    Stepsize #Res #Jac #Sol   LinErr   LinRes
   1      0.0091   1.0000000      0.0091  444  210  210  1.2e-13  6.1e-11
Concentrations
Iter      SolEst      ResEst     Damping    Stepsize #Res #Jac #Sol   LinErr   LinRe
s
   1        0.04          48   0.3500000        0.04 1027  583  583  4.6e-15  1.7e-
15
   2       0.026          32   0.3500000       0.026 1028  584  584  5.8e-15  1.7e-
15
   3       0.017          22   0.3500000       0.017 1029  585  585  4.7e-15  1.7e-
15
Segregated Step 1
Iter      SolEst     Damping    Stepsize #Res #Jac #Sol   LinErr   LinRes
   1     0.00027   1.0000000     0.00027  444  210  210  9.6e-14  3.3e-13
Solution error estimates for segregated groups
0.0091, 0.029, 0.00027
Residual error estimates for segregated groups
28, 22, 15
Segregated solver iteration 6.
Electric Currents
Iter      SolEst     Damping    Stepsize #Res #Jac #Sol   LinErr   LinRes
   1      0.0064   1.0000000      0.0064  446  211  211  3.6e-14  4.5e-11
Concentrations
Iter      SolEst      ResEst     Damping    Stepsize #Res #Jac #Sol   LinErr   LinRe
s
   1       0.011          18   0.3500000       0.011 1032  586  586  1.3e-14  2.1e-
15
   2      0.0072          14   0.3500000      0.0072 1033  587  587  3.2e-14    2e-
15
   3      0.0047          11   0.3500000      0.0047 1034  588  588  5.1e-14    2e-
15
Segregated Step 1
Iter      SolEst     Damping    Stepsize #Res #Jac #Sol   LinErr   LinRes
   1      0.0003   1.0000000      0.0003  446  211  211  8.8e-14  6.5e-13
Solution error estimates for segregated groups
0.0064, 0.008, 0.0003
Residual error estimates for segregated groups
```

```
30, 11, 11
Segregated solver iteration 7.
Electric Currents
Iter      SolEst     Damping    Stepsize #Res #Jac #Sol   LinErr   LinRes
   1      0.0038   1.0000000      0.0038  448  212  212  4.1e-14  3.1e-11
Concentrations
Iter      SolEst      ResEst     Damping    Stepsize #Res #Jac #Sol   LinErr   LinRe
s
   1      0.0032          12   0.3500000      0.0032 1037  589  589  3.2e-14  4.2e-
15
   2      0.0021          11   0.3500000      0.0021 1038  590  590    6e-15  4.2e-
15
   3      0.0013         9.7   0.3500000      0.0013 1039  591  591  6.9e-15  4.1e-
15
Segregated Step 1
Iter      SolEst     Damping    Stepsize #Res #Jac #Sol   LinErr   LinRes
   1     0.00046   1.0000000     0.00046  448  212  212  5.3e-14  1.4e-12
Solution error estimates for segregated groups
0.0038, 0.0023, 0.00046
Residual error estimates for segregated groups
28, 9.7, 11
Segregated solver iteration 8.
Electric Currents
Iter      SolEst     Damping    Stepsize #Res #Jac #Sol   LinErr   LinRes
   1      0.0018   1.0000000      0.0018  450  213  213  2.1e-13  2.2e-11
Concentrations
Iter      SolEst      ResEst     Damping    Stepsize #Res #Jac #Sol   LinErr   LinRe
s
   1      0.0011          12   0.3500000      0.0011 1042  592  592  4.1e-14  1.1e-
14
   2     0.00074          11   0.3500000     0.00074 1043  593  593    3e-14  1.2e-
14
Segregated Step 1
Iter      SolEst     Damping    Stepsize #Res #Jac #Sol   LinErr   LinRes
   1     0.00052   1.0000000     0.00052  450  213  213  3.4e-14  2.1e-12
Solution error estimates for segregated groups
0.0018, 0.00074, 0.00052
Residual error estimates for segregated groups
20, 11, 16
Segregated solver iteration 9.
Electric Currents
Iter      SolEst     Damping    Stepsize #Res #Jac #Sol   LinErr   LinRes
   1     0.00036   1.0000000     0.00036  452  214  214  1.9e-13  1.8e-11
Concentrations
Iter      SolEst      ResEst     Damping    Stepsize #Res #Jac #Sol   LinErr   LinRe
s
   1     0.00075          12   0.3500000     0.00075 1046  594  594  1.4e-14  2.3e-
14
Segregated Step 1
Iter      SolEst     Damping    Stepsize #Res #Jac #Sol   LinErr   LinRes
   1     0.00042   1.0000000     0.00042  452  214  214  1.6e-14  1.9e-12
Solution error estimates for segregated groups
0.00036, 0.00049, 0.00042
Residual error estimates for segregated groups
11, 12, 13
```

```
Parameter Vmetal = -0.12.
Segregated solver iteration 1.
Electric Currents
Iter      SolEst     Damping    Stepsize #Res #Jac #Sol   LinErr   LinRes
   1        0.92   1.0000000        0.92  455  215  215  1.4e-13  1.4e-14
Concentrations
Iter      SolEst      ResEst     Damping    Stepsize #Res #Jac #Sol   LinErr   LinRe
s
   1         0.7     5.1e+03   0.3500000         0.7 1050  595  595  3.8e-15  1.6e-
15
   2        0.55     3.9e+03   0.3500000        0.55 1051  596  596  3.2e-15  1.6e-
15
   3        0.47     2.6e+03   0.3500000        0.47 1052  597  597  4.9e-15  1.6e-
15
Segregated Step 1
Iter      SolEst     Damping    Stepsize #Res #Jac #Sol   LinErr   LinRes
   1          21   1.0000000          21  455  215  215  9.1e-15  2.1e-15
Solution error estimates for segregated groups
0.92, 0.8, 21
Residual error estimates for segregated groups
1.7e+03, 9.5e+03, 9.5e+03
Segregated solver iteration 2.
Electric Currents
Iter      SolEst     Damping    Stepsize #Res #Jac #Sol   LinErr   LinRes
   1       0.016   1.0000000       0.016  457  216  216  3.3e-14  9.4e-12
Concentrations
Iter      SolEst      ResEst     Damping    Stepsize #Res #Jac #Sol   LinErr   LinRe
s
   1        0.42     2.3e+03   0.3500000        0.42 1055  598  598  3.7e-15  1.6e-
15
   2        0.39     1.5e+03   0.3500000        0.39 1056  599  599  6.6e-15  1.6e-
15
   3        0.37     9.5e+02   0.3500000        0.37 1057  600  600  3.6e-15  1.6e-
15
Segregated Step 1
Iter      SolEst     Damping    Stepsize #Res #Jac #Sol   LinErr   LinRes
   1      0.0032   1.0000000      0.0032  457  216  216  2.3e-14  5.7e-13
Solution error estimates for segregated groups
0.016, 0.63, 0.0032
Residual error estimates for segregated groups
54, 9.5e+02, 75
Segregated solver iteration 3.
Electric Currents
Iter      SolEst     Damping    Stepsize #Res #Jac #Sol   LinErr   LinRes
   1       0.014   1.0000000       0.014  459  217  217  9.9e-14  2.6e-11
Concentrations
Iter      SolEst      ResEst     Damping    Stepsize #Res #Jac #Sol   LinErr   LinRe
s
   1        0.35     6.2e+02   0.3500000        0.35 1060  601  601  2.9e-15  1.6e-
15
   2        0.31       4e+02   0.3500000        0.31 1061  602  602  6.1e-15  1.6e-
15
   3        0.21     2.6e+02   0.3500000        0.21 1062  603  603  3.3e-15  1.6e-
15
Segregated Step 1
Iter      SolEst     Damping    Stepsize #Res #Jac #Sol   LinErr   LinRes
```

```
   1      0.0018   1.0000000      0.0018  459  217  217  1.1e-14  7.2e-13
Solution error estimates for segregated groups
0.014, 0.36, 0.0018
Residual error estimates for segregated groups
29, 2.6e+02, 38
Segregated solver iteration 4.
Electric Currents
Iter      SolEst     Damping    Stepsize #Res #Jac #Sol   LinErr   LinRes
   1       0.012   1.0000000       0.012  461  218  218  1.7e-13  5.5e-11
Concentrations
Iter      SolEst      ResEst     Damping    Stepsize #Res #Jac #Sol   LinErr   LinRe
s
   1        0.14     1.7e+02   0.3500000        0.14 1065  604  604  4.3e-15  1.6e-
15
   2       0.091     1.1e+02   0.3500000       0.091 1066  605  605  3.3e-15  1.7e-
15
   3        0.06          72   0.3500000        0.06 1067  606  606  5.1e-15  1.6e-
15
Segregated Step 1
Iter      SolEst     Damping    Stepsize #Res #Jac #Sol   LinErr   LinRes
   1     0.00083   1.0000000     0.00083  461  218  218  1.7e-14  5.9e-13
Solution error estimates for segregated groups
0.012, 0.1, 0.00083
Residual error estimates for segregated groups
24, 72, 26
Segregated solver iteration 5.
Electric Currents
Iter      SolEst     Damping    Stepsize #Res #Jac #Sol   LinErr   LinRes
   1      0.0095   1.0000000      0.0095  463  219  219  7.2e-14  6.2e-11
Concentrations
Iter      SolEst      ResEst     Damping    Stepsize #Res #Jac #Sol   LinErr   LinRe
s
   1       0.039          48   0.3500000       0.039 1070  607  607  4.1e-15  1.7e-
15
   2       0.025          32   0.3500000       0.025 1071  608  608  5.5e-15  1.7e-
15
   3       0.017          21   0.3500000       0.017 1072  609  609  1.1e-14  1.7e-
15
Segregated Step 1
Iter      SolEst     Damping    Stepsize #Res #Jac #Sol   LinErr   LinRes
   1     0.00031   1.0000000     0.00031  463  219  219  7.9e-15  3.5e-13
Solution error estimates for segregated groups
0.0095, 0.028, 0.00031
Residual error estimates for segregated groups
27, 21, 16
Segregated solver iteration 6.
Electric Currents
Iter      SolEst     Damping    Stepsize #Res #Jac #Sol   LinErr   LinRes
   1       0.007   1.0000000       0.007  465  220  220  3.3e-13  4.9e-11
Concentrations
Iter      SolEst      ResEst     Damping    Stepsize #Res #Jac #Sol   LinErr   LinRe
s
   1       0.011          18   0.3500000       0.011 1075  610  610  4.5e-15  2.1e-
15
   2       0.007          14   0.3500000       0.007 1076  611  611  1.2e-14    2e-
15
```

```
   3      0.0046          11   0.3500000      0.0046 1077  612  612  8.3e-15    2e-
15
Segregated Step 1
Iter      SolEst     Damping    Stepsize #Res #Jac #Sol   LinErr   LinRes
   1     0.00027   1.0000000     0.00027  465  220  220  2.9e-14  5.4e-13
Solution error estimates for segregated groups
0.007, 0.0079, 0.00027
Residual error estimates for segregated groups
30, 11, 12
Segregated solver iteration 7.
Electric Currents
Iter      SolEst     Damping    Stepsize #Res #Jac #Sol   LinErr   LinRes
   1      0.0047   1.0000000      0.0047  467  221  221  3.3e-13  3.5e-11
Concentrations
Iter      SolEst      ResEst     Damping    Stepsize #Res #Jac #Sol   LinErr   LinRe
s
   1      0.0031          13   0.3500000      0.0031 1080  613  613  9.7e-15  4.5e-
15
   2       0.002          11   0.3500000       0.002 1081  614  614  2.1e-14  4.4e-
15
   3      0.0013         9.8   0.3500000      0.0013 1082  615  615  6.2e-15  4.3e-
15
Segregated Step 1
Iter      SolEst     Damping    Stepsize #Res #Jac #Sol   LinErr   LinRes
   1     0.00044   1.0000000     0.00044  467  221  221  4.6e-14  1.2e-12
Solution error estimates for segregated groups
0.0047, 0.0023, 0.00044
Residual error estimates for segregated groups
29, 9.8, 11
Segregated solver iteration 8.
Electric Currents
Iter      SolEst     Damping    Stepsize #Res #Jac #Sol   LinErr   LinRes
   1      0.0026   1.0000000      0.0026  469  222  222  1.5e-13  2.5e-11
Concentrations
Iter      SolEst      ResEst     Damping    Stepsize #Res #Jac #Sol   LinErr   LinRe
s
   1      0.0012          13   0.3500000      0.0012 1085  616  616  1.3e-14  1.3e-
14
   2     0.00079          11   0.3500000     0.00079 1086  617  617  1.5e-14  1.4e-
14
Segregated Step 1
Iter      SolEst     Damping    Stepsize #Res #Jac #Sol   LinErr   LinRes
   1     0.00053   1.0000000     0.00053  469  222  222  1.6e-14  1.8e-12
Solution error estimates for segregated groups
0.0026, 0.00079, 0.00053
Residual error estimates for segregated groups
22, 11, 17
Segregated solver iteration 9.
Electric Currents
Iter      SolEst     Damping    Stepsize #Res #Jac #Sol   LinErr   LinRes
   1     0.00087   1.0000000     0.00087  471  223  223  6.7e-14  1.9e-11
Concentrations
Iter      SolEst      ResEst     Damping    Stepsize #Res #Jac #Sol   LinErr   LinRe
s
   1     0.00087          12   0.3500000     0.00087 1089  618  618  3.5e-14  2.7e-
14
```

```
Segregated Step 1
Iter      SolEst     Damping    Stepsize #Res #Jac #Sol   LinErr   LinRes
   1     0.00046   1.0000000     0.00046  471  223  223  1.8e-14  1.8e-12
Solution error estimates for segregated groups
0.00087, 0.00057, 0.00046
Residual error estimates for segregated groups
13, 12, 14

Parameter Vmetal = -0.13.
Segregated solver iteration 1.
Electric Currents
Iter      SolEst     Damping    Stepsize #Res #Jac #Sol   LinErr   LinRes
   1        0.92   1.0000000        0.92  474  224  224  7.8e-14  1.4e-14
Concentrations
Iter      SolEst      ResEst     Damping    Stepsize #Res #Jac #Sol   LinErr   LinRe
s
   1        0.72     5.1e+03   0.3500000        0.72 1093  619  619    5e-15  1.6e-
15
   2        0.55     3.9e+03   0.3500000        0.55 1094  620  620  5.3e-15  1.6e-
15
   3        0.46     2.5e+03   0.3500000        0.46 1095  621  621  2.4e-15  1.6e-
15
Segregated Step 1
Iter      SolEst     Damping    Stepsize #Res #Jac #Sol   LinErr   LinRes
   1          22   1.0000000          22  474  224  224  3.5e-15  2.1e-15
Solution error estimates for segregated groups
0.92, 0.79, 22
Residual error estimates for segregated groups
1.8e+03, 9.5e+03, 9.5e+03
Segregated solver iteration 2.
Electric Currents
Iter      SolEst     Damping    Stepsize #Res #Jac #Sol   LinErr   LinRes
   1       0.014   1.0000000       0.014  476  225  225  6.8e-14  8.8e-12
Concentrations
Iter      SolEst      ResEst     Damping    Stepsize #Res #Jac #Sol   LinErr   LinRe
s
   1        0.42     2.2e+03   0.3500000        0.42 1098  622  622  5.4e-15  1.6e-
15
   2        0.39     1.5e+03   0.3500000        0.39 1099  623  623  1.4e-15  1.6e-
15
   3        0.36     9.4e+02   0.3500000        0.36 1100  624  624  3.5e-15  1.6e-
15
Segregated Step 1
Iter      SolEst     Damping    Stepsize #Res #Jac #Sol   LinErr   LinRes
   1      0.0032   1.0000000      0.0032  476  225  225  2.4e-14  5.3e-13
Solution error estimates for segregated groups
0.014, 0.62, 0.0032
Residual error estimates for segregated groups
55, 9.4e+02, 67
Segregated solver iteration 3.
Electric Currents
Iter      SolEst     Damping    Stepsize #Res #Jac #Sol   LinErr   LinRes
   1       0.014   1.0000000       0.014  478  226  226  8.1e-14  2.5e-11
Concentrations
Iter      SolEst      ResEst     Damping    Stepsize #Res #Jac #Sol   LinErr   LinRe
s
```

```
   1        0.34     6.1e+02   0.3500000        0.34 1103  625  625  4.3e-15  1.6e-
15
   2         0.3       4e+02   0.3500000         0.3 1104  626  626  3.2e-15  1.6e-
15
   3        0.21     2.6e+02   0.3500000        0.21 1105  627  627  3.9e-15  1.6e-
15
Segregated Step 1
Iter      SolEst     Damping    Stepsize #Res #Jac #Sol   LinErr   LinRes
   1      0.0018   1.0000000      0.0018  478  226  226  9.1e-15  6.8e-13
Solution error estimates for segregated groups
0.014, 0.35, 0.0018
Residual error estimates for segregated groups
31, 2.6e+02, 37
Segregated solver iteration 4.
Electric Currents
Iter      SolEst     Damping    Stepsize #Res #Jac #Sol   LinErr   LinRes
   1       0.012   1.0000000       0.012  480  227  227  2.9e-13  5.3e-11
Concentrations
Iter      SolEst      ResEst     Damping    Stepsize #Res #Jac #Sol   LinErr   LinRe
s
   1        0.14     1.7e+02   0.3500000        0.14 1108  628  628  7.3e-15  1.6e-
15
   2       0.089     1.1e+02   0.3500000       0.089 1109  629  629  5.2e-15  1.6e-
15
   3       0.058          72   0.3500000       0.058 1110  630  630    4e-15  1.6e-
15
Segregated Step 1
Iter      SolEst     Damping    Stepsize #Res #Jac #Sol   LinErr   LinRes
   1     0.00087   1.0000000     0.00087  480  227  227  1.6e-14  5.8e-13
Solution error estimates for segregated groups
0.012, 0.1, 0.00087
Residual error estimates for segregated groups
25, 72, 27
Segregated solver iteration 5.
Electric Currents
Iter      SolEst     Damping    Stepsize #Res #Jac #Sol   LinErr   LinRes
   1      0.0096   1.0000000      0.0096  482  228  228  2.4e-13  6.2e-11
Concentrations
Iter      SolEst      ResEst     Damping    Stepsize #Res #Jac #Sol   LinErr   LinRe
s
   1       0.038          47   0.3500000       0.038 1113  631  631  6.8e-15  1.7e-
15
   2       0.025          32   0.3500000       0.025 1114  632  632  4.1e-15  1.7e-
15
   3       0.016          21   0.3500000       0.016 1115  633  633  5.1e-15  1.6e-
15
Segregated Step 1
Iter      SolEst     Damping    Stepsize #Res #Jac #Sol   LinErr   LinRes
   1     0.00036   1.0000000     0.00036  482  228  228  5.6e-14  3.6e-13
Solution error estimates for segregated groups
0.0096, 0.028, 0.00036
Residual error estimates for segregated groups
26, 21, 17
Segregated solver iteration 6.
Electric Currents
Iter      SolEst     Damping    Stepsize #Res #Jac #Sol   LinErr   LinRes
```

```
   1      0.0075   1.0000000      0.0075  484  229  229  5.1e-14  5.2e-11
Concentrations
Iter      SolEst      ResEst     Damping    Stepsize #Res #Jac #Sol   LinErr   LinRe
s
   1       0.011          17   0.3500000       0.011 1118  634  634  1.1e-14  2.1e-
15
   2      0.0069          14   0.3500000      0.0069 1119  635  635  1.6e-14    2e-
15
   3      0.0045          11   0.3500000      0.0045 1120  636  636  7.1e-15    2e-
15
Segregated Step 1
Iter      SolEst     Damping    Stepsize #Res #Jac #Sol   LinErr   LinRes
   1     0.00025   1.0000000     0.00025  484  229  229  3.2e-14  4.5e-13
Solution error estimates for segregated groups
0.0075, 0.0077, 0.00025
Residual error estimates for segregated groups
29, 11, 12
Segregated solver iteration 7.
Electric Currents
Iter      SolEst     Damping    Stepsize #Res #Jac #Sol   LinErr   LinRes
   1      0.0054   1.0000000      0.0054  486  230  230  8.8e-14  3.9e-11
Concentrations
Iter      SolEst      ResEst     Damping    Stepsize #Res #Jac #Sol   LinErr   LinRe
s
   1      0.0031          13   0.3500000      0.0031 1123  637  637  9.9e-15  4.6e-
15
   2       0.002          12   0.3500000       0.002 1124  638  638  1.9e-14  4.5e-
15
   3      0.0013         9.8   0.3500000      0.0013 1125  639  639  2.5e-14  4.4e-
15
Segregated Step 1
Iter      SolEst     Damping    Stepsize #Res #Jac #Sol   LinErr   LinRes
   1     0.00041   1.0000000     0.00041  486  230  230  2.8e-14    1e-12
Solution error estimates for segregated groups
0.0054, 0.0022, 0.00041
Residual error estimates for segregated groups
30, 9.8, 10
Segregated solver iteration 8.
Electric Currents
Iter      SolEst     Damping    Stepsize #Res #Jac #Sol   LinErr   LinRes
   1      0.0034   1.0000000      0.0034  488  231  231  1.9e-13  2.8e-11
Concentrations
Iter      SolEst      ResEst     Damping    Stepsize #Res #Jac #Sol   LinErr   LinRe
s
   1      0.0013          13   0.3500000      0.0013 1128  640  640    4e-14  1.5e-
14
   2     0.00082          12   0.3500000     0.00082 1129  641  641  3.1e-14  1.5e-
14
Segregated Step 1
Iter      SolEst     Damping    Stepsize #Res #Jac #Sol   LinErr   LinRes
   1     0.00052   1.0000000     0.00052  488  231  231  2.1e-14  1.6e-12
Solution error estimates for segregated groups
0.0034, 0.00082, 0.00052
Residual error estimates for segregated groups
24, 12, 18
Segregated solver iteration 9.
```

```
Electric Currents
Iter      SolEst     Damping    Stepsize #Res #Jac #Sol   LinErr   LinRes
   1      0.0015   1.0000000      0.0015  490  232  232  1.6e-13  2.2e-11
Concentrations
Iter      SolEst      ResEst     Damping    Stepsize #Res #Jac #Sol   LinErr   LinRe
s
   1     0.00097          13   0.3500000     0.00097 1132  642  642  9.6e-15    3e-
14
Segregated Step 1
Iter      SolEst     Damping    Stepsize #Res #Jac #Sol   LinErr   LinRes
   1     0.00048   1.0000000     0.00048  490  232  232  4.8e-14  1.7e-12
Solution error estimates for segregated groups
0.0015, 0.00063, 0.00048
Residual error estimates for segregated groups
14, 13, 14
Segregated solver iteration 10.
Electric Currents
Iter      SolEst     Damping    Stepsize #Res #Jac #Sol   LinErr   LinRes
   1     0.00043   1.0000000     0.00043  492  233  233  2.2e-13  1.9e-11
Concentrations
Iter      SolEst      ResEst     Damping    Stepsize #Res #Jac #Sol   LinErr   LinRe
s
   1      0.0009          13   0.3500000      0.0009 1135  643  643  1.5e-14  4.5e-
14
Segregated Step 1
Iter      SolEst     Damping    Stepsize #Res #Jac #Sol   LinErr   LinRes
   1     0.00031   1.0000000     0.00031  492  233  233  8.4e-15  1.2e-12
Solution error estimates for segregated groups
0.00043, 0.00059, 0.00031
Residual error estimates for segregated groups
12, 13, 10
Solution time: 48518 s. (13 hours, 28 minutes, 38 seconds)
Physical memory: 34.23 GB
Virtual memory: 41.33 GB
Ended at Sep 24, 2025, 6:10:15 AM.
----- Stationary Solver 1 in Study 1/Solution 1 (sol1) ------------------------>
```

Advanced (aDef)

ASSEMBLY SETTINGS

Description	Value
Reuse sparsity pattern	On

Parametric 1 (p1)

GENERAL

Description	Value
Defined by study step	Parametric Sweep
Run continuation for	No parameter
On error	Skip parameter step

PARAMETERS

Parameter name	Parameter value list	Parameter unit
Vmetal	range(0.13,-0.01,0.01),-1e-6, range(-0.01,-0.01,-0.13)	V

Segregated 1 (se1)

GENERAL

Description	Value
Maximum number of iterations	1000

Electric Currents (ss1)

GENERAL

Description	Value
Variables	Electric Potential (comp1.V)
Linear solver	Direct

Concentrations (ss2)

GENERAL

Description	Value
Variables	{Concentration (comp1.cAu), Concentration (comp1.cH)}
Linear solver	Direct

METHOD AND TERMINATION

Description	Value
Damping factor	0.35
Termination technique	Iterations or tolerance
Number of iterations	3
Tolerance factor	1

Segregated Step 1 (tds1)

GENERAL

Description	Value
Variables	{Concentration (comp1.cCu), Concentration (comp1.cSO4)}
Linear solver	Direct

4 Results

4.1 DATASETS

4.1.1 Study 1/Solution 1

SOLUTION

Description	Value
Solution	Solution 1 (sol1)
Component	Component 1 (comp1)

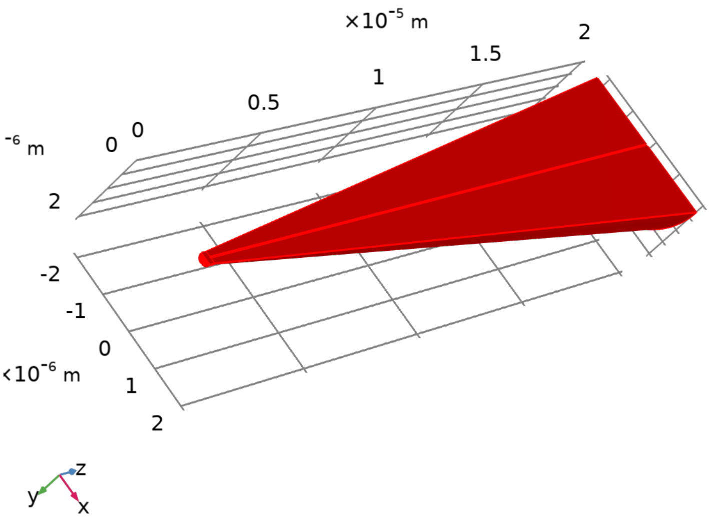

Dataset: Study 1/Solution 1

4.2 DERIVED VALUES

4.2.1 Average voltage

DATA

Description	Value
Dataset	Study 1/Solution 1 (sol1)

EXPRESSIONS

Expression	Unit	Description
V	V	Electric potential

INTEGRATION SETTINGS

Description	Value
Integration order	4

4.2.2 Surface Integration Cu

OUTPUT

Evaluated in	Table 1

DATA

Description	Value
Dataset	Study 1/Solution 1 (sol1)

EXPRESSIONS

Expression	Unit	Description
tds.ntflux_cCu	mol/s	Normal total flux

INTEGRATION SETTINGS

Description	Value
Integration order	4

4.2.3 Surface Integration Au

OUTPUT

Evaluated in	Table 2

DATA

Description	Value
Dataset	Study 1/Solution 1 (sol1)

EXPRESSIONS

Expression	Unit	Description
tds.ntflux_cAu	mol/s	Normal total flux

INTEGRATION SETTINGS

Description	Value
Integration order	4

4.3 TABLES

4.3.1 Table 1

Surface Integration Cu

Vmetal (V)	Normal total flux (mol/s)
0.13	6.5427E-16
0.12	6.137E-16

Vmetal (V)	Normal total flux (mol/s)
0.11	5.7185E-16
0.1	5.2865E-16
0.09	4.8403E-16
0.08	4.3809E-16
0.07	3.9081E-16
0.06	3.4235E-16
0.05	2.9314E-16
0.04	2.4394E-16
0.03	1.9595E-16
0.02	1.5083E-16
0.01	1.105E-16
-1E-6	7.6678E-17
-0.01	5.0324E-17
-0.02	3.1317E-17
-0.03	1.8606E-17
-0.04	1.0665E-17
-0.05	5.989E-18
-0.06	3.3693E-18
-0.07	1.9643E-18
-0.08	1.2422E-18
-0.09	8.8981E-19
-0.1	7.3172E-19
-0.11	6.7322E-19
-0.12	6.6453E-19
-0.13	6.8041E-19

4.3.2 Table 2

Surface Integration Au

Vmetal (V)	Normal total flux (mol/s)
0.13	4.3081E-18
0.12	5.6827E-18
0.11	7.5597E-18
0.1	1.0096E-17
0.09	1.3485E-17
0.08	1.7961E-17

Vmetal (V)	Normal total flux (mol/s)
0.07	2.3799E-17
0.06	3.1306E-17
0.05	4.0814E-17
0.04	5.2661E-17
0.03	6.7161E-17
0.02	8.4586E-17
0.01	1.0513E-16
-1E-6	1.2887E-16
-0.01	1.5579E-16
-0.02	1.8574E-16
-0.03	2.1846E-16
-0.04	2.5362E-16
-0.05	2.9078E-16
-0.06	3.2957E-16
-0.07	3.6955E-16
-0.08	4.1034E-16
-0.09	4.5161E-16
-0.1	4.9325E-16
-0.11	5.3473E-16
-0.12	5.7602E-16
-0.13	6.1713E-16

4.4 PLOT GROUPS

4.4.1 Electric Potential (ec)

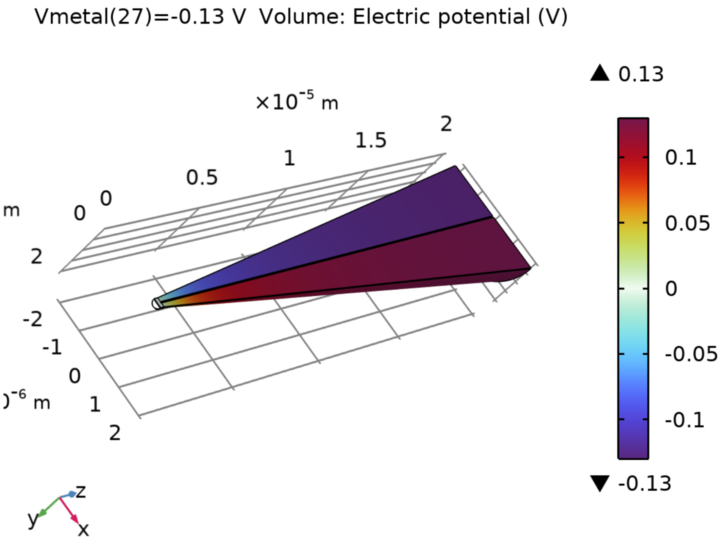

Volume: Electric potential (V)

4.4.2 Electric Field Norm (ec)

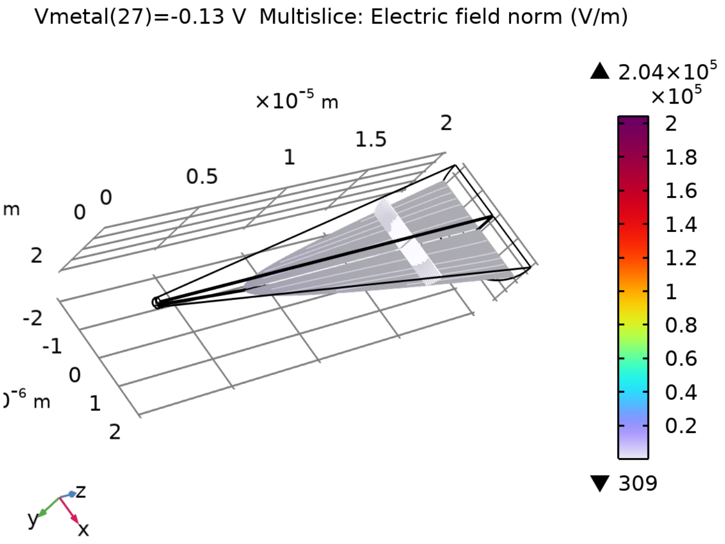

Multislice: Electric field norm (V/m)

4.4.3 Concentration, Au, Streamline (tds)

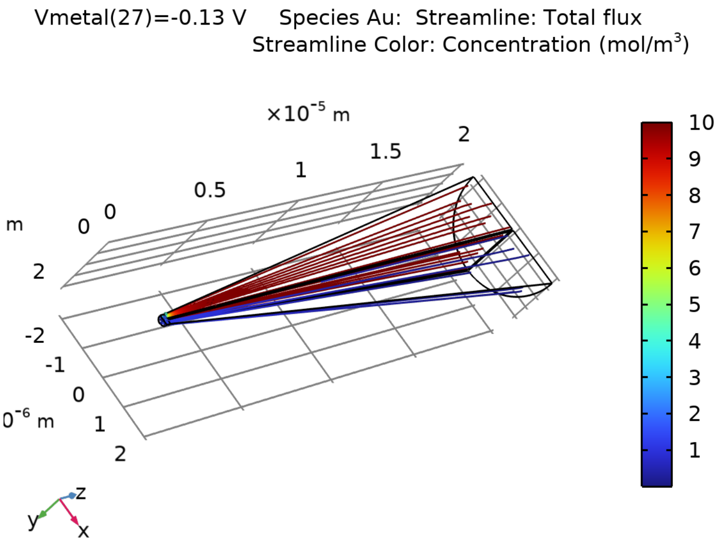

Species Au: Streamline: Total flux   Streamline Color: Concentration (mol/m³)

4.4.4 Concentration, Au, Surface (tds)

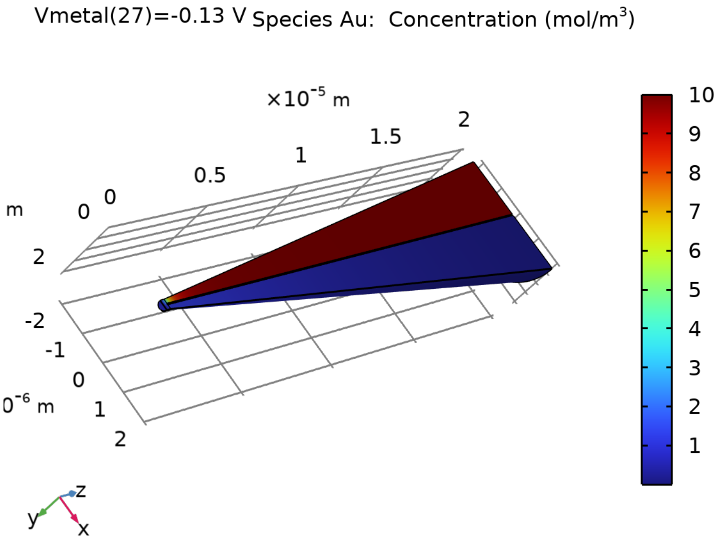

Species Au: Concentration (mol/m³)

4.4.5 Concentration, Pt, Streamline (tds)

4.4.6 Concentration, Pt, Surface (tds)

4.4.7 Concentration, H, Streamline (tds)

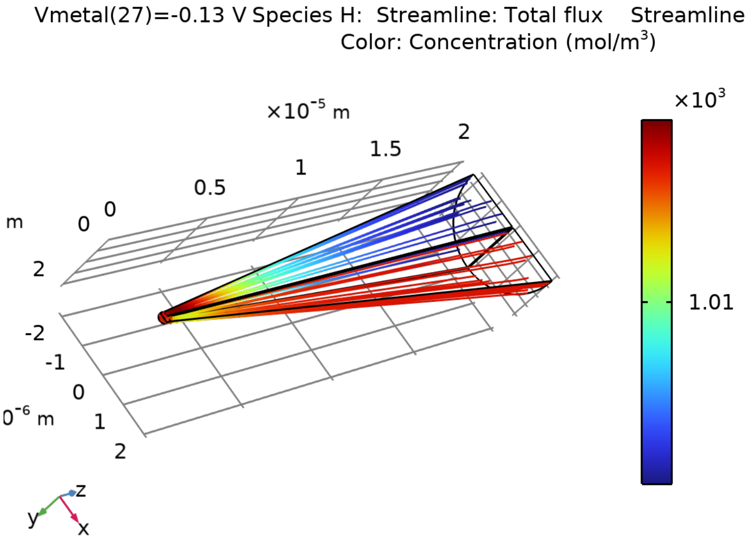

Species H: Streamline: Total flux Streamline Color: Concentration (mol/m^3)

4.4.8 Concentration, H, Surface (tds)

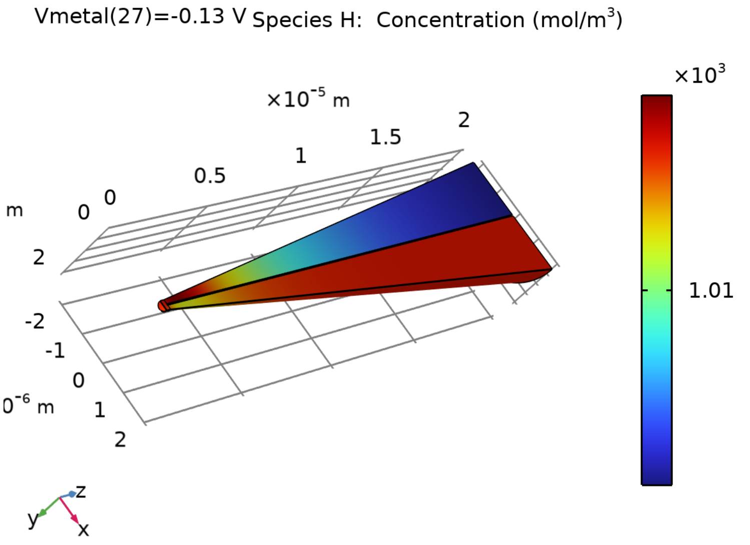

Species H: Concentration (mol/m^3)

4.4.9 1D Plot Group 9

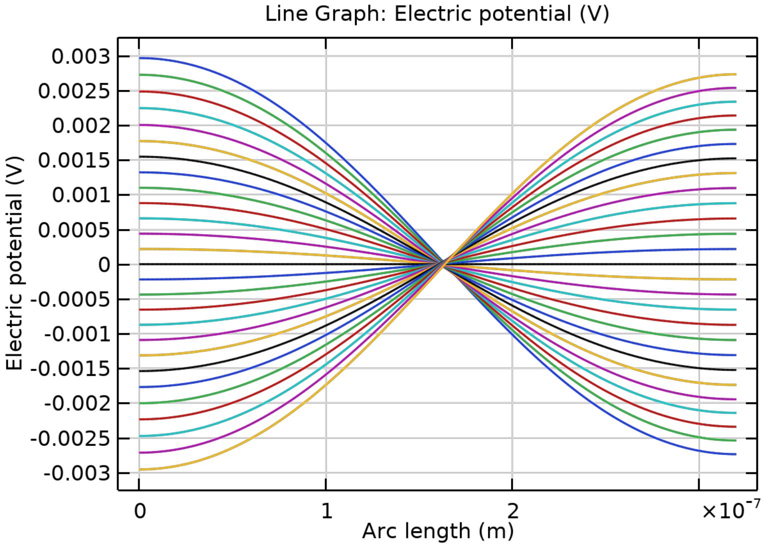

Line Graph: Electric potential (V)

4.4.10 1D Plot Group 10

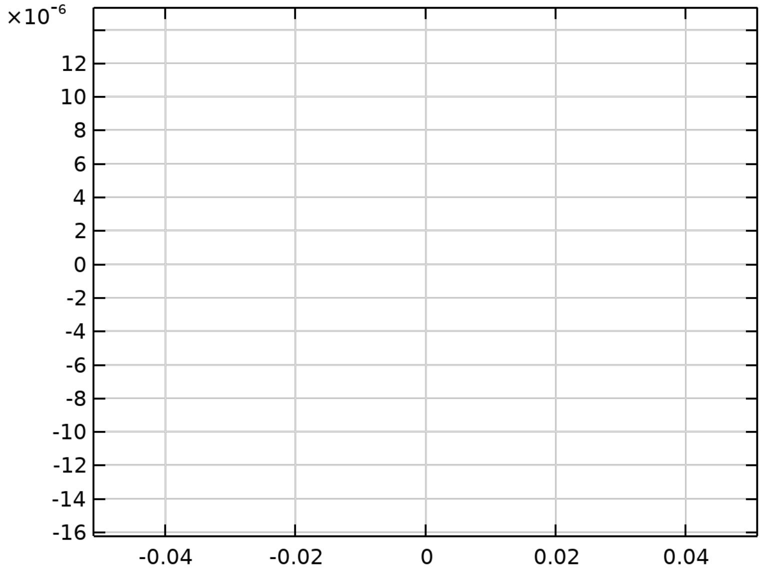

4.4.11 1D Plot Group 11

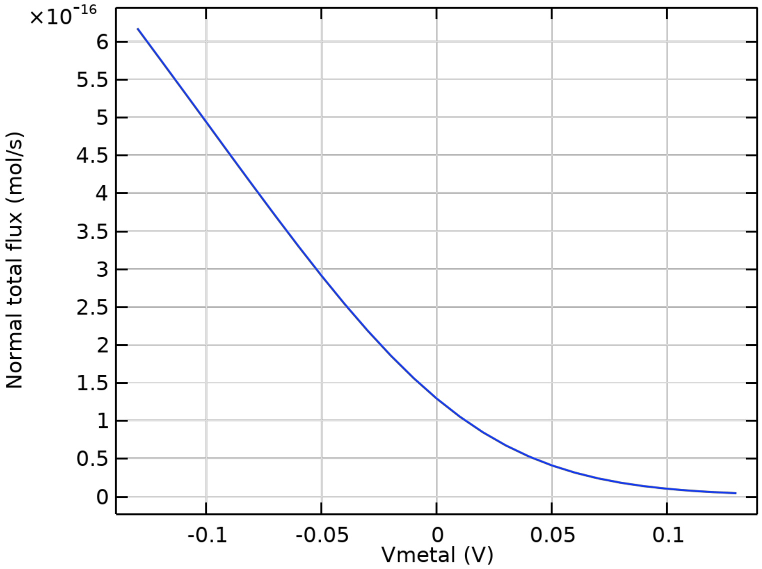

Supplement: Supplementary file 2 [file nn5c16931_si_002.pdf]
